# A Broad Set of Chromatin Factors Influences Splicing

**DOI:** 10.1371/journal.pgen.1006318

**Published:** 2016-09-23

**Authors:** Eric Allemand, Michael P. Myers, Jose Garcia-Bernardo, Annick Harel-Bellan, Adrian R. Krainer, Christian Muchardt

**Affiliations:** 1 Institut Pasteur, Unit of Epigenetic regulation, Paris, France; 2 CNRS, UMR3738, Paris, France; 3 ICGEB Trieste, Protein Networks unit, Trieste, Italy; 4 Institute for Integrative Biology of the Cell (I2BC), IBiTecs, CEA, CNRS, Gif-sur-Yvette, France; 5 Université Paris Saclay, Gif-sur-Yvette, France; 6 Cold Spring Harbor Laboratory, Cold Spring Harbor, New York, United States of America; Universitat Pompeu Fabra, SPAIN

## Abstract

Several studies propose an influence of chromatin on pre-mRNA splicing, but it is still unclear how widespread and how direct this phenomenon is. We find here that when assembled *in vivo*, the U2 snRNP co-purifies with a subset of chromatin-proteins, including histones and remodeling complexes like SWI/SNF. Yet, an unbiased RNAi screen revealed that the outcome of splicing is influenced by a much larger variety of chromatin factors not all associating with the spliceosome. The availability of this broad range of chromatin factors impacting splicing further unveiled their very context specific effect, resulting in either inclusion or skipping, depending on the exon under scrutiny. Finally, a direct assessment of the impact of chromatin on splicing using an *in vitro* co-transcriptional splicing assay with pre-mRNAs transcribed from a nucleosomal template, demonstrated that chromatin impacts nascent pre-mRNP in their competence for splicing. Altogether, our data show that numerous chromatin factors associated or not with the spliceosome can affect the outcome of splicing, possibly as a function of the local chromatin environment that by default interferes with the efficiency of splicing.

## Author Summary

Splicing is an RNA editing step allowing to produce multiple transcripts from a single gene. The gene itself is organized in chromatin, associating DNA and multiple proteins. Some proteins regulating the compaction of the chromatin also affect RNA splicing. Yet, it was unclear whether these chromatin proteins were exceptions or whether chromatin very generally affected the outcome of splicing. Here, we show that a subset of chromatin proteins is physically in interaction with the enzyme responsible for RNA splicing. In addition, several chromatin proteins not found directly associated with the splicing machinery were also able to influence RNA splicing, suggesting that chromatin compaction very globally plays a role in splicing. This finding was confirmed using the first *in vitro* assay combining transcription and splicing in the context of chromatin; this assay showed that assembling DNA with chromatin proteins influences the efficiency of splicing.

## Introduction

The transcribed region of almost all human genes contains introns that must be excised from the pre-mRNA for the exons to be spliced together. This process provides an opportunity to modify the exon content of the mature mRNA and, as such, must be regarded as a powerful source of RNA diversity. This alternative splicing is highly regulated and largely depends on a number of non-snRNP splicing factors that bind site-specifically to sequences present on the pre-mRNAs.

Over the recent years, a series of observations has suggested that splicing is also influenced by histone modifying enzymes, readers of histone modifications, and chromatin remodelers such as SWI/SNF. Here, we will refer to these proteins as chromatin factors. The reasoning behind a possible impact of these factors on splicing is that splicing is mostly co-transcriptional, and therefore potentially influenced by proteins associated with the transcribed template.

At least three modifications of histone H3 present inside the coding region of genes, namely tri-methylation of H3 at lysines 9 (H3K9), 27 (H3K27), and 36 (H3K36), were shown to affect the outcome of splicing in mammalian cells through their specific recognition by dedicated chromatin factors [1–4]. A role for intragenic DNA methylation has also been advocated either as a modification interfering with the recruitment of the boundary protein CTCF that in turns affects splicing, or as a booster of H3K9 tri-methylation [5,6].

H3K4 tri-methylation, a modification tightly associated with transcription start sites, and CHD1, a chromatin remodeler able to bind this modification have also been linked to the regulation of splicing [7]. That study showed the first interaction between a chromatin factor (CHD1) and components of the U2 snRNP, and suggested that this snRNP may function as a bridge between chromatin and splicing machineries. Other observations have since given support to that idea. In particular, immuno-purification of the splicing factor PRP40A from HeLa cell nuclear extracts brings down U2 snRNP subunits together with SWI/SNF subunits and several CHD family members [8]. Furthermore, experiments in *Schizosaccharomyces pombe* have revealed genetic interactions between U2 and SWI/SNF subunits [9]. Finally, the U2 snRNP subunit SF3B1 was shown to interact directly with chromatin [10], with Polycomb group proteins [11], and with the WSTF-SNF2h chromatin remodeling complex [12].

The U2 snRNP is composed of the U2 snRNA and numerous proteins, including 7 Sm proteins, U2-A’, U2-B”, and the components of the SF3A and SF3B complexes. It associates with the lariat branch site near the 3’ end of the intron *via* base-pairing between the U2 snRNA and the pre-mRNA. This binding is primed by the association of the U1 snRNP to the 5’ end of the pre-mRNA and the binding of SF1 and U2AF to the branch site and the polypyrimidine track, respectively.

The U1 and U2 snRNPs together with the pre-mRNA form the A complex or pre-spliceosome. In most cases, the positioning of this complex defines the exon-intron borders or splice-sites that will be used [13].

The A complex then associates with the U4/U5/U6 tri-snRNP, and finally U1 and U4 are evicted to generate an active B complex. This complex catalyzes a first transesterification reaction that cleaves between the upstream exon and the intron.

Finally, the splicing reaction is complete by a second transesterification reaction that rejoins the two exons and releases the intron as a lariat [14].

In the present study, we wished to investigate to what extend the U2 snRNP was a pivot in connecting splicing to chromatin. To address this issue, we developed several complementary approaches. First, we captured spliceosomes assembled before the second transesterification reaction and showed the presence within this complex of chromatin and transcription factors. In a second inverse approach, we systematically depleted human tissue culture cells from known chromatin factors and examined the impact on a splicing reporter. Finally, we combined for the first time chromatin, transcription, and splicing in a same *in vitro* reaction to estimate the direct impact of chromatin on the splicing reaction. Together, our observation documents a direct and extensive impact of chromatin factors on splicing with however an outcome that remains difficult to predict possibly because of the influence of chromatin.

## Results

### U2 snRNP anchored to spliceosomes in the cell captures chromatin components

U2 is the only snRNP present in every spliceosome complexe. Therefore, to capture chromatin factors associated with the spliceosome in the course of an *in vivo* splicing reaction, we developed a new procedure for proteomic analysis of the U2 snRNP.

Earlier protocols for the purification of spliceosomes mostly relied on *in vitro* assembly of the splicing machinery on a tagged reporter RNA. The subsequent capture of the tagged RNA did not result in co-purification of any chromatin factor, possibly because, by design, the approach captured only spliceosomes assembled independently of transcription.

**Fig 1:**
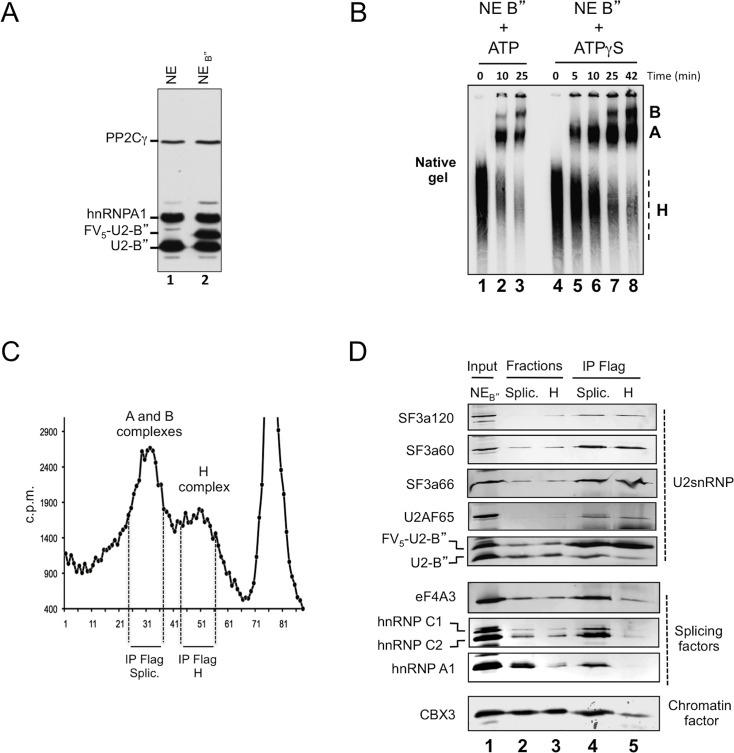
Purification of spliceosome complexes associated with the U2 snRNP. (A) Detection by western blot of indicated splicing factors in nuclear extracts from HeLa S3 cells either WT (NE) or stably transduced with FV_5_-U2-B (NE_B”_). (B) Comparison of the kinetic and quality of spliceosome assembly on a radiolabeled AdML pre-mRNA reporter in the presence of either ATP (lanes 1–3) or ATP-γS (lanes 4–8). Spliceosome complexes A and B were resolved from unspecific RNP (H complex) on a native gel. (C) Spliceosome complexes were purified by Sephacryl-S500 gel filtration from an *in vitro*-splicing reaction composed of NE_B”_ and radiolabeled AdML reporter incubated for 45 min in the presence of ATP-γS. Fractions with spliceosome and H complexes selected for anti-FLAG immunoprecipitation are indicated. Degraded RNAs and free proteins appear in fractions 68 to 80. (D) Spliceosome complexes anchored to the U2 snRNP. NE_B”_, gel-filtration fractions with either spliceosome (Splic.; lane 2) or H complex (H; lane 3), and the product of the anti-FLAG immunoprecipitation (lanes 4 and 5) were analyzed by western blot using the indicated antibodies. See also Table 1 and S1D and S1E Fig.

Here, to capture *in vivo*-assembled U2 complexes, we engineered HeLa S3 cells to express a FLAG-V5-tagged version of U2-B” (FV_5_-U2-B”), a constitutive component of the U2 snRNP (Fig 1A). Immuno-purifications in the absence of any cross-linking showed that the recombinant FV_5_-U2-B” was incorporated into both the 12*S* and 17*S* forms of U2 snRNP (S1A Fig and S1B Fig). Nuclear extracts prepared from the FV_5_-U2-B”-expressing cells (NE_B”_) also retained full competence for splicing of a ^32^P-labelled AdML reporter pre-mRNA *in vitro* (S1C Fig compare lanes 1–3 and 6–8), and immuno-precipitation of FV_5_-U2-B” from the *in vitro* splicing reactions led to enrichment in both un-spliced and spliced AdML reporter RNA (S1C Fig, lanes 9–10), consistent with the presence of the U2 snRNP in all intermediate complexes of spliceosome assembly.

We then set up to examine the protein composition of complexes associated with FV_5_-U2-B” during the splicing reaction. To accumulate pre-spliceosome (complex A) and spliceosome (complex B), NE_B”_ was supplemented with ATPγS, an ATP analog which blocks the splicing reaction before the second transesterification step [15]. To facilitate the tracing of splicing complexes engaged in splicing reactions, NE_B”_ with ATPγS was further incubated for 40 min. at 30°C with ^32^P-labelled AdML reporter pre-mRNA. This tracing allowed to confirm accumulation of complexes A and B in our experimental conditions (Fig 1B).

The A and B complexes assembled *in vivo* on non-radioactive pre-mRNAs together with those assembled *in vitro* on the tracer pre-mRNA were resolved from non-specific ribonucleoparticles (H complex) by gel-filtration chromatography and used for immuno-purification with anti-FLAG antibody (Fig 1C).

As revealed by mass spectrometry, this procedure resulted in isolation of most previously characterized splicing factors (187 out of 284), including all the core components of the spliceosome and many regulators of splicing (S1 Table). In addition, the FV_5_-U2-B”-associated complexes contained a large number of chromatin factors (Table 1).

**Table 1:** Chromatin factors identified by mass spectrometry as associated physically with the spliceosome.

		Gene name		NCBI Gene Id.	Mr (kD)	Unique peptide	% coverage	log(e)	
**Chromatin Remodelling Factors**	**PBRM1**		*BAF180*	55193	181	27	24	-307.4	
	**SMARCC2**		*BAF170*	6601	132.8	23	36	-263.5	
	**SMARCA4**		*BRG1*	6597	188	21	23	-224.6	
	**SMARCD1**		*BAF60A*	6602	58.2	11	34	-135.3	
	**BRD7**		*BRD7*	29117	74.1	10	21	-97.3	
	**SMARCC1**		*BAF155*	6599	122.8	10	20	-183.8	
	**CHD4**		*CHD4*	1108	217.9	9	8	-81	
	**ACTL6A**		*BAF53A*	86	43.2	8	40	-76.9	
	**SMARCE1**		*BAF57*	6605	46.6	8	32	-101.9	
	**SMARCA1**		*SNF2L*	6594	121.1	6	8	-45.8	
	**SMARCD2**		*BAF60B*	6603	58.9	6	23	-54.6	
	**SMARCA2**		*Brm*	6595	181.2	5	7	-128.4	
	**SMARCB1**		*BAF47*	6598	44.1	4	14	-42.2	
	**ARID1A**		*BAF250*	8289	218.2	3	4	-18.2	
	**CHD7**		*KAL5*	55636	335.7	2	1	-11.1	
	**BPTF**		*NURF301*	2186	324.9	2	1	-10.7	
	**BRD1**		*BRPF1*	23774	119.4	2	4	-19.2	
	**CBX2**		*CDCA6*	84733	56	2	7	-12.2	
	**CHD5**		*CHD5*	26038	222.9	2	2	-43	
	**ARID1B**		*BAF250B*	57492	237.5	1	1	-1.6	
	**MSL3**		*MSL3*	10943	58.2	1	2	-1.7	
	**POLE3**		*CHRAC17*	54107	16.8	1	9	-1.6	
	**SMARCA5**		*SNF2H*	8467	121.8	1	2	-27.6	
**Histone PTMs regulators**	Ac	**HDAC2**	*RPD3*	3066	55.3	6	20	-51.8	
	Me	**WBP7**	*MLL4*	9757	293.3	5	4	-35.3	
	Ac	**KAT6B**	*MYST4*	23522	199.7	4	7	-29.4	
	Me	**EHMT1**	*GLP1*	79813	86.6	4	8	-27.3	
	Ph	**ZMYND8**	*RACK7*	23613	128.3	3	3	-23.9	
	Ac	**BRD8**	*SMAP*	10902	94.2	3	6	-21.2	
	Me	**MLL3**	*HALR*	58508	541	3	1	-18.8	
	Me	**MLL2**	*KMT2D*	8085	593	3	1	-18.7	
	Ac	**HDAC1**	*SMAP*	10902	55.1	2	8	-38.8	
	Me	**SETD1A**	*KMT2F*	9739	185.9	2	2	-14	
	Ub	**SHPRH**	*SHPRH*	257218	193	2	2	-11.6	
	Ac	**MORF4L1**	*MRG15*	10933	41.4	2	11	-13.7	
	Me	**EHMT2**	*G9A*	10919	128.9	2	3	-13	
	Ph	**BAZ1B**	*WSTF*	9031	170.8	2	1	-10.9	
	Me	**DOT1L**	*KMT4*	84444	164.8	1	1	-12.2	
	Ac	**CDYL**	*CDYL1*	9425	60.6	1	3	-2.3	
**Transcriptional regulators**	**SNW1**		*SKIP*	22938	61.5	16	42	-161.7	
	**SAFB**		*HET*	6294	102.7	6	17	-84.4	
	**NCOR2**		*SMRT*	9612	272.7	5	4	-39.9	
	**SIN3A**		*SIN3A*	25942	145.1	4	4	-38.6	
	**GATAD2A**		*GATAD2A*	54815	68	4	9	-34.1	
	**NCOR1**		*TRAC1*	9611	270	4	3	-35.7	
	**ZBTB4**		*ZNF903*	57659	105	3	12	-18.9	
	**HP1BP3**		*HP1BP3*	50809	61.2	2	6	-11	
	**DMAP1**		*EAF2*	55929	53	2	5	-12.9	
	**SIN3B**		*SIN3B*	23309	133	2	3	-10.9	
	**TAF1**		*TAFII250*	6872	212.5	2	2	-10.7	
	**RB1**		*OSRC*	5925	106.1	2	4	-10.4	
	**COBRA1**		*NELFB*	25920	65.7	1	3	-3.6	
	**CBX3**		*HP1γ*	11335	20.8	1	9	-1.9	
	**ZBTB33**		*ZNF-kaiso*	10009	74.4	1	2	-1.6	
	**TRIM28**		*KAP1*	10155	88.5	1	3	-2	
**Nucleosome components**	**HIST1H4L**		*H4*	8368	11.4	2	33	-21.5	
	**HIST3H2BB**		*H2B*	128312	13.9	2	25	-12	
	**HIST1H1T**		*H1*	3010	22	1	14	-1.8	
	**H2AFY2**		*H2A2*	55506	40	1	4	-2.1	
	**HIST1H2BA**		*TSH2B*	255626	14.2	1	27	-1.9	
**Others**	**MTA2**		*MTA1L1*	9219	75	15	31	-174.3	
	**ACIN1**		*ACINUS*	22985	150.5	8	8	-69.8	
	**RBBP4**		*NURF55*	5928	47.6	6	24	-59.7	
	**SMC1A**		*SMC1*	8243	143.1	6	8	-46.7	
	**MBD3**		*MBD3*	53615	32.8	4	17	-29	
	**PARP4**		*PH5P*	143	192.5	3	3	-19	
	**NASP**		*FLB7527*	4678	78.4	3	6	-19.4	
	**C14ORF43**		*ELM2*	91748	114.9	2	3	-12.1	
	**BRCA2**		*FAD*	675	384	2	1	-11.1	
	**TOX4**		*LCP1*	9878	66.2	2	18	-12.1	
	**LIG4**		*LIG4*	3981	103.9	1	1	-1.8	
	**MBD3L2**		*MBD3L2*	125997	23	1	6	-1.9	
	**EIF2C1**		*EIF2C*	26523	97.2	1	2	-1.8	
	**PRMT8**		*HRMT1L3*	56341	45.3	1	2	-1.8	
	**PRMT5**		*HRMT1L5*	10419	71.3	1	2	-1.8	
	**HERC2**		*SHEP1*	8924	526.9	1	0	-1.8	
	**BRCA1**		*IRIS*	672	202.2	1	1	-2	
	**DICER1**		*Dicer*	23405	208.3	1	1	-2.4	
	**C6ORF130**		*C6orf13*	221443	17	1	8	-2.1	
	**EIF2C2**		*AGO2*	27161	97.1	1	2	-2.7	
	**TSPYL1**		*TSPYL*	7259	49.2	1	9	-2.4	
	**TNKS1BP1**		*TAB182*	85456	181.7	1	1	-1.9	
	**BRIP1**		*BACH1*	83990	140.8	1	1	-10.8	

**Fig 2:**
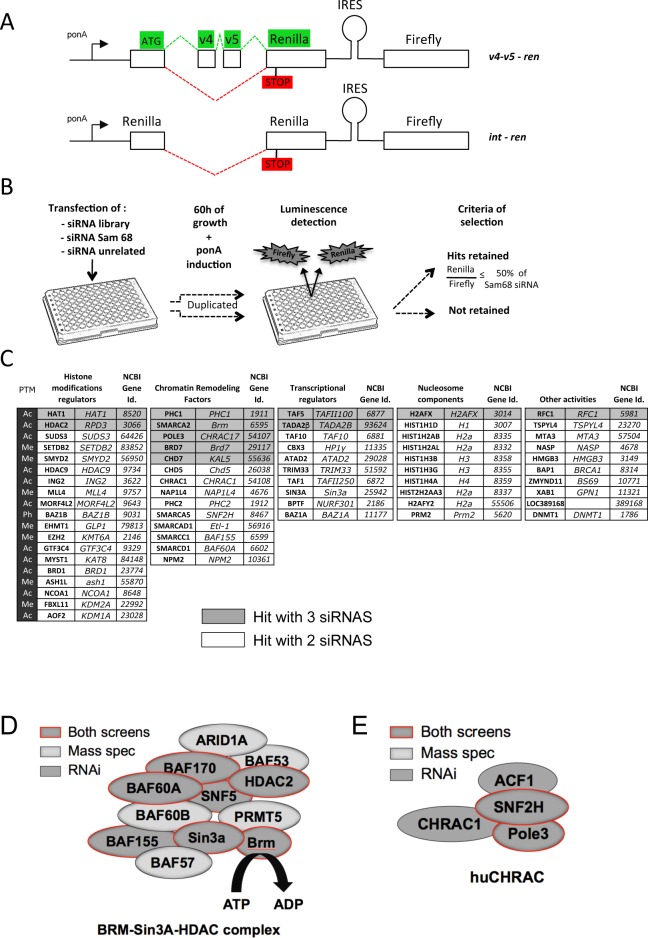
A high-throughput siRNA screen for chromatin factors that affect splicing. (A) Diagram of the bi-cistronic *v4-v5–ren* and *int-ren* minigene reporters used to follow splicing by luminescence. Rectangles represent exons, stem-loop structures represent Internal Ribosome Entry Sites (IRES). Splicing regulation leading to a functional Renilla is indicated in Green, while those producing to a non-functional Renilla are marked in Red (B) Outline of the procedure used to screen a library of siRNAs targeting 375 chromatin factors. Each gene was targeted with an average of 3 individual siRNAs, tested in duplicates. (C) List of chromatin factors modulating splicing of the v4-v5-ren reporter in 293 EcR cells. The 63 hits of the siRNA screen are grouped in five categories according to the “String” database (string-db.org). Associated post-translational modifications (PTMs) are indicated to the left of the gene names: Acetylation (Ac), Methylation (Me), Phosphorylation (Ph). Each gene is designated by its name and its NCBI identification number (NCBI Gene Id.). (D and E) Schematic display of BRM-sin3A-HDAC and huCHRAC complexes highlighting the subunits identified by proteomic or by the siRNA screen.

List of chromatin factors associated with spliceosome complexes immunopurified from gel-filtration fractions through the tagged U2 snRNP. Mass spectrometry was carried out like in [42]. Proteins are grouped in five pools as in Fig 2C; black boxes designate factors also identified in the siRNA screen. For each gene, the table provides the name, frequently used alternative names, the NCBI identification gene number (NCBI Gene Id.), the molecular weight (Mr), the number of unique peptides, the percentage of protein coverage, and a measure of the statistical confidence (Log(e)). In the pool of histone PTM regulators, the modifications are reported as follows: acetylation (Ac), methylation (Me), phosphorylation (Ph), ubiquitination (Ub). Data shown was compiled from two independent experiments.

Importantly, endogenous/tagged U2-B” and two chromatin factors (CHD4 and SMARCC1) co-sedimented with both the H complex and the spliceosome (S1D Fig). The presence of U2 snRNP subunits, splicing factors, and chromatin factors in both the H complex- and the spliceosome-fractions was confirmed by western blotting (Figs 1D, lanes 2 & 3 and S1E, lanes 1 & 2). Yet, splicing and chromatin factors were efficiently co-immunoprecipitated with FV_5_-U2-B” only when using the spliceosome-fractions (Figs 1D, lanes 4 & 5 and S1E, lanes 3 & 4). This indicated that these factors were physically together with the U2 snRNP only in the context of an assembled spliceosome. The Polycomb group protein PHC1 that was present in both the H complex- and spliceosome-fractions but not detected in the mass spectrometry data, was not co-immunoprecipitated with FV_5_-U2-B” and is shown as a negative control (S1E).

As will be further discussed below, the chromatin factors associated with FV_5_-U2-B” were enriched in subunits of chromatin remodeling complexes such as SWI/SNF, but included also readers and writers of histone modifications, and rather surprisingly histones.

Altogether, these data showed that U2-snRNPs incorporated into splicing complexes assembled onto pre-mRNAs during or immediately after their transcription, are physically associated with chromatin factors.

### Identification of chromatin factors active in the regulation of splicing

We next questioned whether the chromatin factors associated with U2-snRNPs were representative of the population of chromatin factors affecting pre-mRNA splicing. To reach a global insight on chromatin factors relevant for the regulation of splicing, we carried out an unbiased RNAi screen of virtually every known human chromatin factor, using a CD44-based splicing reporter. This ponasterone-inducible reporter translates transcriptional activity into Firefly-luciferase luminescence, while inclusion of CD44 alternative exons v4-v5 allows for in-frame splicing of the Renilla mRNA and thereby produces Renilla-luciferase activity (Fig 2A). This reporter (*v4-v5—ren*) and a variant missing the v4-v5 genomic sequence (*int—ren*–no in-frame splicing of Renilla) were inserted randomly into the genome of 293 EcR cells, a cell line not expressing the endogenous CD44 gene (S2A Fig).

Comparative analysis of two clonal cell lines having integrated either *v4-v5—ren* or the control *int—ren* construct confirmed that Renilla enzymatic activity was detected only when v4-v5 exons were present in the reporter mRNA (S2A and S2B Fig, light-grey bars). Furthermore, depletion of Sam68, a regulator of CD44 splicing [16], led to a decrease in splicing of v4-v5, which correlated with a decrease in the Renilla over Firefly luminescence ratio (referred to as the R/F ratio—S2C Fig). Thus, splicing of our reporter was regulated in a manner comparable to that expected for exons v4-v5 in the context of the endogenous CD44 gene. At note, our reporter is not insensitive to eventual changes in translation efficiencies.

We then challenged the reporter with a library of 1155 siRNAs targeting 375 different genes predicted or known to encode regulators or components of chromatin (an average of 3 siRNAs per target—Fig 2B and S2 Table). To take experimental variations into account, we compared the R/F ratio obtained with each siRNA to that obtained with control siRNAs (unrelated and Sam68, as negative and positive controls, respectively). Thus, a siRNA was considered a hit when its transfection resulted in a variation of the R/F ratio equal to at least 50% of that observed with Sam68 siRNA. A gene was selected when at least two siRNAs qualified as hits.

Using this criterion, we identified 62 chromatin factors potentially influencing splicing, including 11 factors for which the R/F ratio was affected by all three siRNAs (triple hits—Fig 2C grey case). Among the 62 factors, ING2 and MLL4 were previously identified in another siRNA screen for chromatin factors affecting splicing of a reporter construct (S3 Table) [17]. The 11 “triple hits” were retested in the conditions of the screen, and we confirmed by RT-qPCR that they all had an impact on splicing of v4-v5 exons into the reporter mRNA (S2D and S2E Fig).

Comparing the outcome of the proteomic analysis of the FV_5_-U2-B”-associated complexes and that of the siRNA screen revealed an overlap essentially limited to histones and chromatin remodeling factors (Table 1, lines with black squares). In particular, subunits of the BAF complex and its variant Brm-Sin3a-HDAC were present in both screens (Fig 2D). SNF2H and its partner Pole3 in the human Chrac complex were also identified in both screens (Fig 2E), as were several members of the CHD family. In contrast, histone modifying enzymes and transcriptional regulators identified in the siRNA screen were predominantly absent from the MS data.

### Chromatin factors regulating splicing are not all associated with U2

We next wished to assess the actual impact of the identified chromatin factors on splicing of endogenous genes. These validation experiments were carried out on not previously characterized “triple hits” from the siRNA screen and five additional “double hits” selected for their association with chromatin remodeling complexes (CHD5, SMARCAD1, SIN3a, SMARCA5, and POLE3). Among these 14 factors, 7 were found associated with the U2-snRNP (CHD7, POLE3, SIN3A, HDAC2, CHD5, SMARCA5, BRD7), while the remaining 7 were not (TAF5, H2AFx, TADA2beta, HAT1, PHC1, RCF1, and SMARCAD1).

**Fig 3:**
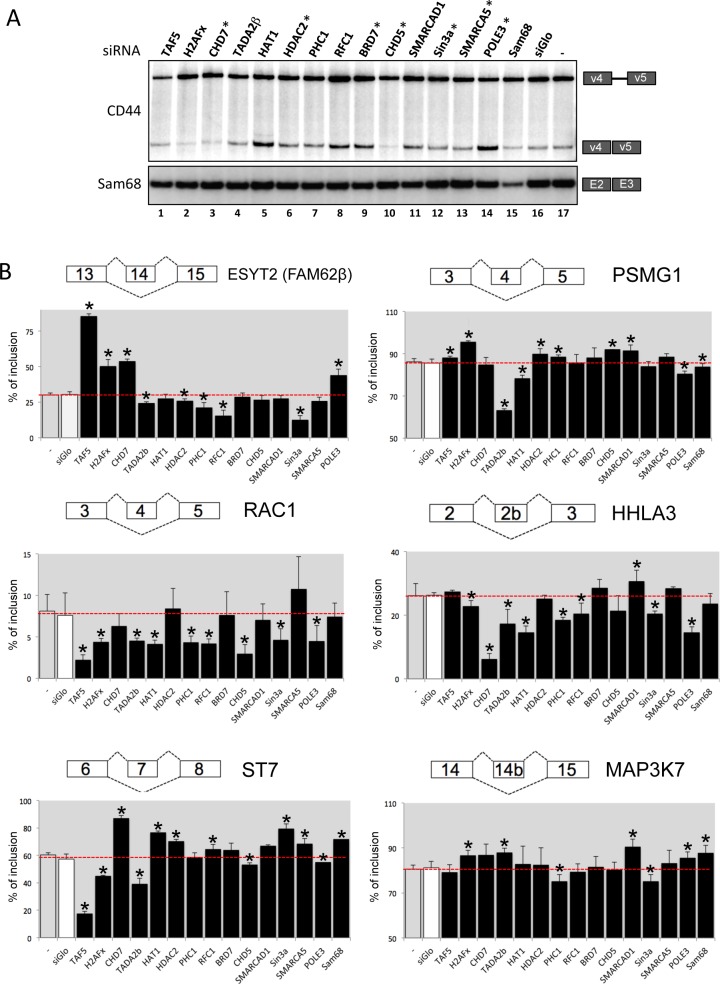
Chromatin factors affect the regulation of endogenous splicing. SiRNAs targeting 14 chromatin factors and identified as affecting splicing of v4-v5-ren in 293-ECR cells were transfected into HeLa cells as well as siRNA targeting controls. (A) Radiolabeled RT-PCR was then used to examine splicing of exons v4-v5 from endogenous CD44 and exons 2 and 3 from Sam68. The chromatin factors identified by mass spectrometry are highlighted with asterisks. (B) Graphs displaying the percentage of inclusion for six alternative exons previously described as sensitive to U2 snRNP activity [18]. Exon inclusion was detected by radiolabeled RT-PCR (S3D–S3F Fig) using total RNA of HeLa cells depleted for the indicated chromatin factors and controls. Graphs display effects obtained from triplicate experiments; asterisks indicate p-val<0.05. The stippled line shows the percentage of inclusion for untreated and siGlo-transfected cells, while the exons amplified by RT-PCR are drawn on the top of each graph.

Depletion of 8 of these factors with siRNAs resulted in reduced usage of the endogenous CD44 variant exons v4-v5 in HeLa cells, a cell line naturally expressing CD44 (Figs 3A, S3A and S3B). In contrast, depletion of CBX8, HDAC3, and NCAPD2, three chromatin factors identified neither in the U2 snRNP proteomic study nor in the siRNA screen, did not affect usage of the v4-v5 exons (S3C Fig).

We next examined the effect of the depletion of the 14 selected chromatin factors on several exons previously described as being particularly sensitive to U2 snRNP activity [18], reasoning that factors associated with the U2 snRNP were likely to affect the inclusion of these exons (Figs 3B, S3D and S3E). In this series of experiments, depletion of 13 chromatin factors, present or not in the FV_5_-U2-B”-associated complex, affected inclusion of multiple alternative exons. Only Brd7 was found not to affect any exon. The 13 chromatin factors active on the U2 snRNP-sensitive exons had no effect on constitutive exons in the RAC1 or the PSMG1 genes, indicating that overall splicing activity was not affected (S3F Fig); neither did their depletion affect expression of hnRNPU, an hnRNP that influence the maturation and activity of the U2 snRNP [18]. We also observed no correlation between the effect of the 13 chromatin factors on splicing and that on expression of several essential splicing regulators, some of which had putative binding sites in the exons we examined (SRSF1, SRSF3, SRSF4, SRSF5, SRSF6, and hnRNPA1, S3G Fig).

These experiments showed that an unexpectedly wide range of chromatin factors can impact on the outcome of splicing, although some were not identified by proteomic as physically interacting with the U2 snRNP in spliceosome complexes. Yet, sensitivity of an exon to levels of U2 appears as a good predictor of its sensitivity to chromatin, suggesting that U2 is responsive to chromatin-born information. Finally, we note that depletion of the various chromatin factors resulted in either increased or decreased inclusion depending on the exon under scrutiny, revealing that the impact of chromatin factors varies from one exon to the other, possibly as a function of local levels of chromatin compaction or histone modifications.

### Chromatin modulates splicing efficiency

**Fig 4:**
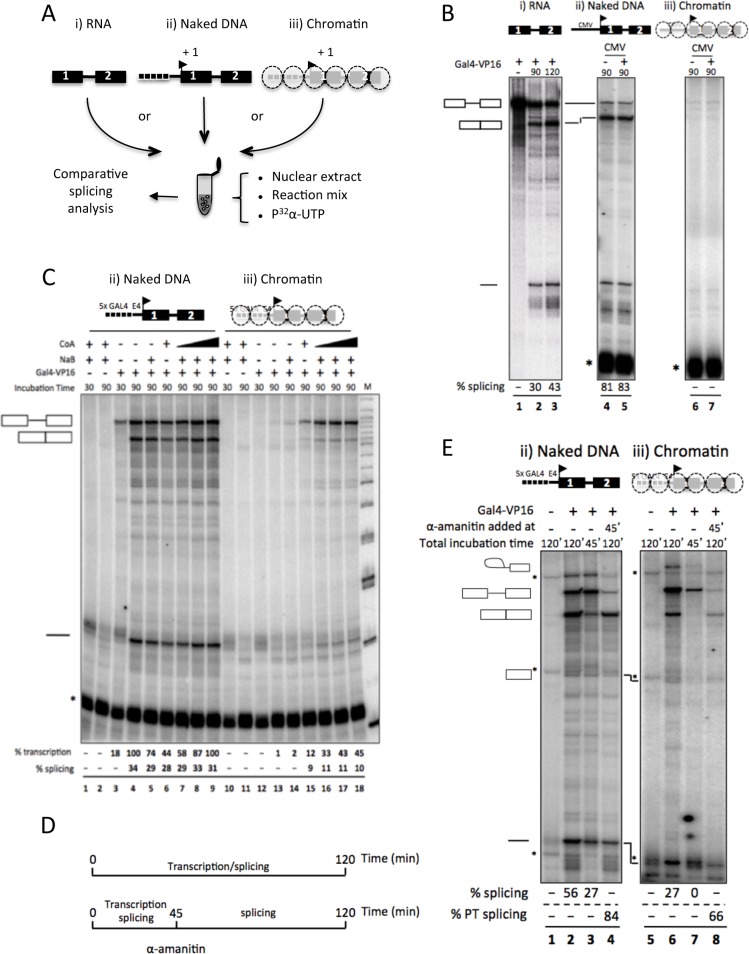
Chromatin affects the efficiency of intron removal *in vitro*. (A) Diagram of the three different sources of Ftz reporter RNA used to study *in vitro* the impact of chromatin on splicing. (i) A capped pre-mRNA that was independently transcribed with the T7 RNA polymerase before being added to HeLa nuclear extract; (ii) pre-mRNA was transcribed in HeLa nuclear extract from a naked, or (iii) a chromatinized DNA template. Regardless of the source, the pre-mRNAs are identical, with two exons and one intron originating from the *Drosophila Ftz* pre-mRNA. (B) Analysis of the products of *in vitro* splicing of the pre-synthesized RNA reporter (lanes 1–3), of a transcription/splicing reaction transcribing the RNA reporter from a naked DNA template (lanes 4–5), or from a chromatinized template (lanes 6–7). Transcription/splicing was assayed in the absence (-) or in the presence (+) of Gal4-VP16. For each condition, the RNA was resolved on a 6% denaturing polyacrylamide gel; the relative abundance of spliced mRNA indicated at the bottom of each lane was calculated as follows: % splicing = [spliced/(unspliced+spliced)]. The asterisk indicates the labeling of U6 snRNA by a terminal uridylyl transferase present in HeLa nuclear extract [15,46]. (C) The influence of NaB and CoA on *in vitro* transcription/splicing was evaluated in reactions assembled with naked or chromatinized DNA template. The presence (+) or absence (-) of Gal4-VP16, NaB, CoA and the time of incubation are indicated above the gel. The transcription level was calculated as the sum of unspliced and spliced RNA, and lane 4 was arbitrarily set at 100%. The splicing efficiency was calculated as in Fig 4B. (D) The experimental procedure applied in Fig 4E is displayed with a chronogram. (E) Chromatin affects the quality of pre-mRNP released by RNAPII transcription. The transcription/splicing reactions of naked or nucleosomal template were performed for 45 min (lanes 3 and 7) or 120 min (lanes 1, 2, 4, 5, 6, 8). α-amanitin was added to the reactions after 45 min of incubation (lanes 4 and 8) and the reactions were extended for additional 75 min. The presence (+) or absence (-) of Gal4-VP16 is indicated. The percentage of splicing (% splicing) was calculated as in (B) for lanes 2 and 6; while the percentage of post-transcriptional splicing in lanes 4 and 8 was calculated as followed: % PT splicing = [(spliced_120_-spliced_45_)/(unspliced+(spliced_120_-spliced_45_))].

To gain information on how chromatin may influence splicing, we next developed an *in vitro* assay (see Methods and Fig 4A), which allowed a side-by-side comparison of splicing performed by (i) the spliceosome alone, (ii) splicing coupled to transcription, and (iii) splicing coupled to both transcription and chromatin decondensation.

We chose a reporter construct based on *Drosophila* Fushi tarazu (Ftz) exon 1 and 2, previously used in an *in vitro* system coupling RNAPII transcription to spliceosome assembly [19]. This splice reporter was put under the control of a CMV promoter, or a chimeric promoter with five GAL4 binding sites located upstream of a minimal Adenovirus E4 promoter (Gal4-E4—S4A Fig). For the latter, transcriptional activity was achieved by supplementing all *in vitro* reactions with the chimeric transcriptional activator Gal4-VP16.

Co-transcriptional splicing of the pre-mRNA synthesized from the CMV-Ftz DNA template was more efficient than splicing of an identical pre-synthesized and capped pre-mRNA, consistent with earlier observations ([19] and Fig 4B, compare lanes 2 and 5). This difference in splicing efficiency was less discernible when the Gal4-E4-Ftz DNA template was used instead (compare Fig 4B lane 2 and Fig 4C lane 4), possibly reflecting previously described influence of promoter sequences on splicing [20,21].

To then evaluate the impact of nucleosomes on our co-transcriptional splicing assay, the DNA template was chromatinized by combining purified recombinant human chromatin assembly complex ACF (SMARCA5 and BAZ1A) and histone chaperone NAP-1 (NAP1L1) with purified HeLa core histones in the presence of ATP [22]. The regularity of nucleosome spacing on the DNA template was confirmed by micrococcal nuclease digestion, which revealed protected DNA fragments corresponding to a ladder of mono-, di- and oligo-nucleosomes, mimicking the nucleosome periodicity observed with native chromatin (S4B Fig).

Chromatinization of the CMV-Ftz DNA template strongly reduced transcription, making assertion of the splicing efficiency virtually impossible (Fig 4B, compare lanes 4, 5 and 6, 7). Chromatinization also reduced transcription from the Gal4-E4-Ftz DNA template, although less radically, and transcriptional activity was partially recovered (approx. 50% of that observed on naked DNA) by supplementing the *in vitro* reactions with acetyl coenzyme A (CoA), and sodium butyrate (NaB) (Fig 4C, compare lanes 9 and 18). Acetyl CoA, a co-factor of histone acetylases, and sodium butyrate, an inhibitor of histone deacetylases, favor histone acetylation and thereby participate in licensing the chromatin for transcription. Neither Acetyl CoA, nor sodium butyrate, nor Gal4-VP16 affected splicing of the pre-synthesized pre-mRNA (S4C Fig, lanes 2–6). We also verified that the ratio between extract and either naked or chromatinized template had no effect on splicing. These experiments indicated that levels of transcription did not affect the efficiency of the splicing reaction (% of splicing), and also that increased concentration of chromatin constituents did not have any inhibitory effect on splicing (S4D Fig). From these validation experiments, we concluded that our conditions properly emulated chromatin-decondensation associated with co-transcriptional splicing.

Interestingly, our *in vitro* assay showed that the transcription of a chromatinized template leads to pre-mRNA splicing that is less efficient than that detected using a naked DNA template (Fig 4C, compare lanes 7–9 and 16–18, with 30% *vs*. 10% splicing efficiency). This observation is the first evidence for a direct effect of chromatin on splicing efficiency.

To gain insight in the mechanism behind this impact of chromatin on splicing efficiency, we investigated whether the effect was co- or post-transcriptional. To that end, *in vitro* reactions with the Gal4-E4-Ftz minigene were supplemented with α-amanitin after 45 min of transcription and either stopped (ice) or incubated for another 75 min at 30°c (chronogram Fig 4D). The α-amanitin blocks RNAPII processivity without directly affecting splicing (S4C Fig, compare lanes 4 and 6). As expected from the previous experiments, splicing during the first 45 min (phase of transcription and splicing) was less efficient when using the chromatinized template (undetectable *vs*. 27%—Fig 4E, compare lanes 3 and 7). Interestingly, decreased efficiency of splicing as a consequence of chromatin was also observed post-transcriptionally after the addition of α-amanitin (phase of just splicing—Fig 4E, compare lanes 4 and 8). Chromatin-dependent reduction in post-transcriptional splicing efficiency was also observed when using two additional reporters where Ftz exons 1 and 2 were separated by exons harboring (S) or not (T) 3 copies of an SF2-binding sites (S4E Fig, compare lanes 2 and 6, and 8 and 12). These constructs where the S sequences lead to full inclusion of the intervening exon, while the T sequences results in its exclusion, were also an opportunity to observe that chromatin is unable to override a decision enforced by SR proteins (S4E Fig, species a and c). Altogether, these experiments indicate that the pre-mRNPs generated from the naked and chromatinized templates were not equally competent for splicing. This strongly suggests that chromatin influences the quality of pre-mRNPs assembled co-transcriptionally, which in turn affects the efficiency of splicing. Yet, our observations also suggest that chromatin is involved only in fine-tuning of splicing, with little impact on the effect of splicing enhancers.

## Discussion

Co-transcriptional removal of introns occurs in the vicinity of other gene expression machineries, including the RNAPII and the chromatin remodeling factors. While the impact of the RNAPII is now well documented, a role for chromatin in the regulation of splicing is sustained mostly by correlative observations, and the mechanisms involved remain unclear. Here, we have provided a comprehensive study of the coupling between chromatin and splicing, and we have established an *in vitro* system to examine this coupling directly. Although we have at this point examined only a limited number of reporter constructs, our data indicate that transcribing pre-mRNA from a chromatinized template influences splicing efficiency, and we propose that this effect is in part mediated by physical interactions between chromatin factors and the spliceosome.

Our RNAi screen identified a surprisingly broad range of factors, rather than a specific subset of chromatin complexes. The screen caught nearly every chromatin factor previously reported to modulate splicing (SWI/SNF, Cbx3/HP1γ, ZMYND11/BS69, CHDs…), supporting the relevance of the hits. Some of these factors, including Cbx3/HP1γ, and ZMYND11/BS69 have been examined for their genome wide effect on splicing, further suggesting that our hits affect exons beyond those examined during the phase of validation [4,23]. These genome-wide studies and others on MBD3 and CHD4 also indicate that these chromatin factors only have minor effects on the expression of splicing factors, including SRSF1, SRSF3, SRSF4, SRSF5, SRSF6, and hnRNPA1 [24,25].

A reasonable explanation for the diversity of the hits is the presumed heterogeneity of the local levels of chromatin compaction and/or the range of histone modifications surrounding each copy of our integrated splicing reporter, like it has for example been described for the various copies of endogenous histone genes (The Encode Project Consortium). In that sense, our screen may serendipitously have probed a large spectrum of chromatin environments influencing the regulation of splicing. The local influence of chromatin was also illustrated by our validation experiments on endogenous genes. These experiments showed that depending on the exon under scrutiny, a given chromatin factor had a variable effect, favoring either exon inclusion or exclusion in a rather unpredictable manner. This is in agreement with an earlier study showing that in human breast cancer MCF7 cells, the HDAC inhibitor TSA and the DNA methylase inhibitor 5azadC promote the inclusion exon E107 of the SYNE gene, while they induce exclusion of exon E33 of the fibronectin gene [26]. Likewise, in Drosophila S2 cells, depletion of SWI/SNF subunits promotes the use of proximal splice sites at some genes, while it favors distal sites at others [27]. A possible source of heterogeneity in the chromatin of exons may be their degree of proximity with promoters and enhancers, caused by DNA looping [28]. The deciphering of the probably very complex combination of regulatory signals at play at a given locus will be required to meet the challenge of anticipating the per gene impact of a chromatin factor on splicing.

Our proteomic approach confirmed that the splicing machinery is physically bound to a subset of chromatin factors when spliceosome complexes are assembled *in vivo*. Some of these factors were previously connected to splicing, including MORF4L2 (close homolog MRG15), Cbx3/HP1γ, SMARCA2/BRM, EHMT1 and EHMT2, EZH2, and multiple HDACs [2–4,29–31]. In several earlier proteomic studies of the splicing machinery, such interactions were not detected, or were limited to a few factors. This is likely rooted in the procedures used for purification as these approaches involved characterization of the splicing machinery assembled *de-novo* on pre-synthetized reporter RNAs. With such a setup, components normally dispensed during transcription will not be loaded onto the spliceosome. Our procedure based on U2-snRNP anchoring overcomes this limitation and allows for the isolation of both *de-novo-* and *in-vivo*-assembled spliceosome complexes. In that sense, it resembles the previously described capture of the PRPF40A-U2 snRNP that revealed the presence of CHD4/8 and several SWI/SNF subunits in addition to splicing factors [8]. Among the 15 remodeling factors present in that complex, 13 were also detected by our approach.

The U2 snRNP is one of the best-characterized snRNPs of the spliceosome, and while several versions have been described, corresponding to different maturation stages [32], it is likely that only the most abundant particles have been characterized so far, excluding those associated with the transcribed chromatin. Historically, both genetic and biochemical studies have considered the snRNPs as essential rather than regulatory components of the spliceosome. Recent studies, however, demonstrated that several alternative splicing events are regulated by the levels of core components of the splicing machinery [18,33]. The exons we examined to validate our hits were identified as particularly sensitive to levels of U2-snRNP. We speculate that this snRNP may function as a mediator between the splicing machinery and the local chromatin environment, and that exons sensitive to U2-snRNP activity are also likely to be subject to chromatin effects.

Finally, we note that the list of “chromatin factors” physically linked to the spliceosome in our proteomic approach actually included histones. This suggests that these primary building blocks of chromatin may impact on the outcome of splicing, possibly by affecting nucleosome assembly when present in limited supply. Indeed, nucleosomes may be involved in exon definition as suggested by the elevated nucleosome occupancy/positioning observed in exons compared to introns (for a review, see [34]). Nucleosome assembly may also be relevant for RNAPII elongation rate and for the formation of loops connecting alternative exons to promoter-positioned nucleosomes [28,35]. In this context, we believe that our *in vitro* system combining chromatin, transcription, and splicing will provide a powerful tool to unravel the molecular network linking histones to spliceosome components during the course of transcription.

## Materials and Methods

### RNAPII transcription and splicing assay

DNA templates containing promoter and reporter were generated by PCR, purified and 40 ng of DNA were added to a 15-μl *in vitro* transcription/splicing reactions. Assays were performed by mixing 5-μl of HeLa nuclear extract (NE) prepared as described [19], 5-μl of transcription/splicing mix and 5-μl template, then incubation at 30°C. Transcription/splicing mix was assembled for each reaction with 0.20 μl ^32^P-UTP (3000 Ci/mmol), 0.5 μl 25× ATP/CP mix (12.5 mM ATP, 0.5 mM creatine phosphate (di-Tris salt)), 0.5 μl MgCl_2_ (80 mM), 0.75 μl Hepes-KOH (0.4 M), 0.1 μl dNTP (1 mM), 0.5 μl 25× NTP mix (0.2 mM UTP, 0.6 mM GTP, 3.75 mM CTP and ATP), 0.05 μl sodium butyrate (400 mM), 0.05 μl acetyl coenzyme A (1 mM), and H_2_0 up to 5-μl. To activate transcription of template containing GAL4 promoter, the NE was supplemented with 20 ng of recombinant Gal4-VP16, while to inhibit transcription 200ng of α-amanitin was added per reaction. The dNTP/NTP mix is not required, but in our hands, it significantly increased the efficiency of transcription and removed some unspecific bands associated with DNA synthesis. *In vitro* splicing of pre-mRNA templates was carried out like for transcription/splicing assays.

### Plasmid constructions

To construct the Ftz splicing reporter, a fragment containing exon 1 (256 bp), intron 1 (147 bp), and exon 2 (186 bp) was amplified by PCR from the DoF1 plasmid [19], and inserted HindIII/XbaI in pcDNA3.1(+) downstream of a CMV promoter. The GAL4-E4-Ftz constructs were generated by replacement of the CMV promoter between the MluI and HindIII restriction sites. To generate the derivative constructs with exon T or S, a *Cla*I restriction site was created in the intron of constructs mentioned above and PCR fragments containing the respective DUP exons [36,37] bordered by a small part of the intron were cloned into the *Cla*I restriction site. The DNA templates for transcription/splicing assay were amplified by PCR using the universal primers CTTAGGGTTAGGCGTTTTGCGCTG and CAACTAGAAGGCACAGTCGAGGCTG.

The luciferase splicing reporters v4-v5–ren and int-ren were inserted into the ecdysone-inducible vector pI-TK Hygro (kindly provided by R. Karni, Hebrew University Medical School–Jerusalem, Israel) between the restriction sites HindIII and XhoI (ligated cohesively with SalI). Cloning of these reporter constructs required multiple steps; in brief, the first two ATG codons of Renilla cDNA were removed, an exogenous intron with or without the v4-v5 genomic part of the human CD44 gene was inserted, and finally an IRES and the Firefly luciferase cDNA were inserted downstream of a Renilla cDNA. The details of each construct are available upon request. The pBabe-FV5-U2-B” construct was generated by inserting a U2-B” cDNA into the pBabe vector downstream of Flag and V5 tags [38].

### Chromatin assembly and MNase footprint

DNA template were chromatinized as described in [39] using the Chromatin Assembly Kit (Active Motif). The chromatinized DNA was digested with MNase as described in [39] for 0, 30, 75 and 150 sec and analyzed by agarose gel electrophoresis/ethidium-bromide staining.

### Mass spectrometry and antibodies

The HeLa S3 cell line expressing FV5-U2-B” was generated by viral infection with pBabe-FV5-U2-B” and a clonal cell line stably expressing the tagged protein at a high level was selected by immunofluorescence using anti-V5 antibody (Invitrogen) and expanded to prepare nuclear extract. Endogenous PP2Cγ hnRNPA1 and U2-B” levels were estimated by western blotting with monoclonal antibodies 7–53, 4B10 and 4G3, respectively [38]. U2 snRNP was immunopurified from fresh NEB” nuclear extract using ANTI-FLAG M2 Affinity Gel, then eluted with the 3× FLAG Peptide (Sigma-Aldrich), and resolved on a 15–35% glycerol gradient as described [40]; the proteins and RNAs were analyzed on SDS-PAGE and urea gels respectively. Immunoprecipitations from in vitro splicing reactions were performed as above, but the enriched RNAs were isolated directly from the beads without elution. To enrich spliceosome complexes, in-vitro-splicing reactions (1 ml) were set up in the presence of ATPγS [15] and by using the AdML pre-mRNA reporter transcribed from the DoA1 plasmid [19]. The reactions were next size-selected on a Sephacryl-S500 gel-filtration column (approx. 10^6^ to 5.10^6^ Da) to resolve complexes assembled onto the radiolabelled splicing reporter [41]. The pooled fractions corresponding to each complex were incubated with anti-FLAG M2 Affinity Gel and the immunopurified factors were analyzed by mass spectrometry as described [42]. Western-blot analysis was carried out with the following antibodies: anti-SF3a120, 66, 60 (gift from A. Krämer, Department of Cell Biology, University of Geneva, Switzerland); anti-U2AF65 [42]; anti-U2-B” (mAb 4G3); anti-eF4A3 [43]; anti-hnRNPC1/C2 (mab 4F4); anti-hnRNPA1 (mAb A1/55); anti-CBX3 (mAb 1G6); anti-CHD4 (Sigma, WH0001108M1); anti-SMARCC1 (Sigma, B5186); anti-SMARCA4 (mab1E1); anti-SMARCA2 (mab4147); anti-PHC1 (Sigma, HPA006973).

### RNA purification and RT-PCR

The RNA samples were prepared as described [44] and the cDNA libraries were synthesized with M-MLV reverse transcriptase using oligo dT priming or random primers. Radioactive PCRs were performed using 5’end-labeled primers. The primers used in this study are available upon request.

### siRNA screen of chromatin factors and cell culture

The siRNA library was acquired from Qiagen (each siRNA is listed in S2 Table). The Human Sam68 siRNA (CGGATATGATGGATGATAT;[45] and siGlo (Dharmacon) were used as controls. 293EcR and HeLa cells were transfected with Lipofectamine RNAiMAX (Invitrogen). Regarding the siRNA screen conditions, 104 293EcR v4-v5-ren cells were transfected with the siRNA (25 nM final concentration) in 96-well plates. 60h after transfection, expression of the reporter was induced with Ponasterone A (1 μM final concentration) for 16 h and then the cells were lysed in the following buffer (100 mM Tris (pH 8), 0.5% v/v Nonidet P-40, 10 mM DTT). Renilla and Firefly luciferase activities were measured with a Dual-Glo Luciferase assay system (Promega) using a FLUOstar OPTIMA microplate reader (BMG Labtech).

### Recombinant protein purification

Recombinant Gal4-VP16 was produced in bacteria, and then first enriched using a histidine tag, followed by ion-exchange chromatography using a SP-FF Hitrap, and finally gel-filtration chromatography with a Superose 12 column.

## Supporting Information

S1 Fig Related to Fig 1: Analysis of protein and RNA components associated with the purified U2 snRNP. (A) Protein analysis of the U2 snRNP (12*S* and 17*S*) immunopurified via FV_5_-U2-B” from the NE_B”_ nuclear extract. The U2 snRNPs was resolved on a 10–30% glycerol gradient and the proteins corresponding to fractions labeled in S1B Fig were analyzed in SDS-PAGE stained by Coomassie-blue. Individual proteins were identified by comparison with earlier work [29] and their presence was confirmed by proteomic analysis of spliceosome complexes (S1 Table). (B) RNA associated with the U2 snRNP immunoprecipitated with FV_5_-U2-B”, eluted with Flag peptides and loaded on a glycerol gradient was analyzed by separation on a 7% denaturing polyacrylamide gel and stained with ethidium bromide (lanes 1–16). The input RNAs contained in NE_B”_ were also analysed to estimate the enrichment of U2 snRNA after purification of the snRNP. The two main peaks of U2 snRNA detected in the gradient correspond to the 12*S* and 17*S* forms of the U2 snRNP (lanes 3–6 and 7–10). (C) Analysis of splicing products associated with the Flag-tagged U2 snRNP incorporated into spliceosomes *de-novo*-assembled. Splicing reactions were assayed with AdML pre-mRNA in NE (lanes 1–5) or NE_B”_ (lanes 6–10) and analyzed by gel electrophoresis and autoradiography. (D) Proteins present in fractions obtained after gel filtration like in Fig 3C were precipitated and separated in SDS-PAGE to analyze them by western blot using specific antibodies detecting CHD4, SMARCC1, SRSF6 and U2-B”. (E) Western blot analysis of several chromatin factors (CHD4, SMARCC1, SMARCA4, SMARCA2 and PHC1) for their co-immunoprecipitation with U2-B” from spliceosome and H gel filtration fractions.
(PDF)

S2 Fig Related to Fig 2: Analysis of luciferase splicing reporters in 293-*EcR* cell lines. (A) Several amplicons corresponding to various portions of bicistronic transcript expressed by the two splicing reporters were amplified by radioactive RT-PCR (lanes 1–6). Expression of exons v4-v5 was only detected in the 293-EcR v4-v5-ren cells, while the Firefly luciferase transcript was specifically amplified in the two reporters cell lines (compare lanes 1–4 with 5 and 6). The amount of amplified transcripts reproduces the levels of luminescence detected for each enzyme (S2B Fig), supporting the correlation between the transcript levels and the luminescence measured. Two endogenous regulators of exons v4-v5 were also detected as controls to show the absence of a significant effect of ponA on their transcript level. (B) Relative Renilla and Firefly luminescence detected in 293-EcR cell lines, in absence (-) or presence (+) of ponasterone A (ponA) to induce reporter expression. The uninduced 293-EcR v4-v5-ren cells displayed leaky transcription but remained inducible. (C) Depletion of Sam68 decreases splicing of v4-v5 exons and reduces Renilla luminescence. An unrelated siRNA (siGlo) was transfected as control. (C, left panel) Knockdown of Sam68 specifically reduces the expression of Renilla luciferase. Splicing efficiency was calculated as the ratio between both luciferases. (C, right panel) The efficiency of v4-v5 splicing was estimated by radioactive RT-PCR and bands were quantified with a PhosphoImager (graph on top). (D and E) Validation of individual siRNAs targeting the hits of group A. Each siRNA was transfected into the clonal 293-EcR v4-v5-ren cells and its effect on luciferase activities and splicing of exogenous v4-v5-ren was evaluated by luminescence and RT-qPCR, respectively. Unspliced and spliced v4-v5 exons were both amplified in the same RT-qPCR reaction and the level of each product was obtained by measuring SYBR-green incorporation in the linear range of the PCR.
(PDF)

S3 Fig Related to Fig 3: Assessment of splicing misregulation for endogenous genes after depletion of chromatin factors in HeLa cells. (A) The efficiency of gene silencing using specific siRNA was assessed by RT-qPCR using as control samples untreated or treated with siGlo. (B) Percentage of exon v4-v5 splicing displayed in Fig 3A. The percentages and standard deviations (Stdv) were derived from three independent experiments. The red discontinuous line shows the splicing percentage of exons v4-v5 in untreated and siGlo treated cells. (C) Analysis for splicing of exon v4-v5 in HeLa cells treated with several siRNA targeting chromatin factors retained as hits (CHD7, CHD5, POLE3) or unselected in our screen (CBX8, HDAC3 and NCAPD2). The efficiency of siRNAs for each factor is displayed in the top, while the percentage of exon v4-v5 splicing is presented at the bottom. (D and E) Radiolabeled RT-PCR was used to examine the inclusion of exons reported as sensitive to U2 snRNP activity. PCR products were separated in undenaturing acrylamide gel, then dried and exposed for their detection by phosphoimager. A draw on the right of each gel displays the exons composition associated to bands. (F) Similar analysis to D and E but applied to *RAC1*, *PSMG1 and hnRNPU* constitutive exons. (G) The effect of the chromatin factors on splicing cannot be correlated with their effect on the expression of the splicing regulators SRSF1, SRSF3, SRSF4, SRSF5, SRSF6 and hnRNPA1. Left panels: putative binding sites for SRSF1, SRSF5 and SRSF6 within the indicated exons; the binding sequences and their mapping within the exons were obtained using ESEfinder 3.0 (http://rulai.cshl.edu/cgi-bin/tools/ESE3/esefinder.cgi?process=home). Right panels: levels of indicated splicing regulators was quantified by RT-qPCR after knock-down of each of the chromatin factors examined in Fig 3A. Fold-change was then calculated relative to siGlo and plotted as a function of the effect of the chromatin factor on splicing. The scatter plots show an absence of correlation between splicing regulator expression and inclusion of exons showed in samples of Fig 3.
(PDF)

S4 Fig Related to Fig 4: *In vitro* transcription-splicing system using chromatinized templates. (A) Diagram of RNA and DNA reporter templates with two exons. (B) MNase footprint analysis of an *in vitro*-assembled polynucleosomal DNA template. DNA markers, mono- and multimers of nucleosome are indicated. (C) Gal4-VP16, NaB/CoA, and α-amanitin were evaluated for their influence on the regulation of splicing, independently of their role in transcription. They were added to *in vitro* splicing reactions (lanes 3–6) with the RNA version of the reporter for 120 or 45 min as indicated. Then, the percentage of splicing was compared to the basic conditions (lanes 1, 2). (D) Various amount of naked or chromatinized templates were added in transcription-splicing reactions in order to assess potential splicing variation brought by templates. On the top of gel, the quantity of templates added in each reaction is reported, while the percentage of splicing calculated as in the Fig 4B is displayed at the bottom. All reactions were designed with the GAL4-E4-Ftz reporter. (E) Similar experiments to the Fig 4E were performed using two other reporters containing 3 exons. The reporter S integrates three copies of an ESE bound by SRSF1 in its central exon, while the reporter T inserted three sequences inactive for splicing. A precise description of S and T sequences could be found in (Labourier E et al., 1999-NAR [36]). The splicing percentage were calculated as in Fig 4E.
(PDF)

S1 Table Related to Fig 1: Mass-spectrometry data of splicing factors. Table organized as in [44].
(PDF)

S2 Table Related to Fig 2: Library of siRNAs used to screen chromatin factors. (PDF)

S3 Table Comparing the outcome of the siRNA screen and that of the proteomic of the U2 snRNP with the outcome of the siRNA screen described by Salton et al. [17]. Chromatin factors identified in both screens are highlighted in yellow; Non-overlapping siRNAs are highlighted in red or green.
(PDF)
