# Supplementary figures and images for: A Broad Set of Chromatin Factors Influences Splicing
Source: PLoS Genet. 2016 Sep 23;12(9):e1006318. doi: 10.1371/journal.pgen.1006318 (PMC5035054; doi:10.1371/journal.pgen.1006318)

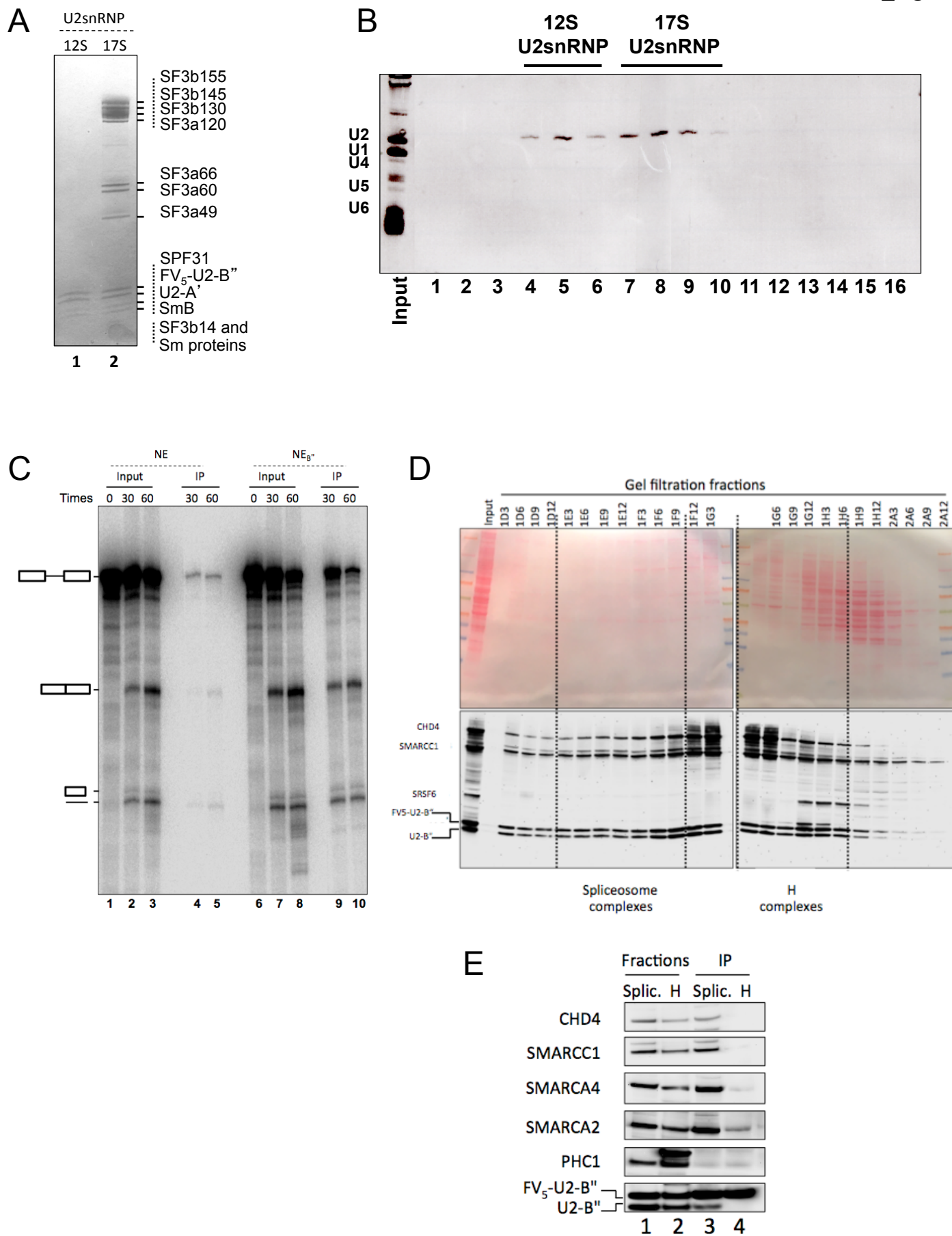

Supplement: S1 Fig — (A) Protein analysis of the U2 snRNP (12S and 17S) immunopurified via FV5-U2-B” from the NEB” nuclear extract. The U2 snRNPs was resolved on a 10–30% glycerol gradient and the proteins corresponding to fractions labeled in S1B Fig were analyzed in SDS-PAGE stained by Coomassie-blue. Individual proteins were identified by comparison with earlier work [29] and their presence was confirmed by proteomic analysis of spliceosome complexes (S1 Table). (B) RNA associated with the U2 snRNP immunoprecipitated with FV5-U2-B”, eluted with Flag peptides and loaded on a glycerol gradient was analyzed by separation on a 7% denaturing polyacrylamide gel and stained with ethidium bromide (lanes 1–16). The input RNAs contained in NEB” were also analysed to estimate the enrichment of U2 snRNA after purification of the snRNP. The two main peaks of U2 snRNA detected in the gradient correspond to the 12S and 17S forms of the U2 snRNP (lanes 3–6 and 7–10). (C) Analysis of splicing products associated with the Flag-tagged U2 snRNP incorporated into spliceosomes de-novo-assembled. Splicing reactions were assayed with AdML pre-mRNA in NE (lanes 1–5) or NEB” (lanes 6–10) and analyzed by gel electrophoresis and autoradiography. (D) Proteins present in fractions obtained after gel filtration like in Fig 3C were precipitated and separated in SDS-PAGE to analyze them by western blot using specific antibodies detecting CHD4, SMARCC1, SRSF6 and U2-B”. (E) Western blot analysis of several chromatin factors (CHD4, SMARCC1, SMARCA4, SMARCA2 and PHC1) for their co-immunoprecipitation with U2-B” from spliceosome and H gel filtration fractions. (PDF) [file pgen.1006318.s001.pdf]

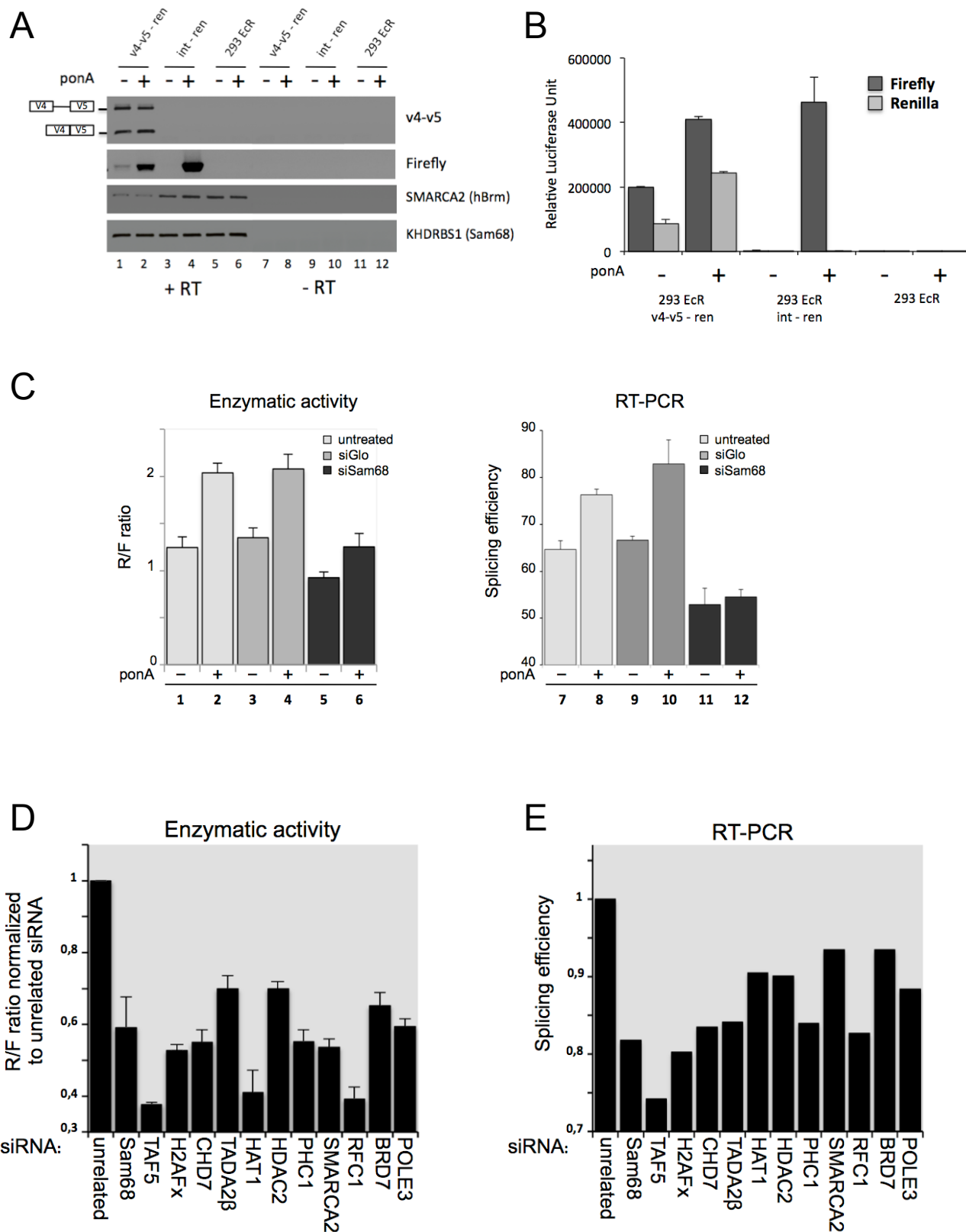

Supplement: S2 Fig — (A) Several amplicons corresponding to various portions of bicistronic transcript expressed by the two splicing reporters were amplified by radioactive RT-PCR (lanes 1–6). Expression of exons v4-v5 was only detected in the 293-EcR v4-v5-ren cells, while the Firefly luciferase transcript was specifically amplified in the two reporters cell lines (compare lanes 1–4 with 5 and 6). The amount of amplified transcripts reproduces the levels of luminescence detected for each enzyme (S2B Fig), supporting the correlation between the transcript levels and the luminescence measured. Two endogenous regulators of exons v4-v5 were also detected as controls to show the absence of a significant effect of ponA on their transcript level. (B) Relative Renilla and Firefly luminescence detected in 293-EcR cell lines, in absence (-) or presence (+) of ponasterone A (ponA) to induce reporter expression. The uninduced 293-EcR v4-v5-ren cells displayed leaky transcription but remained inducible. (C) Depletion of Sam68 decreases splicing of v4-v5 exons and reduces Renilla luminescence. An unrelated siRNA (siGlo) was transfected as control. (C, left panel) Knockdown of Sam68 specifically reduces the expression of Renilla luciferase. Splicing efficiency was calculated as the ratio between both luciferases. (C, right panel) The efficiency of v4-v5 splicing was estimated by radioactive RT-PCR and bands were quantified with a PhosphoImager (graph on top). (D and E) Validation of individual siRNAs targeting the hits of group A. Each siRNA was transfected into the clonal 293-EcR v4-v5-ren cells and its effect on luciferase activities and splicing of exogenous v4-v5-ren was evaluated by luminescence and RT-qPCR, respectively. Unspliced and spliced v4-v5 exons were both amplified in the same RT-qPCR reaction and the level of each product was obtained by measuring SYBR-green incorporation in the linear range of the PCR. (PDF) [file pgen.1006318.s002.pdf]

A

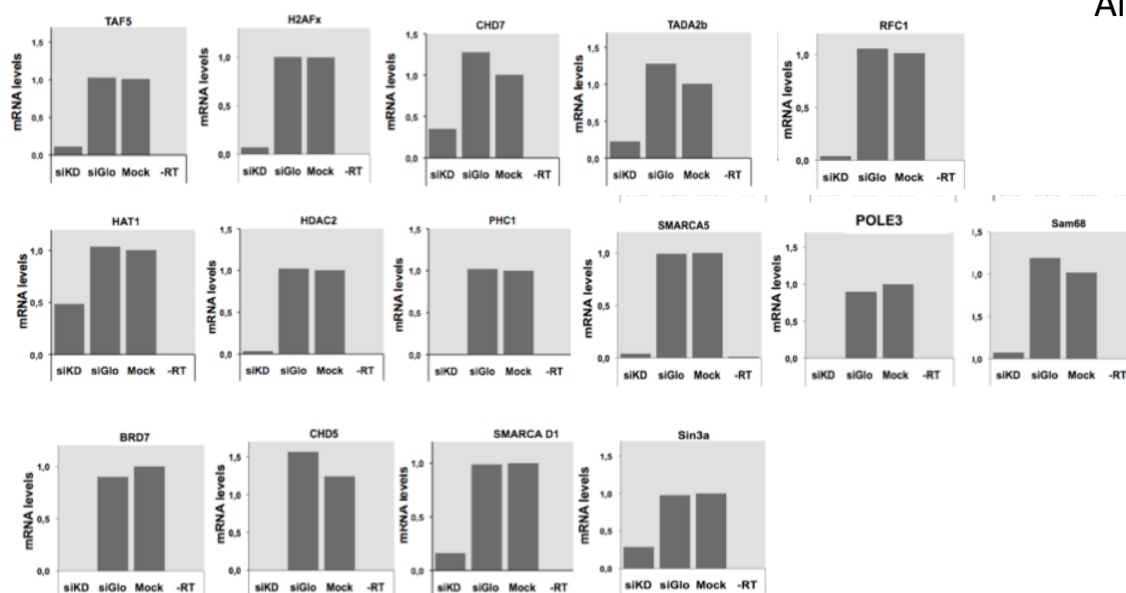

B

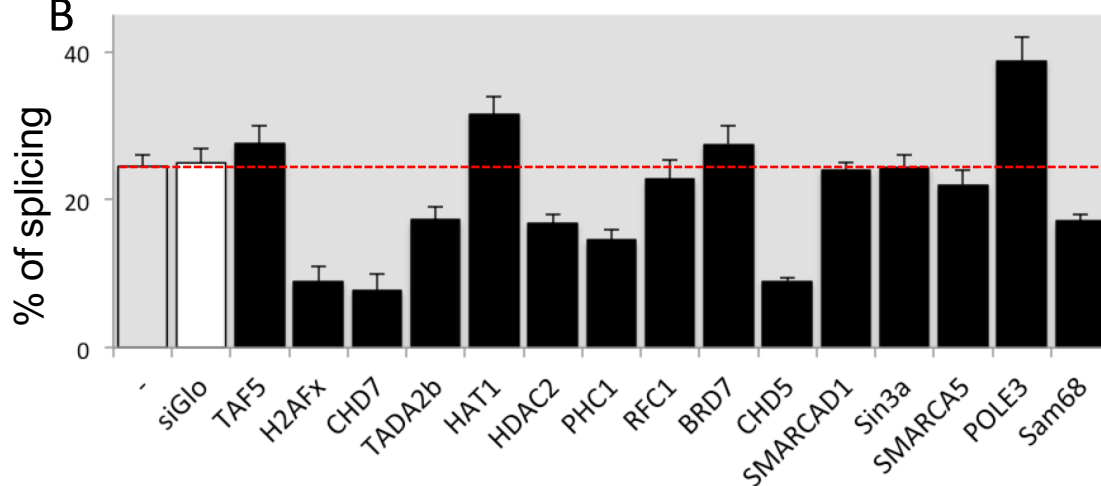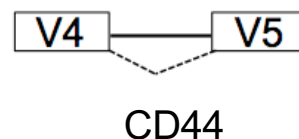

C

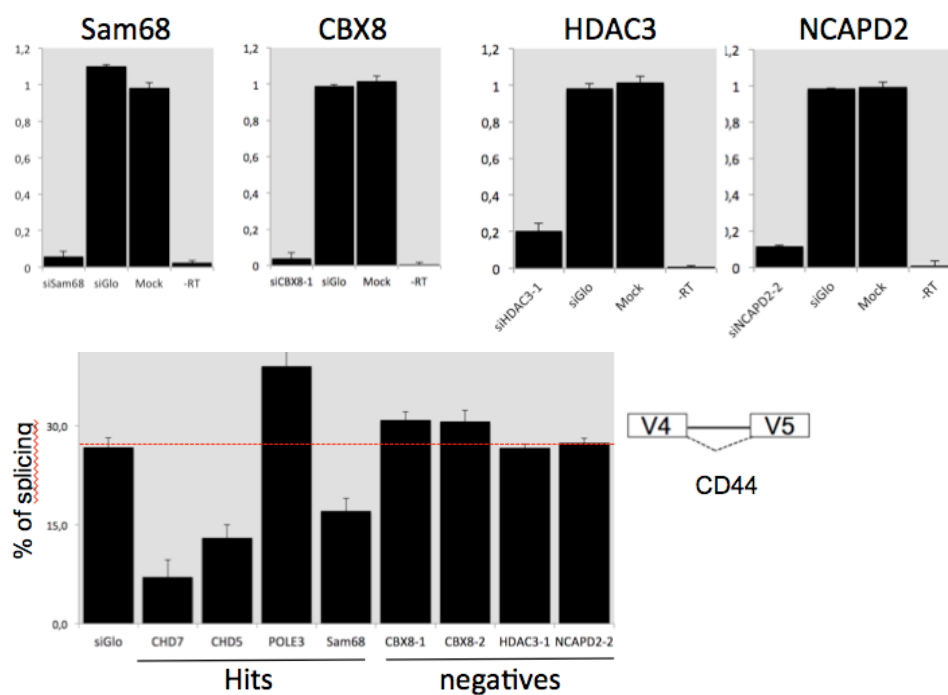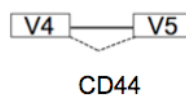

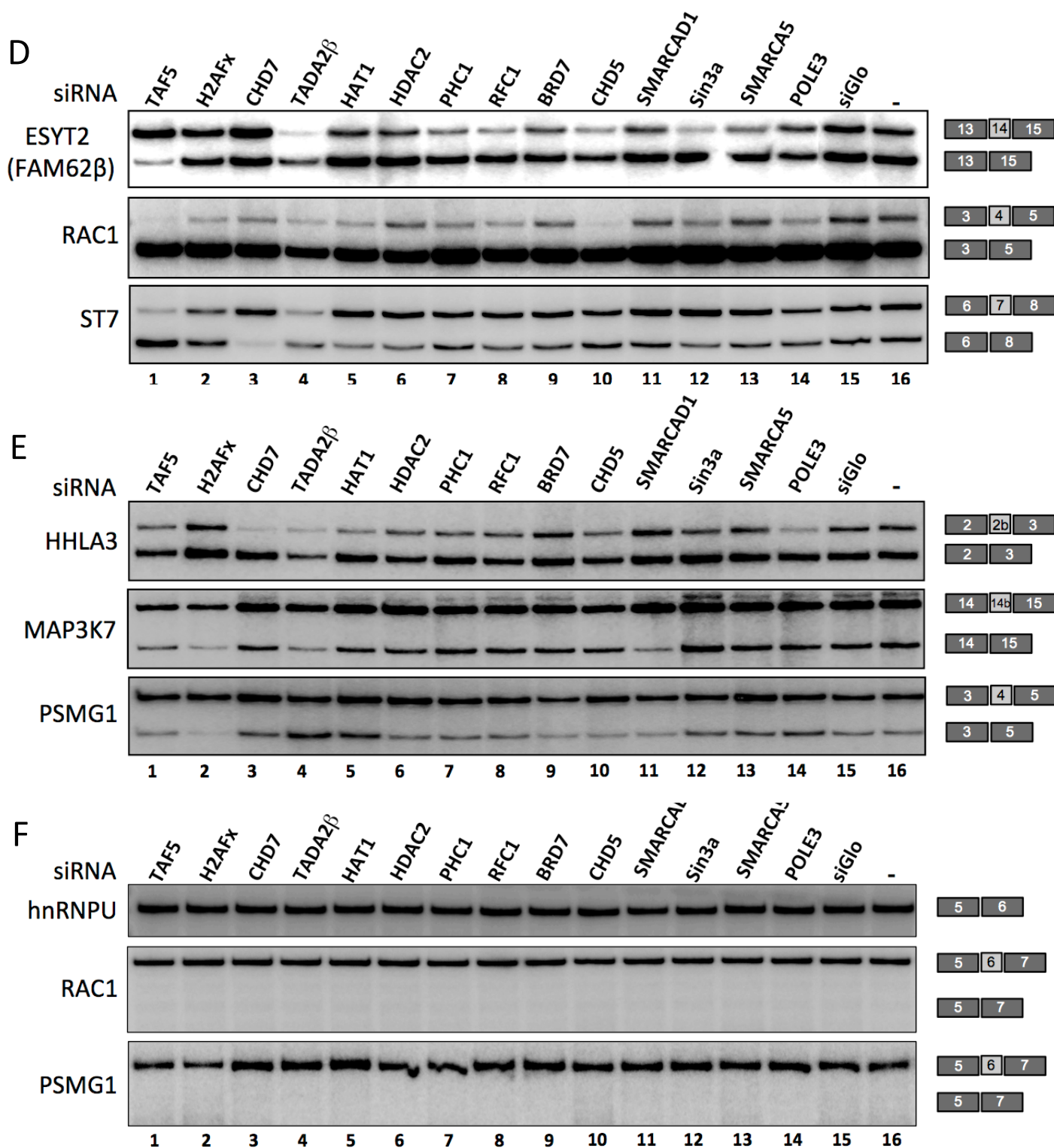

G

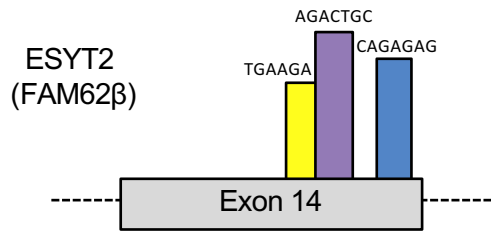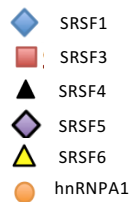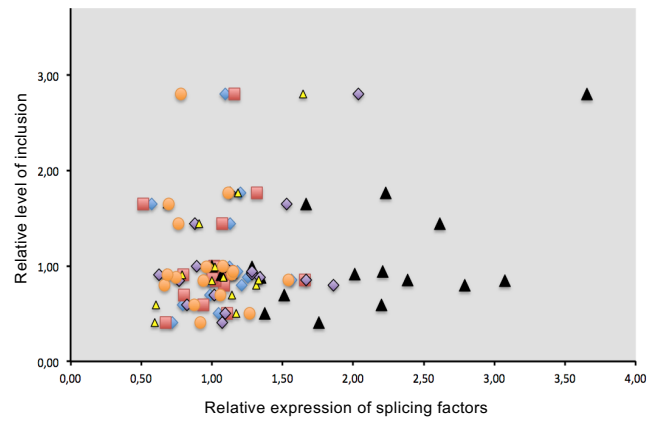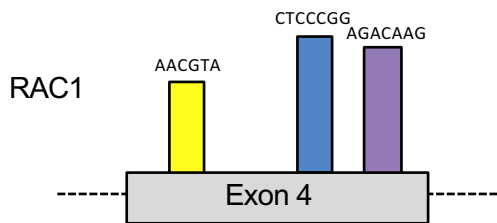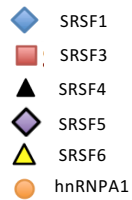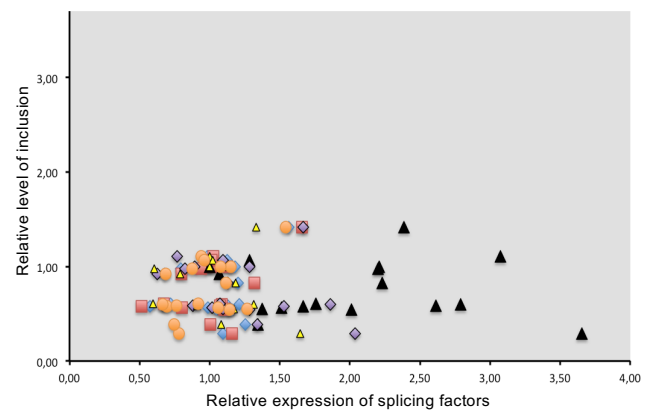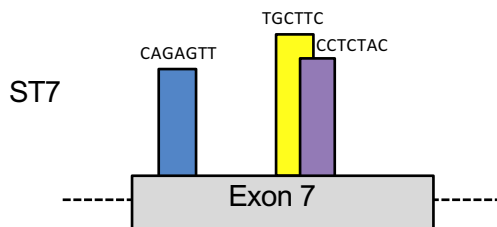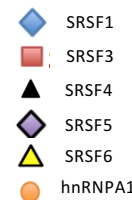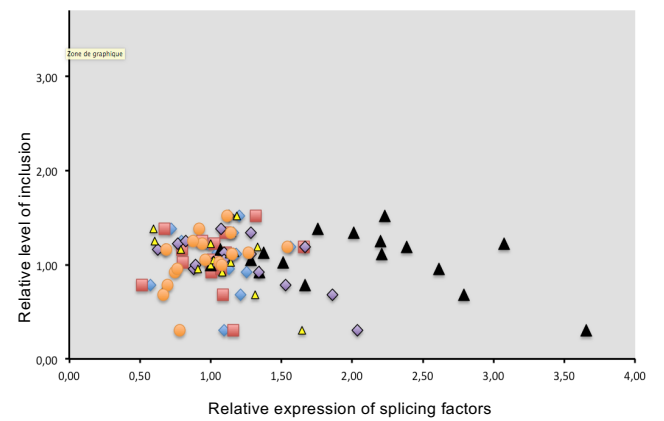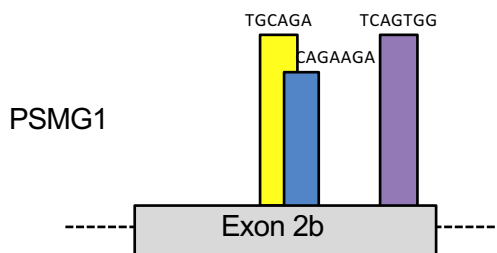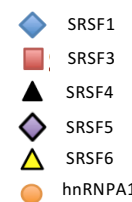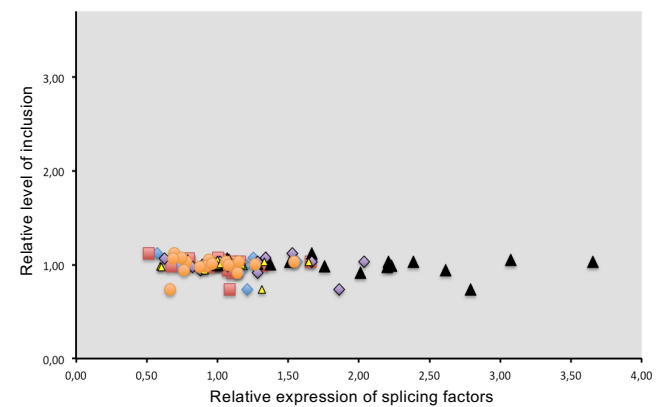

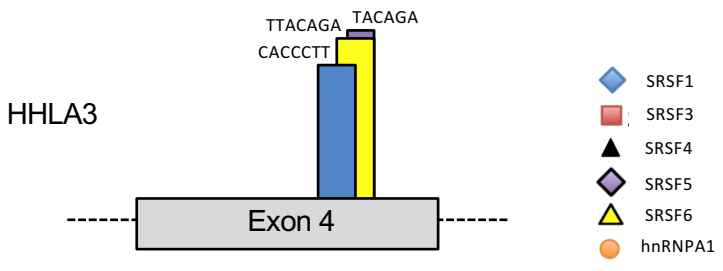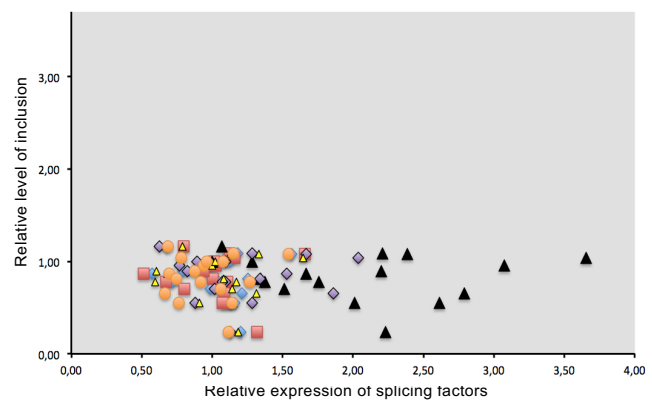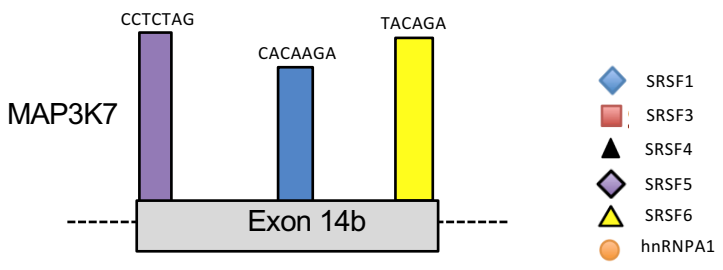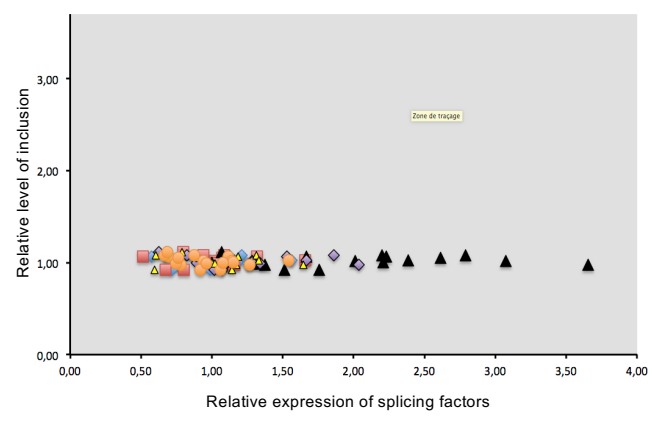

Supplement: S3 Fig — (A) The efficiency of gene silencing using specific siRNA was assessed by RT-qPCR using as control samples untreated or treated with siGlo. (B) Percentage of exon v4-v5 splicing displayed in Fig 3A. The percentages and standard deviations (Stdv) were derived from three independent experiments. The red discontinuous line shows the splicing percentage of exons v4-v5 in untreated and siGlo treated cells. (C) Analysis for splicing of exon v4-v5 in HeLa cells treated with several siRNA targeting chromatin factors retained as hits (CHD7, CHD5, POLE3) or unselected in our screen (CBX8, HDAC3 and NCAPD2). The efficiency of siRNAs for each factor is displayed in the top, while the percentage of exon v4-v5 splicing is presented at the bottom. (D and E) Radiolabeled RT-PCR was used to examine the inclusion of exons reported as sensitive to U2 snRNP activity. PCR products were separated in undenaturing acrylamide gel, then dried and exposed for their detection by phosphoimager. A draw on the right of each gel displays the exons composition associated to bands. (F) Similar analysis to D and E but applied to RAC1, PSMG1 and hnRNPU constitutive exons. (G) The effect of the chromatin factors on splicing cannot be correlated with their effect on the expression of the splicing regulators SRSF1, SRSF3, SRSF4, SRSF5, SRSF6 and hnRNPA1. Left panels: putative binding sites for SRSF1, SRSF5 and SRSF6 within the indicated exons; the binding sequences and their mapping within the exons were obtained using ESEfinder 3.0 (http://rulai.cshl.edu/cgi-bin/tools/ESE3/esefinder.cgi?process=home). Right panels: levels of indicated splicing regulators was quantified by RT-qPCR after knock-down of each of the chromatin factors examined in Fig 3A. Fold-change was then calculated relative to siGlo and plotted as a function of the effect of the chromatin factor on splicing. The scatter plots show an absence of correlation between splicing regulator expression and inclusion of exons showed in samples of F [file pgen.1006318.s003.pdf]

A

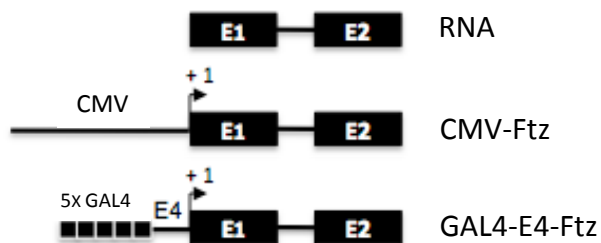

B

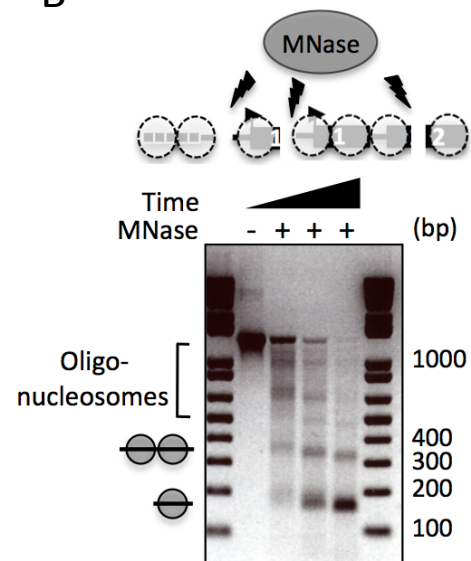

C

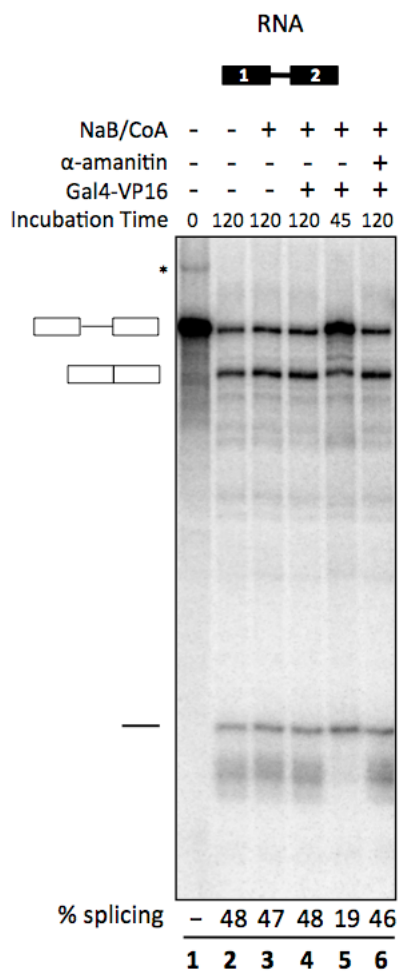

D

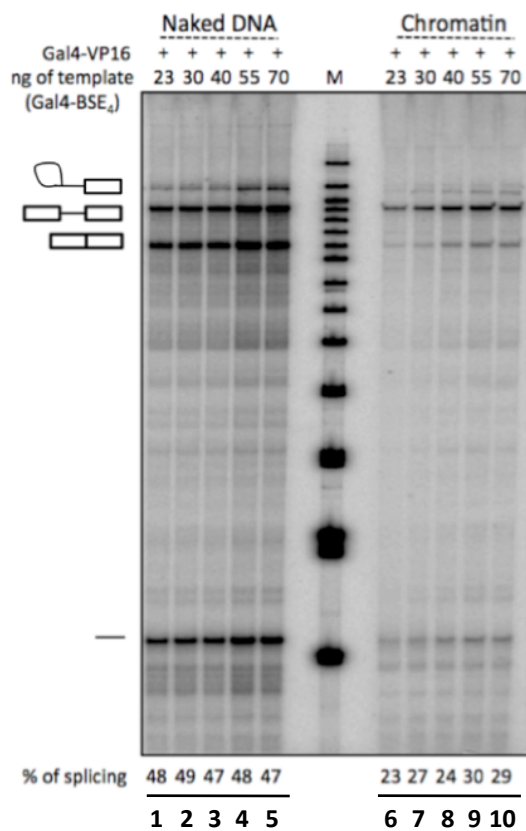

E

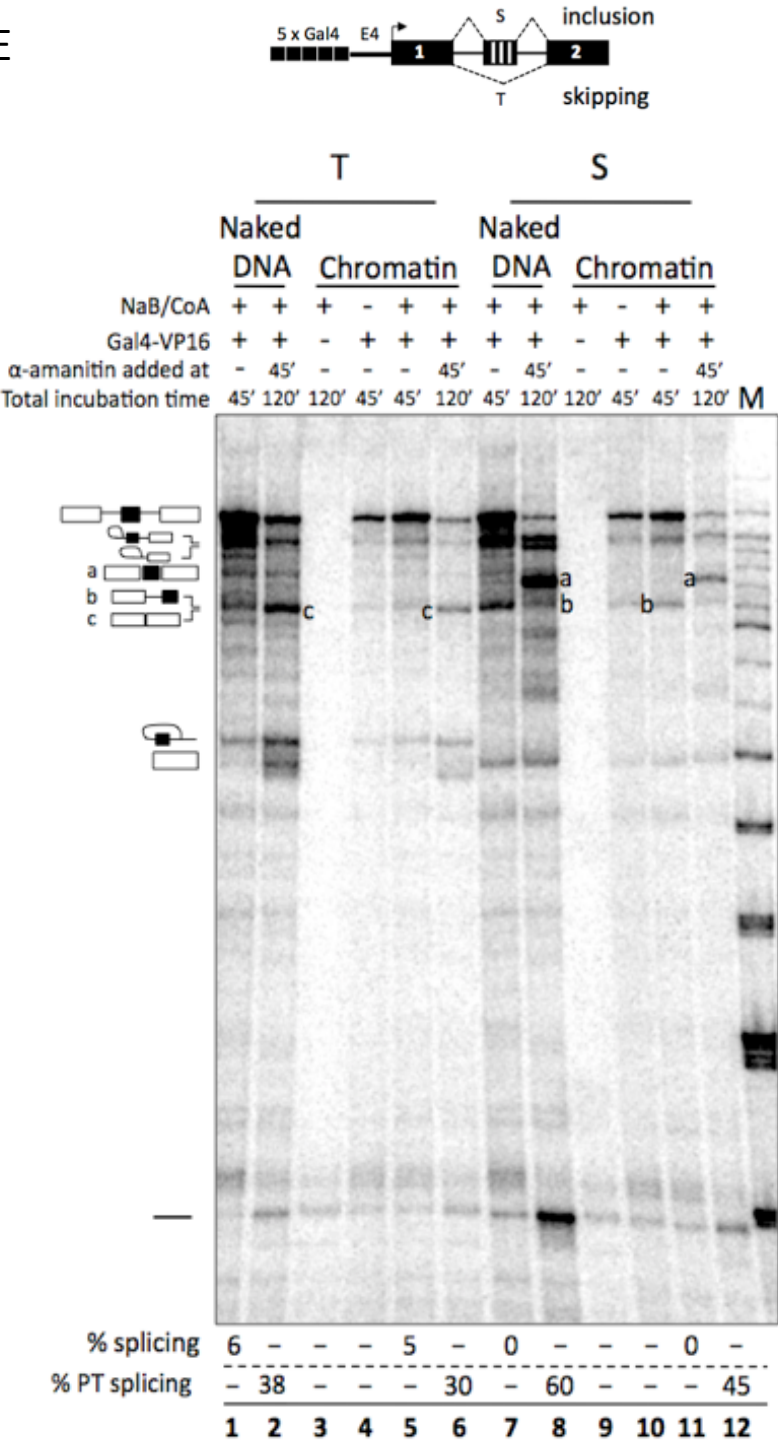

Supplement: S4 Fig — (A) Diagram of RNA and DNA reporter templates with two exons. (B) MNase footprint analysis of an in vitro-assembled polynucleosomal DNA template. DNA markers, mono- and multimers of nucleosome are indicated. (C) Gal4-VP16, NaB/CoA, and α-amanitin were evaluated for their influence on the regulation of splicing, independently of their role in transcription. They were added to in vitro splicing reactions (lanes 3–6) with the RNA version of the reporter for 120 or 45 min as indicated. Then, the percentage of splicing was compared to the basic conditions (lanes 1, 2). (D) Various amount of naked or chromatinized templates were added in transcription-splicing reactions in order to assess potential splicing variation brought by templates. On the top of gel, the quantity of templates added in each reaction is reported, while the percentage of splicing calculated as in the Fig 4B is displayed at the bottom. All reactions were designed with the GAL4-E4-Ftz reporter. (E) Similar experiments to the Fig 4E were performed using two other reporters containing 3 exons. The reporter S integrates three copies of an ESE bound by SRSF1 in its central exon, while the reporter T inserted three sequences inactive for splicing. A precise description of S and T sequences could be found in (Labourier E et al., 1999-NAR [36]). The splicing percentage were calculated as in Fig 4E. (PDF) [file pgen.1006318.s004.pdf]
