# Supplementary material for: A Broad Set of Chromatin Factors Influences Splicing
Source: PLoS Genet. 2016 Sep 23;12(9):e1006318. doi: 10.1371/journal.pgen.1006318 (PMC5035054; doi:10.1371/journal.pgen.1006318)
Supplement: S1 Table — Table organized as in [44]. (PDF) [file pgen.1006318.s005.pdf]

SPLICING FACTORS

Complexes	Gene Name	Ensembl Id.	Mr	Uniprot/ swisprot Accession	total	unique	% Coverage	log(e)
**Sm proteins Core of snRNPs**	SmB/B'	ENSG00000125835	24.6	P14678	7	4	34	-32.8
	SmD1	ENSG00000167088	13.3	P62314	13	2	46	-31.5
	SmD2	ENSG00000125743	13.5	P62316	17	4	47	-38.2
	SmD3	ENSG00000100028	13.9	P62318	4	2	23	-14.8
	SmE1	ENSG00000182004	10.8	P62304	3	1	21	-4.1
	SmF1	ENSG00000139343		P62306				
	SmG	ENSG00000143977	8.5	P62308	6	1	11	-2.5
	LSM2	ENSG00000204392	10.8	Q9Y333	1	1	23	-12.3
	LSM3	ENSG00000170860	11.8	P62310	6	3	40	-24.1
	LSM4	ENSG00000130520	15.3	Q9Y4Z0	2	1	7	-2.1
	LSM5	ENSG00000106355		Q9Y4Y9				
	LSM6	ENSG00000164167		P62312				
	LSM7	ENSG00000130332		Q9UK45				
	LSM8	ENSG00000128534	10.4	O95777	6	1	9	-5.8
**Specific proteins of U1 snRNP**	U1-70 / SNRP70	ENSG00000104852	51.5	P08621	17	7	31	-71.7
	U1-A	ENSG00000077312		P09012				
	U1-C / SNRNPC	ENSG00000124562	17.4	P09234	2	1	37	-17.3
**Specific proteins of U2 snRNP**	U2-A'	ENSG00000131876	28.4	P09661	83	15	49	-190.1
	U2-B"	ENSG00000125870		P08579				
	p14	ENSG00000115128	14.6	Q9Y3B4	69	8	94	-102.7
	SF3a60	ENSG00000183431	58.8	Q12874	155	19	55	-227.6
	SF3a66	ENSG00000104897	49.2	Q15428	34	8	45	-71.1
	SF3a120	ENSG00000099995	88.8	Q15459	145	25	43	-341.4
	SF3b10 / SF3B5	ENSG00000169976	10.1	Q9BWJ5	17	4	69	-48.4
	SF3b14b	ENSG00000100410	12.4	Q7RTV0	31	6	65	-62.6
	SF3b49	ENSG00000143368	44.4	Q15427	27	6	30	-81.2
	SF3b130	ENSG00000189091	135.5	Q15393	168	30	40	-429.4
	SF3b145	ENSG00000087365	100.2	Q13435	191	36	61	-410.1
	SF3b155	ENSG00000115524	145.7	O75533	311	48	45	-610.3
**17S U2snRNP proteins related**	U2AF65	ENSG00000063244	53.5	P26368	9	2	12	-14.8
	U2AF35	ENSG00000160201	27.9	Q01081	6	2	8	-25.1
	SPF45	ENSG00000134453	44.9	Q96I25	20	10	33	-103.5
	CHERP	ENSG00000085872	103.6	Q8IWX8	28	11	24	-128.8
	fSAPa - SR140	ENSG00000163714	118.2	O15042	59	22	46	-214.5
	SF3b125 / DDX42	ENSG00000198231	102.9	Q86XP3	12	5	8	-51.4
	PrP43	ENSG00000109606	90.9	O43143	104	24	35	-257.4
**Specific proteins of U5 snRNP**	PRP8 (U5-220)	ENSG00000174231	273.4	Q6P2Q9	303	77	42	-943.8
	U5-200 (Brr2)	ENSG00000144028	244.4	O75643	254	58	32	-678.4
	U5-116 (Snu114)	ENSG00000108883	109.4	Q15029	174	30	36	-372.5
	U5-102 (Prp6)	ENSG00000101161	106.9	O94906	181	30	41	-385.7
	U5-100 (Prp28)	ENSG00000174243	95.5	Q9BUQ8	59	15	24	-159.8
	U5-52	ENSG00000169217	37.6	O95400	10	2	9	-21.5
	U5-40	ENSG00000060688	39.3	Q96DI7	43	11	45	-145.5
	U5-15	ENSG00000141759		P83876				
**Specific proteins of U4/U6 snRNPs**	U4/U6-90(PRP3)	ENSG00000117360	77.5	O43395	42	16	35	-173.1
	U4/U6-60(PRP4)	ENSG00000136875	58.4	O43172	87	24	70	-279.3
	U4/U6-61- PRPF31	ENSG00000105618	55.4	Q8WWY3	22	9	38	-97
	U4/U6-20	ENSG00000171960	19.2	O43447	48	3	20	-25.4
	U4/U6-15.5	ENSG00000100138	14.2	P55769	16	5	34	-51.9
**Specific proteins of the Tri-SnRNPs U4/U6-U5**	Tri-snRNP 110 - Prp24 - SART	ENSG00000175467	90.2	O43290	110	31	51	-396.3
	Tri-snRNP 65 - SAD1	ENSG00000168883	65.3	Q53GS9	17	7	20	-72.2
	Tri-snRNP 27	ENSG00000124380	18.8	Q8WVK2	1	1	30	-1.9
	PRPF38A / PrP38	ENSG00000134748	37.5	Q8NAV1	10	2	14	-26.4
	TFIP11 / TIP39	ENSG00000100109	96.8	Q9UBB9	15	8	15	-67.5
**SR proteins**	SRp75	ENSG00000116350		Q08170				
	p54/SFRS11	ENSG00000116754	53.5	Q05519	6	2	28	-23.4
	SRp55	ENSG00000124193	39.6	Q13247	4	2	15	-14.2
	SRp40	ENSG00000100650		Q13243				
	SF2/ASF	ENSG00000136450	27.7	Q07955	9	5	31	-43.9
	9G8	ENSG00000115875	27.4	Q16629	32	7	46	-64.9
	SC35	ENSG00000161547		Q01130				
	SRp30c	ENSG00000111786	25.5	Q13242	6	2	14	-12.4
	hTra2	ENSG00000136527	33.6	P62995	7	3	24	-23.7
	SRp20	ENSG00000112081	19.3	P84103	3	2	33	-24.9
	SRp38 / BX511012.1	ENSG00000215699		O75494				
	SRSF8 / SRp46	ENSG00000180771						
**SR proteins related**	SRm300	ENSG00000167978	299.4	Q9UQ35	105	35	28	-407.3
	SRm160	ENSG00000133226	102.3	Q8IYB3	3	2	4	-10.9

**hnRNPs**	hnRNP A0	ENSG00000177733	30.8	Q13151	2	2	20	-16.4
	hnRNP A1	ENSG00000135486	38.7	P09651	23	6	11	-85.2
	hnRNP A2/B1	ENSG00000122566	37.4	P22626	80	15	63	-173.6
	hnRNP A3	ENSG00000170144	39.6	P51991	7	4	33	-41.9
	hnRNP C1/C2	ENSG00000092199	33.6	P07910	97	13	51	-175.1
	hnRNP D0	ENSG00000138668	38.4	Q14103	3	2	14	-15.4
	hnRNP I/PTB	ENSG00000011304	57.2	P26599	4	1	2	-2
	hnRNP K	ENSG00000165119	50.9	P61978	7	4	20	-47.8
	hnRNP F	ENSG00000169813	45.6	P52597	5	3	13	-45.7
	hnRNPH1	ENSG00000169045	49.2	P31943	24	4	17	-52.5
	hnRNPG	ENSG00000147274	42.3	P38159	40	12	39	-112.1
	hnRNPM	ENSG00000099783	77.5	P52272	43	15	31	-185.6
	hnRNPU	ENSG00000153187	90.5	Q00839	43	14	34	-138.9
	hnRNP L	ENSG00000104824	64.1	P14866	14	6	20	-74.3
	hnRNP R	ENSG00000125944	70.9	O43390	18	6	14	-62.4
	hnRNP RALY	ENSG00000125970	32.4	Q9UKM9	11	7	16	-57.1
	HNRNPAB	ENSG00000197451	36.2	Q99729	2	1	4	-4
	RBMXL2	ENSG00000170748		O75526				
	HNRNPH2	ENSG00000126945		P55795				
	HNRNPH3	ENSG00000096746	36.9	P31942	2	1	4	-2.8
	SYNCRIP / GRY-BP	ENSG00000135316	69.6	O60506	2	1	3	-21.7
	PCBP1	ENSG00000169564	37.5	Q15365	7	2	10	-16.7
	PCBP2	ENSG00000197111		Q15366				
**PrP19/CDC5L complex**	CDC5L	ENSG00000096401	92.2	Q99459	26	7	15	-74
	NAP	ENSG00000132792		Q8WYA6				
	CCAP1 / HSP73 / HSP71	ENSG00000109971	70.9	P11142	69	15	35	-163.1
	CCAP2 / CWC15 / Hspc148	ENSG00000150316	26.6	Q9P013	9	2	27	-11.7
	PRL1 (Prp46)	ENSG00000171566	57.2	O43660	11	5	18	-40.3
	PrP19	ENSG00000110107	55.1	Q9UMS4	38	11	30	-142.4
	WBP11 / Npw38BP	ENSG00000084463	70	Q9Y2W2	5	3	11	-20.3
	SPF27 - BCAS2	ENSG00000116752	26.1	O75934	20	5	36	-50.7
**PrP19/CDC5L complex related**	SKIP	ENSG00000100603		Q13573				
	fSAP17(G10 homolgue)	ENSG00000106245	17	P41223	2	1	11	-2.4
	XAB2 / hSYF1 /(HCNP)	ENSG00000076924	99.9	Q9HCS7	22	10	17	-108
	PPIE (CypE)	ENSG00000084072	33.4	Q9UNP9	2	1	4	-1.8
	ISY1	ENSG00000240682	33	Q9ULR0	9	4	17	-37.4
	AQR / KIAA0560 / fSAP164	ENSG00000021776	171.2	O60306	28	9	8	-82.7
	CRNKL1 / hSYF3 / Cm	ENSG00000101343	100.4	Q9BZJ0	9	4	8	-34.9
	PPIL1	ENSG00000137168	18.2	Q9Y3C6	4	2	24	-25
	fSAP47 - RBM22 - ECM2	ENSG00000086589	46.9	Q9NW64	12	5	19	-43.1
**EJC/mRNP**	RNPS1	ENSG00000205937	34.2	Q15287	13	2	14	-31.7
	Aly (REF) (THO4)	ENSG00000183684	26.9	Q86V81	35	8	54	-97.6
	Y14	ENSG00000131795	19.9	Q9Y5S9	6	3	19	-26.5
	Magoh	ENSG00000111196	17.3	Q96A72	10	4	37	-32.3
	IF4N (eIF4a3) DDX48	ENSG00000141543	46.8	P38919	56	13	32	-159.7
	Pinin	ENSG00000100941	81.6	Q9H307	55	15	32	-157.6
	UAP56	ENSG00000198563	49	Q13838	74	14	31	-151.6
	fSAP152(Acinus)	ENSG00000100813		Q9UKV3				
	fSAP18	ENSG00000123144		Q9BQ61				
**Step 2 factors**	PrP16	ENSG00000140829	140.4	Q92620	1	1	2	-4.6
	PrP22 (DHX8)	ENSG00000067596	139.2	Q14562	15	6	9	-61.3
	Prp17	ENSG00000168438	65.5	O60508	11	5	17	-48.2
	Prp18	ENSG00000165630		Q99633				
	Slu7	ENSG00000164609		O95391				
**Factors recruited to B complex**	Prp4kinase	ENSG00000112739	116.9	Q13523	24	9	18	-100.5
	RED	ENSG00000113141	65.6	Q13123	46	15	42	-137.6
	MFAP1	ENSG00000140259	51.9	P55081	23	8	36	-78.9
	PABPC1 / PAB2 / PABPN1	ENSG00000070756	70.6	P11940	7	4	8	-28.3
	RNF113A / ZNF183	ENSG00000125352		O15541				
	SKIV2L2 / fSAP118	ENSG00000039123	117.7	P42285	4	2	3	-16.3
	THRAP3 / THRAP150	ENSG00000054118	108.6	Q9Y2W1	56	15	28	-192.3
	UBL5	ENSG00000198258		Q9BZL1				
	HSPB1 / HSP27	ENSG00000106211	22.8	P04792	9	4	27	-30.9
	Cyp60 / PPIL2	ENSG00000100023		Q13356				
	fSAP57 - Smu-1	ENSG00000122692	57.5	Q2TAY7	52	11	31	-136.2
	fSAPc-NY-REN-24 / C19orf29	ENSG00000105298	88.6	Q8WUQ7	2	1	2	-11.5
	MGC23918 (CCDC12)	ENSG00000160799	19.2	Q8WUD4	8	1	11	-2.3
	HsKin17	ENSG00000151657	45.3	O60870	4	1	5	-4.9
	GCFC1 / fSAP105 / C21orf66	ENSG00000159086	104.7	Q9Y5B6	4	2	4	-16.5
	CWC22 / EIF4GL / fSAPb / KI	ENSG00000163510		Q9HCG8				
	ZNF830 / CCDC16 / OMCG1	ENSG00000198783		Q96NB3				
	p68	ENSG00000108654	69.1	P17844	13	7	22	-88.4

**Factors recruited to A complex**	fSAP59 - HCC1 - RNPC2	ENSG00000131051	59.3	Q14498	56	9	32	-103.1
	HuR	ENSG00000066044	36.1	Q15717	3	2	13	-12
	p72 - DDX17	ENSG00000100201	80.2	Q92841	24	14	30	-143.4
**Factors binding pre-mRNA and RNA**	CBP20	ENSG00000114503		P52298				
	CBP80	ENSG00000136937	91.8	Q09161	8	5	10	-42.6
	DDX3X	ENSG00000215301	73.2	O00571	5	3	8	-29.4
	YB1	ENSG00000065978		P67809				
	NF45/ILF2	ENSG00000143621	43	Q12905	5	4	16	-36.6
	ASR2	ENSG00000087087	100.6	Q9BXP5	9	7	18	-54.3
	DBPA	ENSG00000060138		P16989				
	HSP70 / HSPA2	ENSG00000126803		P54652				
	ZC3H18	ENSG00000158545	106.3	Q86VM9	30	10	24	-110.6
	BCLAF1	ENSG00000029363	106.1	Q9NYF8	29	10	21	-93.7
	RBM7	ENSG00000076053		Q9Y580				
**Factors recruited to C complex**	Abstrakt	ENSG00000183258	69.8	Q9UJV9	4	2	4	-12.1
	DDX35	ENSG00000101452		Q9H5Z1				
	GCIP-IP (CCNDBP1)	ENSG00000117614	28.7	O95926	4	2	16	-13.5
	CyP64 / PPWD1	ENSG00000113593		Q96BP3				
	PPIL3	ENSG00000115934	18.1	Q9H2H8	5	1	8	-4.6
	FRG1	ENSG00000109536		Q14331				
	eNOS-IP	ENSG00000142546		Q9Y314				
	GPKOW / T54 / GPATC5 / Sp	ENSG00000068394		Q92917				
	FAM32A	ENSG00000105058	13.2	Q9Y421	2	1	15	-2.1
	GNB2L1 / RACK1 / H12.3	ENSG00000204628		P63244				
	C1orf55 / FLJ35382	ENSG00000143751		Q6IQ49				
	RUVBL1 / TIP49	ENSG00000175792	50.2	Q9Y265	8	3	10	-33.7
	GPATCH1 / FLJ10805	ENSG00000076650	103.3	Q9BRR8	3	2	5	-14.5
**TREX complex**	HPR1 - THOC1	ENSG00000079134	75.6	Q96FV9	53	10	28	-134.9
	THOC2	ENSG00000125676	182.7	Q8NI27	60	23	26	-251.4
	TEX1(THOC3)	ENSG00000051596	38.7	Q96J01	27	5	21	-44.8
	THOC5 / fSAP79 /C22orf19 (H	ENSG00000100296	78.5	Q13769	17	7	11	-68.4
	THOC6 / fSAP35	ENSG00000131652	37.5	Q86W42	39	7	24	-83.9
**hRES complex protein**	SNIP1	ENSG00000163877	45.8	Q8TAD8	2	1	6	-4.6
	RBMX2 / CGI-79	ENSG00000134597	37.3	Q9Y388	1	1	7	-2
	BUD13 / fSAP71 / MGC13125	ENSG00000137656	70.5	Q9BRD0	1	1	3	-3
**Potential C-complex specific proteins**	PPIG	ENSG00000138398		Q13427				
	FAM50A	ENSG00000071859		Q14320				
	FAM50B	ENSG00000145945		Q9Y247				
	C9orf78	ENSG00000136819		Q9NZ63				
	FRA10AC1	ENSG00000148690		Q70Z53				
	CXorf56	ENSG00000018610		Q9H5V9				
	DGCR14 / DGSI	ENSG00000100056	52.5	Q96DF8	3	2	8	-10.5
	CCDC130	ENSG00000104957		P13994				
	TOE1	ENSG00000132773		Q96GM8				
	NKAP	ENSG00000101882		Q8N5F7				
	ZCCHC10	ENSG00000155329		Q8TBK6				
	CDK10	ENSG00000185324		Q15131				
	TTC14	ENSG00000163728		Q96N46				
	WDR70	ENSG00000082068		Q9NW82				
	NFKBIL1	ENSG00000204498		Q9UBC1				
	JUP	ENSG00000173801		P14923				
**Miscellous factors**	BAG2	ENSG00000112208	23.8	O95816	11	4	27	-37.3
	RBBP6	ENSG00000122257	201.4	Q7Z6E9	9	4	7	-41.5
	RBM42	ENSG00000126254		Q9BTD8				
	PUF60	ENSG00000179950	59.8	Q9UHX1	16	7	30	-65.1
	PrP5	ENSG00000145833	117.3	Q7L014	114	29	42	-327.5
	SF1	ENSG00000168066	68.3	Q15637	4	2	13	-10.6
	SPF30 - SMNC1	ENSG00000119953	26.7	O75940	20	5	58	-52.9
	FBP11(FNBP3) / PRPF40A	ENSG00000196504	108.7	O75400	29	9	26	-85.6
	CA150 / TCERG1	ENSG00000113649	123.8	O14776	18	8	20	-83
	SPF31	ENSG00000126698	29.8	O75937	4	2	13	-15.9
	PP2Cg	ENSG00000115241		O15355				
	fSAP94 - RBM25	ENSG00000119707	100.1	P49756	42	14	44	-140.3
	SHARP - HDAC1	ENSG00000065526	402	Q96T58	12	8	5	-61.8
	TAT-SF1	ENSG00000102241	85.8	O43719	65	14	18	-168.3
	OTT - RBM15	ENSG00000162775	107.1	Q96T37	16	8	14	-61.7
	CF I-68	ENSG00000111605		Q16630				
	IMP3 / IGF2BP3	ENSG00000136231		O00425				
	CIRP	ENSG00000099622		Q14011				
	RHA (DDX9)	ENSG00000135829	140.9	Q08211	5	3	5	-17.8
	fSAP113	ENSG00000155363		Q9HCE1				

	LUC7A	ENSG00000108848	51.4	O95232	15	5	24	-55.5
	FBP3	ENSG00000107164		Q96I24				
	CrkRS	ENSG00000167258		Q9NYV4				
	CF I-25	ENSG00000167005	26.2	O43809	4	2	18	-11.7
	ZNF207	ENSG00000010244	50.7	O43670	9	3	44	-23.5
	fSAP24 / THOC7	ENSG00000163634	23.7	Q6I9Y2	25	6	31	-46.7
	WTAP	ENSG00000146457	44.2	Q15007	25	10	49	-113
	fSAP121	ENSG00000164944	201.9	Q69YN4	21	12	12	-134.4
	fSAP33 (ISY1)	ENSG00000172780		Q86YS6				
	fSAP23 (TFIIB) C16orf80	ENSG00000070761		Q9Y6A4				
	DHX16	ENSG00000226171	119.2	O60231	4	1	1	-1.6
	PSF	ENSG00000116560	76.1	P23246	3	2	6	-19.1
	FLJ31121 / ZMAT2	ENSG00000146007	23.6	Q96NC0	1	1	9	-1.9
	CPSF1 (160kD)	ENSG00000071894	160.8	Q10570	29	10	11	-82.7
	HDB/DICE1	ENSG00000102786		Q9UL03				
	SDCCAG10	ENSG00000153015		Q6UX04				
	eIF-4G 3 (KIAA1604)	ENSG00000075151	176.5	O43432	1	1	1	-1.6
	FLJ10374	ENSG00000105248		Q9BW85				
	FLJ10634	ENSG00000104129		Q9NVM6				
	SFRS14-SWAP-(SF4)	ENSG00000105705	72.4	Q8IWZ8	8	5	16	-46
	RBM5	ENSG00000003756	92.1	P52756	18	6	17	-74.8
	E1B-AP5	ENSG00000105323	95.7	Q9BUJ2	4	3	8	-18.9
	PM/Scl 2	ENSG00000171824	100.8	Q01780	3	3	8	-29.9
	ZCCHC-CD8 (FLJ90157)	ENSG00000033030		Q6NZY4				
	NuMA (SP-H antigen)	ENSG00000137497	238.1	Q14980	6	2	2	-24.3
	FLJ10839	ENSG00000138336		Q8NFU7				
	FLJ21007	ENSG00000083544		Q9H7E2				
**Other factors associated to spliceosome**	DnaJ (Hsp40) / DNAJC6	ENSG00000116675		O75061				
	SMC1	ENSG00000072501		Q14683				
	SMC2	ENSG00000136824		O95347				
	NFAR-2 / ILF3	ENSG00000129351	95.3	Q12906	9	4	10	-33.6
	TLS-FUS	ENSG00000089280	53.4	P35637	13	6	48	-48
	Matrin3	ENSG00000015479	94.6	P43243	37	13	24	-131.5
	hBUB3	ENSG00000154473	37.1	O43684	6	5	34	-44.9
	TAFII68	ENSG00000172660		Q92804				
	GRP78	ENSG00000044574	72.3	P11021	2	1	3	-13.5
	Ku70	ENSG00000196419		P12956				
	PABPC4	ENSG00000090621	70.7	Q13310	2	1	2	-15.5
	NRIP2	ENSG00000053702		Q9BQI9				
	RBM10	ENSG00000182872	103.5	P98175	49	17	32	-188.1
	WBP4	ENSG00000120688	42.5	O75554	7	3	9	-18.6
	ARGLU1	ENSG00000134884		Q9NWB6				
	SREK1	ENSG00000153914	59.3	Q8WXA9	7	4	47	-39.7
	BRPF3	ENSG00000096070		Q9ULD4				
	DCD	ENSG00000161634		P81605				
	DDX19B	ENSG00000157349		Q9UMR2				
	TBL1XR1	ENSG00000177565	55.6	Q9BZK7	7	4	20	-34.3
	DSP	ENSG00000096696		P15924				
	DNAJA1	ENSG00000086061		P31689				
	GEMIN5	ENSG00000082516		Q8TEQ6				
	SLTM	ENSG00000137776	117.1	Q9NWH9	4	3	6	-28.6
	TARDBP	ENSG00000120948	44.7	Q13148	9	4	25	-39.9
	ANXA1	ENSG00000135046		P04083				
	C19orf53	ENSG00000104979		Q9UNZ5				
	DHX57	ENSG00000163214		Q6P158				
	FBL	ENSG00000105202	33.8	P22087	4	3	20	-20.6
	GNL3	ENSG00000163938		Q9BVP2				
	GPATCH3	ENSG00000198746		Q96I76				
	INTS5	ENSG00000185085		Q6P9B9				
	SAFB2	ENSG00000130254	107.4	Q14151	26	10	18	-88.6
	TFE3	ENSG00000068323		P19532				
	YTHDC1	ENSG00000083896	84.6	Q96MU7	9	2	4	-14.6
	ZCRB1	ENSG00000139168		Q8TBF4				
	CREBBP	ENSG00000005339		Q92793				
	SEC31A	ENSG00000138674		O94979				
	CRIPT	ENSG00000119878		Q9P021				
	HSPA6	ENSG00000173110	71	P17066	3	2	6	-41.5
	SRSF12	ENSG00000154548	30.5	Q8WXF0	1	1	14	-11.5
	FNBP4	ENSG00000109920	110.2	Q8N3X1	4	3	9	-18.9
	RBM4	ENSG00000173933		Q9BWF3				
	DDX21	ENSG00000165732		Q9NR30				
	ERH	ENSG00000100632	12.3	P84090	40	6	45	-60.6

	USP42	ENSG00000106346		Q9H9J4				
