# Supplementary material for: A Broad Set of Chromatin Factors Influences Splicing
Source: PLoS Genet. 2016 Sep 23;12(9):e1006318. doi: 10.1371/journal.pgen.1006318 (PMC5035054; doi:10.1371/journal.pgen.1006318)
Supplement: S2 Table — (PDF) [file pgen.1006318.s006.pdf]

NCBI gene symbo	mRNA Accessions	siRNA Target Sequence	Gene Description	Product Id	Product Name
**ACIN1**	NM_014977	**AAGGAGAAAGACGATCATCTA**	apoptotic chromatin condensation inducer 1	SI00290871	Hs_ACIN1_1
		**TCCGAATATATTCGATTCAAA**		SI00290878	Hs_ACIN1_2
		**ATGGAAAGAATTGATTGGTAA**		SI00290885	Hs_ACIN1_3
**ACOX2**	NM_003500	**CTGCCTATGAGGAATATATAA**	acyl-Coenzyme A oxidase 2, branched chain	SI00290990	Hs_ACOX2_2
		**CACCATCATGTGATAATGGTA**		SI00290997	Hs_ACOX2_3
		**ATGGCATATATGCAACATTAA**		SI00290983	Hs_ACOX2_1
**ACTL6A**	NM_004301 NM_177989 NM_178042	**AAAGCTTTAACTGGCTCTATA**	actin-like 6A	SI02779987	Hs_ACTL6A_5
		**TACGAGGTCTTGGCACAATTA**		SI00062405	Hs_ACTL6A_1
		**CAACAGTGGAACGGAGGTTTA**		SI02779994	Hs_ACTL6A_6
**ACTL6B**	NM_016188	**CAACAGCACCATGGAGCGCAA**	actin-like 6B	SI00114226	Hs_ACTL6B_1
		**CACGGCCATTCCAGTACATGA**		SI00114247	Hs_ACTL6B_4
		**CACCTACAGCAAACACGTCAA**		SI00114240	Hs_ACTL6B_3
		**CAACAGCACCATGGAGCGCAA**		SI00114226	Hs_ACTL6B_1
**AOF2**	NM_001009999 NM_015013	**CTGGAAATGACTATGATTTAA**	amine oxidase (flavin containing) domain 2	SI02780932	Hs_AOF2_5
		**ACATCTTACCTTAGTCATCAA**		SI00109102	Hs_AOF2_2
		**AGGCCTAGACATTAAACTGAA**		SI02781177	Hs_AOF2_6
**ARID1A**	NM_006015 NM_018450 NM_139135	**CACCTTGGTTACACTCGCCAA**	AT rich interactive domain 1A (SWI-like)	SI00083713	Hs_ARID1A_2
		**ATGGCGGGAACTTGCAACCAA**		SI03051461	Hs_ARID1A_5
		**CAGAGTTTACTCTGTACGAAT**		SI00083727	Hs_ARID1A_4
		**CACCTTGGTTACACTCGCCAA**		SI00083713	Hs_ARID1A_2
**ARID1B**	NM_017519 NM_020732 NM_175863	**CCGGAGTTTAATAATTACTAT**	AT rich interactive domain 1B (SWI1-like)	SI00302981	Hs_ARID1B_1
		**ATGGCTCAGTATGGACCTCAA**		SI00302995	Hs_ARID1B_3
		**CTCAGGAAGGCTATGGAACTA**		SI03199252	Hs_ARID1B_5
**ARID3A**	NM_005224	**CAGCATGTCGGTGGAGATCAA**	AT rich interactive domain 3A (BRIGHT-like)	SI00303044	Hs_ARID3A_2
		**AAGGATATCTATATATCTATA**		SI00303037	Hs_ARID3A_1
		**CCGCAGGGCCATGAAACTACA**		SI00303058	Hs_ARID3A_4
**ARID3B**	NM_006465	**GACGGTGATCCTGAAAGGAAA**	AT rich interactive domain 3B (BRIGHT-like)	SI00303079	Hs_ARID3B_3
		**CAGGGTATGAGGAGATGAATA**		SI00303086	Hs_ARID3B_4
		**AAGCTTGAGCACAAACAGATA**		SI00303072	Hs_ARID3B_2
**ARID4A**	NM_002892 NM_023000 NM_023001	**ATGGCTTATCTAAGACAGCAA**	AT rich interactive domain 4A (RBP1-like)	SI00045402	Hs_ARID4A_1
		**ACGATTGAAGTTGATAGTATT**		SI00045409	Hs_ARID4A_2
		**ACGATTGAAGTTGATAGTATT**		SI00045409	Hs_ARID4A_2
		**CTCGAGATGTAAATTCTATTA**		SI00045416	Hs_ARID4A_3
		**CTCGAGATGTAAATTCTATTA**		SI00045416	Hs_ARID4A_3
		**ATGGCTTATCTAAGACAGCAA**		SI00045402	Hs_ARID4A_1
**ARID4B**	NM_016374 NM_031371	**CAGGAAGCTGTTATCAATAAA**	AT rich interactive domain 4B (RBP1-like)	SI00303121	Hs_ARID4B_1
		**AAGGAGGATAATAGCAGTGAA**		SI00303128	Hs_ARID4B_2
		**AAGGAGGATAATAGCAGTGAA**		SI00303128	Hs_ARID4B_2
		**AACAGAGATCAAAGAAATAAA**		SI00303135	Hs_ARID4B_3
		**AACAGAGATCAAAGAAATAAA**		SI00303135	Hs_ARID4B_3
		**CAGGAAGCTGTTATCAATAAA**		SI00303121	Hs_ARID4B_1
**ASF1A**	NM_014034	**AACCATGGATTTAGAAAGGTA**	ASF1 anti-silencing function 1 homolog A (S. cerevisiae)	SI00305277	Hs_ASF1A_1
		**CTGTAGAAAGTTTATAAGAAA**		SI00305291	Hs_ASF1A_3
		**AAGGCATATGTTTGTATTTCA**		SI00305284	Hs_ASF1A_2
		**AACCATGGATTTAGAAAGGTA**		SI00305277	Hs_ASF1A_1
**ASF1B**	NM_018154	**TTGCTGTTTGTATATTATCAA**	ASF1 anti-silencing function 1 homolog B (S. cerevisiae)	SI00305312	Hs_ASF1B_2
		**CCGAGTGGGCTACTACGTCAA**		SI00305319	Hs_ASF1B_3
		**AAGCAGGTACTTCAGTTCTAA**		SI00305305	Hs_ASF1B_1
**ASH1L**	NM_018489	**CAGGCTGTCGTATCAATGCAA**	ash1 (absent, small, or homeotic)-like (Drosophila)	SI00122094	Hs_ASH1L_2
		**ACGGTAGTTCTTGCAATTATT**		SI00122101	Hs_ASH1L_3
		**TCGACTGTTATCTCACATTAA**		SI00122087	Hs_ASH1L_1
**ATAD2**	NM_014109	**AAGGCATTTATAAAGATCGAA**	ATPase family, AAA domain containing 2	SI00103138	Hs_ATAD2_3
		**AAGAATAATTAGCAGCGTTAA**		SI00103145	Hs_ATAD2_4
		**CTGGTACTTAGGCACCATAAA**		SI00103131	Hs_ATAD2_2
**ATF2**	NM_001880	**TCCATTTGAGAATGAATTCAA**	activating transcription factor 2	SI00305872	Hs_ATF2_2
		**CCGAGTAGTCCACATACAGAA**		SI00305879	Hs_ATF2_3
		**CGCGGGTGACCGAAAGGATCA**		SI02780309	Hs_ATF2_5
**BAP1**	NM_004656	**CTCAATTCCTCTGTCCATCAA**	BRCA1 associated protein-1 (ubiquitin carboxy-terminal hydrolase)	SI00066703	Hs_BAP1_2
		**CCCGCTGGTGCTGGAAGCAAA**		SI00066710	Hs_BAP1_3
		**CAGCAGCTGATAAGAGTAACA**		SI00066696	Hs_BAP1_1
**BARD1**	NM_000465	**CTGAATATTATACCAGATGAA**	BRCA1 associated RING domain 1	SI02664354	Hs_BARD1_7
		**AACTGGATGTCCAGTGTGTTA**		SI00299383	Hs_BARD1_5
		**TCCGATGAATTCATTAGTCTT**		SI00010136	Hs_BARD1_2
**BAZ1A**	NM_013448 NM_182648	**AACGAGGAAGACCACAAGTTA**	bromodomain adjacent to zinc finger domain, 1A	SI00102340	Hs_BAZ1A_1
		**CACAAGTTAGATTGCCAGTTA**		SI00102354	Hs_BAZ1A_3
		**CAGCTAGACCAGCTTATTGAA**		SI00102347	Hs_BAZ1A_2
**BAZ1B**	NM_023005 NM_032408	**AGGAGATAGTTCGATACTTTA**	bromodomain adjacent to zinc finger domain, 1B	SI00134960	Hs_BAZ1B_3
		**AAGAACGAATACGGAAGCACA**		SI03030909	Hs_BAZ1B_5
		**CAGATGCTCAGTATCCTATTA**		SI00134967	Hs_BAZ1B_4
**BAZ2A**	NM_013449	**CTCGGTAGCTTTGAATCTTAA**	bromodomain adjacent to zinc finger domain, 2A	SI00102368	Hs_BAZ2A_1
		**CCCGCAGTGACGGCCAGTAAA**		SI00102382	Hs_BAZ2A_3
		**CACCACTACAGAGATATCATA**		SI00102375	Hs_BAZ2A_2
**BAZ2B**	NM_013450	**AGGGCCTAAATTAAAGTATAA**	bromodomain adjacent to zinc finger domain, 2B	SI00102396	Hs_BAZ2B_1
		**TTGGAGCGGAACATTGAAAGA**		SI03024700	Hs_BAZ2B_5
		**CTGAAGGTAATGATAGCTATA**		SI00102403	Hs_BAZ2B_2
**BCCIP**	NM_016567	**GCACCTAGTAATGGTAAATTA**	BRCA2 and CDKN1A interacting protein	SI00116858	Hs_BCCIP_2
		**CAGTGTGTTGAACAAATTCAA**		SI02654232	Hs_BCCIP_8
		**AAGGCTGGACTAATTCAATCA**		SI00116851	Hs_BCCIP_1
**BCL10**	NM_003921	**TTGAATCTATTCGGCGAGAAA**	B-cell CLL/lymphoma 10	SI03023559	Hs_BCL10_6
		**CACGTACTGTTTCACGACAAT**		SI00057778	Hs_BCL10_4
		**CAGAAGGAGAATCCAGCACGA**		SI03063144	Hs_BCL10_7
**BMI1**	NM_005180	**TTGGATCGGAAAGTAAACAAA**	BMI1 polycomb ring finger oncogene	SI00073311	Hs_PCGF4_1
		**ATGGGTCATCAGCAACTTCTT**		SI03051874	Hs_BMI1_1
		**CAGAGTTCGACCTACTTGTAA**		SI00073325	Hs_PCGF4_3
**BPTF**	NM_004459 NM_182641	**AAGGTCCAACTTGCAGAATTA**	bromodomain PHD finger transcription factor	SI00064015	Hs_FALZ_2
		**AACGCCTTATGATGAATCTAA**		SI00064022	Hs_FALZ_3
		**AAGCATAATGCTGTAATAGAA**		SI00064008	Hs_FALZ_1
**BRAP**	NM_006768	**CCCACTGCGCAAAGAAGTAAA**	BRCA1 associated protein	SI00091735	Hs_BRAP_2
		**CAGGAGAGAAAGTAGCGATTA**		SI00091749	Hs_BRAP_4
		**CAAGATAGTTCGGATAGAGAA**		SI00091728	Hs_BRAP_1
**BRCA1**	NM_007294 NM_007295 NM_007296 NM_007297 NM_007299 NM_007300	**CAGCAGTTTATTACTCACTAA**	breast cancer 1, early onset	SI02654575	Hs_BRCA1_13
		**AACCTATCGGAAGAAGGCAAG**		SI00299495	Hs_BRCA1_9
		**ACCATACAGCTTCATAAATAA**		SI02664368	Hs_BRCA1_15
**BRCA2**	NM_000059	**TTGGAGGAATATCGTAGGTAA**	breast cancer 2, early onset	SI02653595	Hs_BRCA2_7
		**CAGGACACAATTACAACTAAA**		SI02653434	Hs_BRCA2_6
		**TTGGAGGAATATCGTAGGTAA**		SI02653595	Hs_BRCA2_7
		**CAGGACACAATTACAACTAAA**		SI02653434	Hs_BRCA2_6
		**CAGCGTTTGTGTATCGGGCAA**		SI00000966	Hs_BRCA2_1
		**CAGCGTTTGTGTATCGGGCAA**		SI00000966	Hs_BRCA2_1
**BRD1**	NM_014577	**TCCTGTCTGCTTAAGAACTAA**	bromodomain containing 1	SI00106960	Hs_BRD1_2
		**AAGACGGGATGTAAATTTACA**		SI00106967	Hs_BRD1_3
		**CTGACCTACGCTCAAGCTCAA**		SI00106953	Hs_BRD1_1
**BRD2**	NM_005104	**CCGGAGGTGTCCAATCCCAAA**	bromodomain containing 2	SI00313236	Hs_BRD2_2
		**CACCTTGGAAATGAAGTTTAT**		SI00313243	Hs_BRD2_3
		**CAGGAGTTTGCTGCTGATGTA**		SI00313229	Hs_BRD2_1
**BRD3**	NM_007371	**CTCGAATTGCTACAAATACAA**	bromodomain containing 3	SI00313264	Hs_BRD3_2
		**CTGCTTCTTAATTCAGTGTTA**		SI00313271	Hs_BRD3_3
		**CAGGGAGATGCTATCCAAGAA**		SI00313257	Hs_BRD3_1
**BRD4**	NM_014299 NM_058243	**CAAGCTGAACCTCCCTGATTA**	bromodomain containing 4	SI03157427	Hs_BRD4_5
		**CCGGCCTGTGAAACCTCCAAA**		SI03190845	Hs_BRD4_6
		**AACCGAGATCATGATAGTCCA**		SI00313306	Hs_BRD4_4
**BRD7**	NM_013263	**ATGCTTGAAGATAAGTTTAAA**	bromodomain containing 7	SI00313327	Hs_BRD7_3
		**AGCGCTGAAAGCAGTAACAAA**		SI00313334	Hs_BRD7_4
		**CTGGTATTTGATTATATATTA**		SI00313313	Hs_BRD7_1
**BRD8**	NM_006696 NM_139199 NM_183359	**CCGGCTGTCTCCACTACTGAA**	bromodomain containing 8	SI00091154	Hs_BRD8_4
		**AAGGCGATGAGACTCCACTTA**		SI03035424	Hs_BRD8_12
		**AAGGTAGATGATCATCCTGAA**		SI00091147	Hs_BRD8_3
**BRIP1**	NM_032043	**AAGATAAACAGTCCACTTCAA**	BRCA1 interacting protein C-terminal helicase 1	SI00141988	Hs_BRIP1_2
		**ACAGTCAAGAGTCATCGAATA**		SI03037993	Hs_BRIP1_5
		**TAGCATGGCAACAATCTCTTA**		SI00141981	Hs_BRIP1_1
**BRMS1**	NM_001024957 NM_001024958 NM_015399	**CAGGAATAAGTACGAATGTGA**	breast cancer metastasis suppressor 1	SI00110796	Hs_BRMS1_1
		**AAGGAGAAGTTGTTCAGGGAA**		SI00110803	Hs_BRMS1_2
		**CTGTCTGGATGTGATCAGGAA**		SI00110817	Hs_BRMS1_4
**BRMS1L**	NM_032352	**ATGCAAATTCGTACAAAGGTA**	breast cancer metastasis-suppressor 1-like	SI02664669	Hs_BRMS1L_6
		**CCGCTGGCAACTTTACAGGAA**		SI02664676	Hs_BRMS1L_7
		**CACAGCATTGATATTACCTCA**		SI00142800	Hs_BRMS1L_2
**BRPF1**	NM_001003694 NM_004634	**AAGGGAGACGATCAAGGTTCA**	bromodomain and PHD finger containing, 1	SI00066339	Hs_BRPF1_3
		**TCCCAAGTCAGGCAAACATAA**		SI00066346	Hs_BRPF1_4
		**CCGAAAGGTCTACAAGAGTTA**		SI00066332	Hs_BRPF1_2
**BRPF3**	NM_015695	**CCAGATTACCTGGAATTCATA**	bromodomain and PHD finger containing, 3	SI00313565	Hs_BRPF3_1
		**CCAGGATATCACCGAATGCAA**		SI00313572	Hs_BRPF3_2
		**CCGGCTTGAGAAAGAGTCATA**		SI00313579	Hs_BRPF3_3

**BRWD1**	NM_001007246 NM_018963 NM_033656	**CTGGGAGGGCAACGAGCACAA**	bromodomain and WD repeat domain containing 1	SI00762734	Hs_WDR9_2
		**AGGAGTTGGTCTTGTCCAATA**		SI00762748	Hs_WDR9_4
		**CACCCAGTATTTCAAGAGTCA**		SI00762727	Hs_WDR9_1
**C14orf43**	NM_001043318 NM_194278	**CAGAAAGTATTCATACTGCTA**	chromosome 14 open reading frame 43	SI00319991	Hs_C14orf43_3
		**CCCGAGTGCATTTATCCCAAA**		SI00319977	Hs_C14orf43_1
		**CTGGCATAGGTATATCCTTAA**		SI00319984	Hs_C14orf43_2
**C1D**	NM_006333 NM_173177	**CAGCTGTGAAATTTAAGTGAA**	nuclear DNA-binding protein	SI02637250	Hs_C1D_5
		**CACAGTAAATATGTAAAGCTA**		SI00087549	Hs_C1D_1
		**CTCATTAACTTTAGATGGGAA**		SI02637257	Hs_C1D_6
**C20orf191**	NM_001039379 NR_003678 XM_086725	**ATAGAGTGTATGGATCGTGTA**	chromosome 20 open reading frame 191	SI03046575	Hs_C20orf191_1
		**TTCGTCTAATATATTCTAAGA**		SI00480620	Hs_LOC149934_1
		**CAGCAGGATCCAGCATTTGGA**		SI03065741	Hs_C20orf191_2
**C6orf130**	NM_145063	**ACCGGGCAAACCTACCTTAAA**	chromosome 6 open reading frame 130	SI00330365	Hs_C6orf130_1
		**AAGGAGTTAGGTGGCTTGATA**		SI00330372	Hs_C6orf130_2
		**AAGGGTGACTTGGCTGAAGAA**		SI00330379	Hs_C6orf130_3
**CARM1**	NM_199141	**CAGGATAGAAATCCCATTCAA**	coactivator-associated arginine methyltransferase 1	SI02663815	Hs_CARM1_5
		**CACAACAACCTGATTCCTTTA**		SI02663822	Hs_CARM1_6
		**AGGGTTCACCTCACACTTGAA**		SI00164486	Hs_CARM1_3
**CARM1L**	XM_171224	**AACAGTCAAATACACAGCAAA**	coactivator associated arginine methyltransferase 1-like	SI00338492	Hs_CARM1L_2
		**CAGAATCACATTGACTTTAGA**		SI00338485	Hs_CARM1L_1
		**AAGGTGACTGATGGTGAATTA**		SI00338499	Hs_CARM1L_3
**CBX1**	NM_006807	**AAGGGAAGGAGTTCTACTTGT**	chromobox homolog 1 (HP1 beta homolog Drosophila)	SI00299670	Hs_CBX1_1
		**TTGGCTATTACTGGTGTATTA**		SI02657249	Hs_CBX1_3
		**CAGGAGAAGATTAAACTTTCA**		SI02657242	Hs_CBX1_2
**CBX2**	NM_005189 NM_032647	**CAGCCGGCGCTCCAAGCTCAA**	chromobox homolog 2 (Pc class homolog, Drosophila)	SI03171203	Hs_CBX2_1
		**CGCCGAGTGCATCCTGAGCAA**		SI03193890	Hs_CBX2_2
**CBX3**	NM_007276 NM_016587	**CTGGTTACTTTGAACAAATAA**	chromobox homolog 3 (HP1 gamma homolog, Drosophila)	SI02665222	Hs_CBX3_6
		**TCCCTTCAAGTTAAATTGAAA**		SI02638650	Hs_CBX3_5
		**AAAGTACTAGATCGACGTGTA**		SI00096173	Hs_CBX3_2
**CBX4**	NM_003655	**CTGCCTCACCGTTACTTTCAA**	chromobox homolog 4 (Pc class homolog, Drosophila)	SI00054257	Hs_CBX4_1
		**CAGCCCGAGGTCATCCTGCTA**		SI00054271	Hs_CBX4_3
		**GAGGCTGGTCGCCCAAATATA**		SI00054264	Hs_CBX4_2
**CBX5**	NM_012117	**CAGGGAGAAGTCAGAAAGTAA**	chromobox homolog 5 (HP1 alpha homolog, Drosophila)	SI00339157	Hs_CBX5_1
		**CTGAGCTAATTTCTGAATTTA**		SI00339171	Hs_CBX5_3
		**GCCGATGACATCAAATCTAAA**		SI00339164	Hs_CBX5_2
**CBX6**	NM_014292	**CCGCATCATCCTGAACCTGAA**	chromobox homolog 6	SI00339185	Hs_CBX6_1
		**CGCGGCCGAATCCATCATCAA**		SI00339199	Hs_CBX6_3
		**CCGCGTTATAGGCAAGAGCAA**		SI00339192	Hs_CBX6_2
**CBX7**	NM_175709	**TCGAGCTTTGTTCAGACTAAA**	chromobox homolog 7	SI00339227	Hs_CBX7_3
		**CCGCCTCGTCATGGCCTACGA**		SI03189137	Hs_CBX7_6
		**CACGAGTTGATTCCAAGCACA**		SI00339234	Hs_CBX7_4
**CBX8**	NM_020649	**AAGATTATCTCTAGAGTTATA**	chromobox homolog 8 (Pc class homolog, Drosophila)	SI00339241	Hs_CBX8_1
		**AAGGAAAGTAACACGGACCAA**		SI00339255	Hs_CBX8_3
		**ACGCATGGAATACCTCGTGAA**		SI00339248	Hs_CBX8_2
**CDY1**	NM_004680 NM_170723	**AAGAAAGCTTTCTGTACTACA**	chromodomain protein, Y-linked, 1	SI03127355	Hs_CDY1_5
		**AGCACTTAAGGAATAACAGAA**		SI03143140	Hs_CDY1_6
		**AGCACTTAAGGAATAACAGAA**		SI03143140	Hs_CDY1_6
		**TAGCTGCTATTTCGCACAATT**		SI03227938	Hs_CDY1_7
		**TAGCTGCTATTTCGCACAATT**		SI03227938	Hs_CDY1_7
		**AAGAAAGCTTTCTGTACTACA**		SI03127355	Hs_CDY1_5
**CDY1B**	NM_001003894 NM_001003895	**AGCACTTAAGGAATAACAGAA**	chromodomain protein, Y-linked, 1B	SI03143133	Hs_CDY1B_6
		**TAGCTGCTATTTCGCACAATT**		SI03227924	Hs_CDY1B_7
		**AAGAAAGCTTTCTGTACTACA**		SI03127341	Hs_CDY1B_5
**CDY2A**	NM_004825	**AAGAAAGCTTTCTGTACTACA**	chromodomain protein, Y-linked, 2A	SI03127348	Hs_CDY2A_1
		**TAGCTGCTATTTCGCACAATT**		SI03227931	Hs_CDY2A_3
		**TACACCAGTGGTGACCAAATA**		SI03223808	Hs_CDY2A_2
**CDYL**	NM_004824 NM_170751 NM_170752	**CTACCTTATATTCAACATTTA**	chromodomain protein, Y-like	SI00342636	Hs_CDYL_2
		**CAGAGATATTGTGGTCAGGAA**		SI00342650	Hs_CDYL_4
		**CACAAAGTCCTCAGAGAATAA**		SI00342643	Hs_CDYL_3
**CDYL2**	NM_152342	**AAGGACTACGTCTTTGACAAA**	chromodomain protein, Y-like 2	SI00342664	Hs_CDYL2_2
		**ACCCAATTGAGTGATTTGCAA**		SI00342671	Hs_CDYL2_3
		**AACCCAGATATTGTACATTTA**		SI00342657	Hs_CDYL2_1
**CECR2**	NM_031413 XM_926014 XM_938600	**AACCATGGTGCTACGAACCAA**	cat eye syndrome chromosome region, candidate 2	SI02822722	Hs_CECR2_6
		**ATCGATCGTACAAGTACCTGA**		SI02822729	Hs_CECR2_7
		**ATCGATCGTACAAGTACCTGA**		SI02822729	Hs_CECR2_7
		**ATGGAGGTTTATACTGTACCA**		SI02822736	Hs_CECR2_8
		**ATGGAGGTTTATACTGTACCA**		SI02822736	Hs_CECR2_8
		**AACCATGGTGCTACGAACCAA**		SI02822722	Hs_CECR2_6
**CENPA**	NM_001042426 NM_001809	**CAGAGGTAATATATGAGACAA**	centromere protein A	SI00343161	Hs_CENPA_1
		**CCGAGTTACTCTCTTCCCAAA**		SI00343168	Hs_CENPA_2
		**TAGCTCTTTCTGTAATATTTA**		SI00343175	Hs_CENPA_3
**CENPB**	NM_001810	**CGCCTTGAGAAGCACAGTTTA**	centromere protein B, 80kDa	SI00028644	Hs_CENPB_1
		**CCGGGAGAAGTCACGGATCAT**		SI00028651	Hs_CENPB_2
		**CACGCTGAGCACGATCCTGAA**		SI00028658	Hs_CENPB_3
**CHAF1A**	NM_005483	**AAGGAAGAAGAGAAACGGTTA**	chromatin assembly factor 1, subunit A (p150)	SI00345184	Hs_CHAF1A_2
		**CAGCCATGGATTGCAAAGATA**		SI00345198	Hs_CHAF1A_4
		**CTGCCCTTTAATAAAGCATTA**		SI00345191	Hs_CHAF1A_3
**CHAF1B**	NM_005441	**ACGGAAAGTCTGGACCCTTGA**	chromatin assembly factor 1, subunit B (p60)	SI02652944	Hs_CHAF1B_6
		**GGGCAACTGATGGGAATTTAA**		SI00077224	Hs_CHAF1B_1
		**CACCAATGTCAGGATCTGGAA**		SI02653539	Hs_CHAF1B_7
**CHD1L**	NM_004284	**CTGGTGGGAATCCAACAATTA**	chromodomain helicase DNA binding protein 1-like	SI00345485	Hs_CHD1L_1
		**ATGTACTTATTTGAAGGTAAA**		SI00345499	Hs_CHD1L_3
		**CAGATGTTGGATATTCTCCAA**		SI00345492	Hs_CHD1L_2
**CHD2**	NM_001042572 NM_001271	**TCGCGATTTAATATTAAGGAA**	chromodomain helicase DNA binding protein 2	SI00345520	Hs_CHD2_2
		**CAGTCGGAATCTGAGAGCGAA**		SI03178343	Hs_CHD2_6
		**CAAGAGGAGGACAGTTCGCTA**		SI03156944	Hs_CHD2_5
**CHD4**	NM_001273	**CATGAAGGTTATAAATACGAA**	chromodomain helicase DNA binding protein 4	SI00024549	Hs_CHD4_2
		**CGGGTATTGAATGGTTACTCA**		SI00024563	Hs_CHD4_4
		**TAAGTTCTTCCGGGTATTGAA**		SI00024556	Hs_CHD4_3
**CHD5**	NM_015557	**CCCACGGTACATGATCCTCAA**	chromodomain helicase DNA binding protein 5	SI00345569	Hs_CHD5_1
		**CTGGGCTACATGGATGAGAAA**		SI00345583	Hs_CHD5_3
		**CAGCTACAAGATTGATTACAA**		SI00345576	Hs_CHD5_2
**CHD7**	NM_017780	**CCCTAATAAATTGGATATAAA**	chromodomain helicase DNA binding protein 7	SI00345639	Hs_CHD7_3
		**CAGGGATTAGTTAACAATACA**		SI00345625	Hs_CHD7_1
		**TACCATTGGTATTATAATAAA**		SI00345632	Hs_CHD7_2
**CHD8**	NM_020920	**AAGGATAAGAGGATACATCAA**	chromodomain helicase DNA binding protein 8	SI00345660	Hs_CHD8_2
		**CAGATTGGATCCGGAAATATA**		SI00345667	Hs_CHD8_3
		**CCGGATCCCTGTCATCAATAA**		SI03190488	Hs_CHD8_5
**CHRAC1**	NM_017444	**TCGGTTATGTAAATTCATATA**	chromatin accessibility complex 1	SI00346269	Hs_CHRAC1_1
		**CTGTGTTTCAATAGAGCTTTA**		SI00346283	Hs_CHRAC1_3
		**AAGTCAAATTATCAAATCAAA**		SI00346276	Hs_CHRAC1_2
**CIITA**	NM_000246	**CTGGCTGGGATTCCTACACAA**	class II, major histocompatibility complex, transactivator	SI00645470	Hs_MHC2TA_2
		**CTCTGGTTATCTAATATGTAA**		SI00645484	Hs_MHC2TA_4
		**CAGGCTAAGCTTGTACAATAA**		SI00645463	Hs_MHC2TA_1
**CREBBP**	NM_001079846 NM_004380	**CCCGCATTGTCGAACCATGAA**	CREB binding protein (Rubinstein-Taybi syndrome)	SI02622648	Hs_CREBBP_2
		**AACGGAGGTCGCGTTTACATA**		SI02633085	Hs_CREBBP_6
		**CCCGATCCTGGTGAATCCAAA**		SI02633092	Hs_CREBBP_7
**CSDC2**	NM_014460	**CACGTCAAACTCTCTTCCCAA**	cold shock domain containing C2, RNA binding	SI00685090	Hs_PIPPIN_2
		**CAGGAGGTAGGTGGAGGGCAA**		SI00685083	Hs_PIPPIN_1
		**CTCCTTTACCCTGACTCACAA**		SI00685097	Hs_PIPPIN_3
**DICER1**	NM_030621 NM_177438	**AATGTGCTATCTGGATCCTAG**	Dicer1, Dcr-1 homolog (Drosophila)	SI00300006	Hs_DICER1_1
		**ACCACTGATTCTGCATATGAA**		SI02655492	Hs_DICER1_11
		**ATCGATCCTATGTTCAATCTA**		SI02645972	Hs_DICER1_3
**DMAP1**	NM_001034023 NM_001034024 NM_019100	**CAGCTTTATCTCCACGATGAT**	DNA methyltransferase 1 associated protein 1	SI00369964	Hs_DMAP1_2
		**GCGGATGTACGGGACATTCTA**		SI00369957	Hs_DMAP1_1
		**CCGGCTGTTCCTGAGACTGCA**		SI00369971	Hs_DMAP1_3
**DNAJC1**	NM_022365	**AAGAAACAAGATTGAAGGAAA**	DnaJ (Hsp40) homolog, subfamily C, member 1	SI00370965	Hs_DNAJC1_1
		**CACCTTTAGAAACTACATATA**		SI00370972	Hs_DNAJC1_2
		**CTGACTAGAACTGAACTTGAA**		SI00370979	Hs_DNAJC1_3
**DNMT1**	NM_001379	**AACGGTGCTCATGCTTACAAC**	DNA (cytosine-5-)-methyltransferase 1	SI00300062	Hs_DNMT1_1
		**CCCAATGAGACTGACATCAAA**		SI02663409	Hs_DNMT1_6
		**CGGGAAGTGAATGGACGTCTA**		SI02663416	Hs_DNMT1_7
**DNMT3A**	NM_022552 NM_153759 NM_175629	**CAGAACAAGCCCATGATTGAA**	DNA (cytosine-5-)-methyltransferase 3 alpha	SI02665271	Hs_DNMT3A_10
		**CTCAGTGGTGTGTGTTGAGAA**		SI02665278	Hs_DNMT3A_11
		**CTCGCGATTTCTCGAGTCCAA**		SI03091396	Hs_DNMT3A_14
**DNMT3B**	NM_006892 NM_175848 NM_175849 NM_175850	**CTCACGGTTCCTGGAGTGTAA**	DNA (cytosine-5-)-methyltransferase 3 beta	SI00092974	Hs_DNMT3B_3
		**AAGGACTACTTTGCATGTGAA**		SI00092967	Hs_DNMT3B_2
		**ACCCAAGCGCCTCAAGACAAA**		SI03038952	Hs_DNMT3B_6
		**CTCACGGTTCCTGGAGTGTAA**		SI00092974	Hs_DNMT3B_3
		**ACCCAAGCGCCTCAAGACAAA**		SI03038952	Hs_DNMT3B_6
		**AAGGACTACTTTGCATGTGAA**		SI00092967	Hs_DNMT3B_2
**DNMT3L**	NM_013369 NM_175867	**AAGGCTAACCAGCGAAATATA**	DNA (cytosine-5-)-methyltransferase 3-like	SI00372281	Hs_DNMT3L_1
		**AAGCATGTGGTTGATGTCACA**		SI00372288	Hs_DNMT3L_2
		**CGCGGCCAAGTGGCCCACCAA**		SI03085180	Hs_DNMT3L_5

		**ACCGGTGGAAATGACAGGAAA**		SI00372316	Hs_DNTT_2
**DNTT**	NM_001017520 NM_004088	**CTGGGATTGGATTATATTGAA**	deoxynucleotidyltransferase, terminal	SI00372309	Hs_DNTT_1
		**AACCACTTTAAACAACTGTAA**		SI00372323	Hs_DNTT_3
		**CCGGCCTTCGTCGAAGCAGAA**		SI00143311	Hs_DOT1L_2
**DOT1L**	NM_032482	**CACACTATCGACCGCACCATA**	DOT1-like, histone H3 methyltransferase (S. cerevisiae)	SI00143325	Hs_DOT1L_4
		**CCGCTGCCGGTCTACGATAAA**		SI00143318	Hs_DOT1L_3
		**CAGCCGTATATGGAACATAAA**		SI00373485	Hs_DR1_1
**DR1**	NM_001938	**AAGATAGTATTAAGTAATTAA**	down-regulator of transcription 1, TBP-binding (negative cofactor 2)	SI00373492	Hs_DR1_2
		**CTGAGGTTTATGAATATTCAA**		SI00373499	Hs_DR1_3
		**AACGAAGAATGGGAACCTATA**		SI02778923	Hs_EHMT1_5
**EHMT1**	NM_024757	**CAACGGATACATCTTAAATAA**	euchromatic histone-lysine N-methyltransferase 1	SI00137074	Hs_EHMT1_3
		**CAGCAACGGATACATCTTAAA**		SI00137060	Hs_EHMT1_1
		**ATCGAGGTGATCCGCATGCTA**		SI00091189	Hs_BAT8_1
**EHMT2**	NM_006709 NM_025256	**AAGCTCTAACTGAACAACTAA**	euchromatic histone-lysine N-methyltransferase 2	SI00091210	Hs_BAT8_4
		**CACCATGAACATCGATCGCAA**		SI00091203	Hs_BAT8_3
		**CTCCAAGAATTGTGCAAGTAA**		SI00377440	Hs_EIF2C1_2
**EIF2C1**	NM_012199	**TAGGCTTAACATAAAGCCGAA**	eukaryotic translation initiation factor 2C, 1	SI00377433	Hs_EIF2C1_1
		**CAGCTACAACTTAGATCCCTA**		SI00377447	Hs_EIF2C1_3
		**ACAGATCCATACGTCCGTGAA**		SI00377468	Hs_EIF2C2_2
**EIF2C2**	NM_012154	**ATACGGGTCTGTGGTGATAAA**	eukaryotic translation initiation factor 2C, 2	SI00377461	Hs_EIF2C2_1
		**CAGCACCGGCAGGAGATCATA**		SI03065461	Hs_EIF2C2_5
		**CTCCTATAGGAAGTATCGCAA**		SI00377496	Hs_EIF2C3_2
**EIF2C3**	NM_024852 NM_177422	**CTGACTGATTCTCATCGGGTA**	eukaryotic translation initiation factor 2C, 3	SI00377489	Hs_EIF2C3_1
		**CGGGATGAAATGGCTCATGTA**		SI00377503	Hs_EIF2C3_3
		**AAGGCATGTATTAGCTTGGAA**		SI00377524	Hs_EIF2C4_2
**EIF2C4**	NM_017629	**TTGGACGGGATAGGGTTGATA**	eukaryotic translation initiation factor 2C, 4	SI00377517	Hs_EIF2C4_1
		**CCAGAGTTTACTGACTTGTAA**		SI00377531	Hs_EIF2C4_3
		**AAGGTAAGAGTTCAAGTTCAA**		SI00379008	Hs_ELP3_2
**ELP3**	NM_018091	**CAGGACATACTTCCAACAATA**	elongation protein 3 homolog (S. cerevisiae)	SI00379015	Hs_ELP3_3
		**CAGGATTTCTTAAATACTCAA**		SI00379001	Hs_ELP3_1
		**CACCGATAACTCAGACTTGAA**		SI02622592	Hs_EP300_3
**EP300**	NM_001429	**ACCAAGGGTTGCAAACGGAAA**	E1A binding protein p300	SI03038259	Hs_EP300_9
		**TTGGACTACCCTATCAAGTAA**		SI02626267	Hs_EP300_7
		**AACCATGTTTACAACTATCAA**		SI02665166	Hs_EZH2_7
**EZH2**	NM_004456 NM_152998	**CAGACGAGCTGATGAAGTAAA**	enhancer of zeste homolog 2 (Drosophila)	SI00063966	Hs_EZH2_3
		**AAGCAAATTCTCGGTGTCAAA**		SI00063959	Hs_EZH2_2
		**AGGAGTGGACTAGAAGTTTAA**		SI00143794	Hs_FBXL10_3
**FBXL10**	NM_001005366 NM_032590	**CAGATCTAACAAAGTAGGCTA**	F-box and leucine-rich repeat protein 10	SI00143801	Hs_FBXL10_4
		**CCGGCAAGACCGGGAAACAAA**		SI00143787	Hs_FBXL10_2
		**AAGCCAGACTGAATCAACAAA**		SI00099204	Hs_FBXL11_2
**FBXL11**	NM_012308	**CTCACTGGAGTTCCTATAGTA**	F-box and leucine-rich repeat protein 11	SI00099218	Hs_FBXL11_4
		**ACCCATTTCGTTGCTACCCAA**		SI00099197	Hs_FBXL11_1
		**CAGGATTAGAACCACTTTCAA**		SI00422940	Hs_FYCO1_1
**FYCO1**	NM_024513	**CAGGAGAGACTGAATTAATAA**	FYVE and coiled-coil domain containing 1	SI00422954	Hs_FYCO1_3
		**AAGCCACGTCATATAACTCAA**		SI00422961	Hs_FYCO1_4
		**CAGTTAGGGACTTTAATTTAA**		SI00676606	Hs_p66alpha_2
**GATAD2A**	NM_017660	**CTGCTACAACATTCTAATAAA**	GATA zinc finger domain containing 2A	SI00676599	Hs_p66alpha_1
		**CCTCATGATGATGGAGTTAAA**		SI00676613	Hs_p66alpha_3
		**CTCCATTTGAGAAACCTAATA**		SI00426118	Hs_GCN5L2_3
**GCN5L2**	NM_021078	**CCGCGGCATCATCGAGTTCCA**	GCN5 general control of amino-acid synthesis 5-like 2 (yeast)	SI00426125	Hs_GCN5L2_4
		**TCCCTTAGAGGGAATAATAAA**		SI00426104	Hs_GCN5L2_1
		**CAGGTCAAGCATAACAACGAA**		SI00432320	Hs_GTF3C4_1
**GTF3C4**	NM_012204	**AAAGATAAACATATCCCTCAA**	general transcription factor IIIC, polypeptide 4, 90kDa	SI00432334	Hs_GTF3C4_3
		**CAGGTAGATTTAATAGACCTA**		SI00432327	Hs_GTF3C4_2
		**CCCGTTGTGAGAGTTTCAGAA**		SI00075754	Hs_H1F0_1
**H1F0**	NM_005318	**CAAGAAGGTAGCCACGCCAAA**	H1 histone family, member 0	SI00075761	Hs_H1F0_2
		**CAAGCCGGTCAAGGCATCCAA**		SI03054177	Hs_H1F0_5
		**CAGCAGGAAGGCAAAGGCCAA**		SI00432943	Hs_H1FOO_2
**H1FOO**	NM_153833	**AAGCTGAGAGTAAGAGTTCAA**	H1 histone family, member O, oocyte-specific	SI00432950	Hs_H1FOO_3
		**CACGTCGGTGGCAGCTATCAA**		SI00432957	Hs_H1FOO_4
		**CAACGGTTCCTTCAAGCTCAA**		SI00432964	Hs_H1FX_1
**H1FX**	NM_006026	**TGGGCACAATAAAGCGTTTAA**	H1 histone family, member X	SI00432971	Hs_H1FX_2
		**CAAGATCTACACCGAGGCCAA**		SI00432978	Hs_H1FX_3
		**GTGGTTCACAACGACAGGCTA**		SI04133962	Hs_H2AFB3_5
**H2AFB3**	NM_080720 XM_373381	**CCAGAACAGCGGAGAGCGGAA**	H2A histone family, member B3	SI04273332	Hs_H2AFB3_6
		**CAACACGACCACCATCTCTCA**		SI04280626	Hs_H2AFB3_7
		**CGCGGGAAACAGGGCGGCAAA**		SI00433020	Hs_H2AFJ_1
**H2AFJ**	NM_018267 NM_177925	**CAACAAGAAGACCAGGATAAT**	H2A histone family, member J	SI00433034	Hs_H2AFJ_3
		**CAGACTGCTGCGCAAAGGGAA**		SI00433027	Hs_H2AFJ_2
**H2AFV**	NM_012412 NM_201436 NM_201516	**TTGTATGTTCTTAGACTCGAA**	H2A histone family, member V	SI03025792	Hs_H2AFV_10
		**CCGGGACGAAGCACTTGGTAA**		SI00032844	Hs_H2AFX_1
**H2AFX**	NM_002105	**CACGACTAGAACCTTAGGCAT**	H2A histone family, member X	SI02627786	Hs_H2AFX_3
		**TGCCTCCTAGGAGGACATTTA**		SI02627793	Hs_H2AFX_4
		**AAGGCTTTGGTTTCCAGTTTA**		SI00433048	Hs_H2AFY_1
**H2AFY**	NM_001040158 NM_004893 NM_138609 NM_138610	**CTGGCTGTGGCCAATGATGAA**	H2A histone family, member Y	SI00433055	Hs_H2AFY_2
		**CAAGTTTGTGATCCACTGTAA**		SI00433062	Hs_H2AFY_3
		**CTCAAAGAAGATATAGGTAAA**		SI00433076	Hs_H2AFY2_1
**H2AFY2**	NM_018649	**CAGCTTGAAGAGACCATCAAA**	H2A histone family, member Y2	SI00433083	Hs_H2AFY2_2
		**AAGAAGGAACTTCAAATTCCA**		SI00433090	Hs_H2AFY2_3
		**CTCAGGACTCTAAATACTCTA**		SI02654288	Hs_H2AFZ_7
**H2AFZ**	NM_002106	**AAGGTCATATCCCAAACTAAA**	H2A histone family, member Z	SI02653301	Hs_H2AFZ_6
		**GCCGTATTCATCGACACCTAA**		SI00032872	Hs_H2AFZ_3
		**TGGGAAGAAAGGACAACAGAA**		SI00505918	Hs_LOC346990_3
**H2AFZP2**	XM_294468	**CCAGTTGGGCGTATTCATCTA**	H2A histone family, member Z, pseudogene 2	SI00505904	Hs_LOC346990_1
		**AAAGCGTAGTACCACTGGTCA**		SI00505911	Hs_LOC346990_2
		**CACCAAGTACACCAGCGCTAA**		SI00433132	Hs_H2BFS_1
**H2BFS**	NM_017445	**CGCGCCCAAGAAGGGCTCGAA**	H2B histone family, member S	SI00433139	Hs_H2BFS_2
		**CTCCTCTAAGGCCATGGGAAT**		SI00433146	Hs_H2BFS_3
		**CAACTTTATAATAATTATAAA**		SI00433216	Hs_H3F3A_1
**H3F3A**	NM_002107	**AACGTGCTTAAGAATCCACTA**	H3 histone, family 3A	SI00433223	Hs_H3F3A_2
		**CTCCGTGAAATTAGACGTTAT**		SI00433230	Hs_H3F3A_3
		**CACCAGCATCATCTTAACTAA**		SI00433244	Hs_H3F3B_1
**H3F3B**	NM_005324	**CAGGTGTTGTTTGGTGGATAA**	H3 histone, family 3B (H3.3B)	SI00433251	Hs_H3F3B_2
		**CAGGTGTAGAACTCAGTCTTA**		SI00433258	Hs_H3F3B_3
		**TACCGGCGTGTTATTGAACGA**		SI00433916	Hs_HAT1_1
**HAT1**	NM_001033085 NM_003642	**CACGCTAGAAGGGTTTATGAA**	histone acetyltransferase 1	SI00433937	Hs_HAT1_4
		**AAGACTAATTAGCCCATATAA**		SI00433930	Hs_HAT1_3
		**CTCCGTGAAATTAGACATTAT**		SI02820020	Hs_LOC347376_6
**hCG_1749005**	NR_003933 XM_293312 XM_937928	**AACGTGTTTCAGAATACACCA**	H3 histone, family 3A	SI00506044	Hs_LOC347376_1
		**CGCAAATCTACCGGTGGTATA**		SI02820013	Hs_LOC347376_5
		**CACCCGGAGGAAAGTCTGTTA**		SI02663472	Hs_HDAC1_6
**HDAC1**	NM_004964	**CCACAGCGATGACTACATTAA**	histone deacetylase 1	SI00070623	Hs_HDAC1_3
		**CACAGCGATGACTACATTAAA**		SI00070609	Hs_HDAC1_1
		**CAGGTGAACAGTGGTATAGCA**		SI00141736	Hs_HDAC10_1
**HDAC10**	NM_032019	**CCGTTAGTAAACATCGCTCAA**	histone deacetylase 10	SI00141757	Hs_HDAC10_4
		**CGCCCTTGCGCCGTTAGTAAA**		SI00141743	Hs_HDAC10_2
		**AAGCAAGGTCTGGACCTGCAA**		SI00137473	Hs_HDAC11_4
**HDAC11**	NM_024827	**CGCATCATTGCTGACTCCATA**	histone deacetylase 11	SI03084158	Hs_HDAC11_6
		**ACCCAGCGTCTCCGCACAGAA**		SI03039085	Hs_HDAC11_5
		**ACGGTCAATAAGACCAGATAA**		SI00434952	Hs_HDAC2_1
**HDAC2**	NM_001527	**TCCCAATGAGTTGCCATATAA**	histone deacetylase 2	SI00434966	Hs_HDAC2_3
		**CTGGGTTGTTTCAATCTAACA**		SI00434959	Hs_HDAC2_2
		**CAGCCGGTTATCAACCAGGTA**		SI00057316	Hs_HDAC3_1
**HDAC3**	NM_003883	**GACCATGACAATGACAAGGAA**	histone deacetylase 3	SI00057337	Hs_HDAC3_4
		**CAGGTAGTGGACTTCTACCAA**		SI00057323	Hs_HDAC3_2
		**TTCCAATGTATTCCAAGCTAA**		SI00083951	Hs_HDAC4_3
**HDAC4**	NM_006037	**ACGGTTTATTCTGATTGAGAA**	histone deacetylase 4	SI02636536	Hs_HDAC4_5
		**TTGCGTCTTATTGAACTTATT**		SI00083958	Hs_HDAC4_4
		**CTACGACACGTTCATGCTAAA**		SI00077714	Hs_HDAC5_1
**HDAC5**	NM_001015053 NM_005474	**CACGCTAGAGAAAGTCATCGA**	histone deacetylase 5	SI03060330	Hs_HDAC5_5
		**GCCGGGTTTGATGCTGTTGAA**		SI00077735	Hs_HDAC5_4
		**CACCGTCAACGTGGCATGGAA**		SI02663808	Hs_HDAC6_5
**HDAC6**	NM_006044	**CCGCATTATCCTTATCCTAGA**	histone deacetylase 6	SI00084000	Hs_HDAC6_2
		**CACTTCGAAGCGAAATATTAA**		SI02757769	Hs_HDAC6_6
		**AAGGCTGGAAACAGAAACCCA**		SI02777719	Hs_HDAC7A_5
**HDAC7A**	NM_001098415 NM_001098416 NM_015401 NM_016596	**TCCACTTTGCCCAGTCCTTAA**	histone deacetylase 7A	SI00110845	Hs_HDAC7A_4
		**CAGCAAGATCCTCATTGTAGA**		SI02777726	Hs_HDAC7A_6
		**AGGGACGGTACTACAGTGTAA**		SI00122066	Hs_HDAC8_2
**HDAC8**	NM_018486	**ATGAGGATAGTTAAGCCTAAA**	histone deacetylase 8	SI03049382	Hs_HDAC8_5
		**ACGGAATGTGCAAAGTAGCAA**		SI00122080	Hs_HDAC8_4
		**CAGCAACGCATTCTAATTCAT**		SI00148372	Hs_HDAC9_1
**HDAC9**	NM_014707 NM_058176 NM_058177 NM_178423 NM_178425	**CCAACTGGAAGTGTTACTGAA**	histone deacetylase 9	SI00148393	Hs_HDAC9_4
		**TTGGCTCAGCTGGTCATTCAA**		SI03025071	Hs_HDAC9_5
		**CGGGATGATCATGAAGAGTTA**		SI00435659	Hs_HERC2_2

**HERC2**	NM_004667	**CCAGAGGATATTTAAACCAAA**	hect domain and RLD 2	SI00435666	Hs_HERC2_3
		**CAACGTTTGCTTATTAGTAAA**		SI00435673	Hs_HERC2_4
		**TTGTAGGAAATTTAAACTCTA**		SI00436555	Hs_HIRA_2
**HIRA**	NM_003325	**CAAACTGATTATGGTGTGGAA**	HIR histone cell cycle regulation defective homolog A (S. cerevisiae)	SI03155691	Hs_HIRA_5
		**CTAGAATTTGCTAACTTGTAA**		SI00436569	Hs_HIRA_4
		**CAGGTTTATACCACTTTATTT**		SI00436660	Hs_HIST1H1A_1
**HIST1H1A**	NM_005325	**ACCCAAGAAAGCGGCACCCAA**	histone cluster 1, H1a	SI00436667	Hs_HIST1H1A_2
		**AAGTTTCTTCTAGTAACCCAA**		SI00436674	Hs_HIST1H1A_3
		**AAGGCCGCTGCCAAACCGAAA**		SI00436688	Hs_HIST1H1B_1
**HIST1H1B**	NM_005322	**AACCTGCTCTTTAGATTTCGA**	histone cluster 1, H1b	SI00436695	Hs_HIST1H1B_2
		**AAGAAGGCAGGCGCCGCTAAA**		SI00436702	Hs_HIST1H1B_3
		**CCGAAGAAGAGCGCTAAGAAA**		SI00436716	Hs_HIST1H1C_1
**HIST1H1C**	NM_005319	**CGCGGCCACTGTAACCAAGAA**	histone cluster 1, H1c	SI00436723	Hs_HIST1H1C_2
		**AAGGTTGCGAAGCCCAAGAAA**		SI00436730	Hs_HIST1H1C_3
		**CTGACAGTTTGAGATTACTTA**		SI00436744	Hs_HIST1H1D_1
**HIST1H1D**	NM_005320	**ACCGCTGCTGGGACCAAGAAA**	histone cluster 1, H1d	SI00436751	Hs_HIST1H1D_2
		**AAAGACTCCTAAGAAGGTAAA**		SI00436758	Hs_HIST1H1D_3
		**CTGCTTAGAAGCCCAACACAA**		SI00436772	Hs_HIST1H1E_1
**HIST1H1E**	NM_005321	**AGCGAAGGCCAAAGCAGTTAA**	histone cluster 1, H1e	SI00436779	Hs_HIST1H1E_2
		**ACCCAAGGCGGCTAAACCAAA**		SI00436786	Hs_HIST1H1E_3
		**CCGCATCAAACTGTCCCTCAA**		SI00436800	Hs_HIST1H1T_1
**HIST1H1T**	NM_005323	**AGGCCACATCTAAGAAGTAAA**	histone cluster 1, H1t	SI00436807	Hs_HIST1H1T_2
		**ACCCAACATCATGAAGTTAAT**		SI00436814	Hs_HIST1H1T_3
		**AAGCCCAAAGCAAGTAACTTA**		SI00436828	Hs_HIST1H2AA_1
**HIST1H2AA**	NM_170745	**CTGCTTCGTAAGGGAAACTAT**	histone cluster 1, H2aa	SI00436835	Hs_HIST1H2AA_2
		**CAGGCAATGCGTCTCGCGATA**		SI00436842	Hs_HIST1H2AA_3
		**CAAGGGAAAGTGAAGAGTTAA**		SI00436856	Hs_HIST1H2AB_1
**HIST1H2AB**	NM_003513	**AATGACGAGGAGCTTAATAAA**	histone cluster 1, H2ab	SI00436863	Hs_HIST1H2AB_2
		**TCGCGGCAAACAAGGCGGTAA**		SI00436870	Hs_HIST1H2AB_3
		**CGGAAACGCTATCAAACCCAA**		SI00436884	Hs_HIST1H2AC_1
**HIST1H2AC**	NM_003512	**GGGCAAGTGATTTGACAGGTA**	histone cluster 1, H2ac	SI00436898	Hs_HIST1H2AC_3
		**CTCCGTAAAGGCAACTACGCA**		SI00436905	Hs_HIST1H2AC_4
		**CACAAGGCCAAGGGCAAGTAA**		SI00436933	Hs_HIST1H2AD_4
**HIST1H2AD**	NM_021065	**AAGGGCAACTACTCCGAGCGA**	histone cluster 1, H2ad	SI03133935	Hs_HIST1H2AD_5
		**ACGCGGCAAGCAAGGCGGAAA**		SI03140991	Hs_HIST1H2AD_6
		**GAGGAGCTAAATAAGCTTCTA**		SI00436940	Hs_HIST1H2AE_1
**HIST1H2AE**	NM_021052	**CAAGGGCAAGTGAAATGATTA**	histone cluster 1, H2ae	SI00436947	Hs_HIST1H2AE_2
		**ACGGCCGAGATCTTAGAGCTA**		SI00436954	Hs_HIST1H2AE_3
		**GTGGCCTAAATTCATAATAAA**		SI00436975	Hs_HIST1H2AG_2
**HIST1H2AG**	NM_021064	**GAGGATTATAATATGGTTCAA**	histone cluster 1, H2ag	SI00436982	Hs_HIST1H2AG_3
		**CACGATAACTCAGCAATTGTA**		SI00436989	Hs_HIST1H2AG_4
		**AGCCACCATAAGGCCAAATAA**		SI00436996	Hs_HIST1H2AH_1
**HIST1H2AH**	NM_080596	**CCGCAAGGGTAATTATGCCGA**	histone cluster 1, H2ah	SI00437003	Hs_HIST1H2AH_2
		**ATAAGGAGCGAGGTTGTGAAA**		SI00437010	Hs_HIST1H2AH_3
		**AAGGGCAAGTAGAAGCCTGGA**		SI00437031	Hs_HIST1H2AI_2
**HIST1H2AI**	NM_003509	**ACAGGGTGGCGTCCTGCCCAA**	histone cluster 1, H2ai	SI00437038	Hs_HIST1H2AI_3
		**GCCGGGCTTCAGTTTCCCGTA**		SI03220077	Hs_HIST1H2AI_5
		**GCCGGGCTTCAGTTTCCCGTA**		SI00437066	Hs_HIST1H2AJ_3
**HIST1H2AJ**	NM_021066	**CAACGCGGCCCGCGACAACAA**	histone cluster 1, H2aj	SI04261733	Hs_HIST1H2AJ_9
		**CCGCAACGATGAGGAGCTCAA**		SI04262223	Hs_HIST1H2AJ_10
		**CTGGAGCAGTTCATTACTCAT**		SI00437080	Hs_HIST1H2AK_1
**HIST1H2AK**	NM_003510	**AGGCTAAGACCCGTTCTTCAA**	histone cluster 1, H2ak	SI00437087	Hs_HIST1H2AK_2
		**TCATACCTCGGTCCAAACCAA**		SI00437101	Hs_HIST1H2AK_4
		**AAGGCAAATAATGTCTCCATA**		SI00437108	Hs_HIST1H2AL_1
**HIST1H2AL**	NM_003511	**CCATAGAATCACTTTCCAATA**	histone cluster 1, H2al	SI00437115	Hs_HIST1H2AL_2
		**CACAAGGCCAAAGGCAAATAA**		SI00437129	Hs_HIST1H2AL_4
		**CAAGTAAGGGCTGAACTTTAA**		SI00437136	Hs_HIST1H2AM_1
**HIST1H2AM**	NM_003514	**AAATGTAAACTTACAAGACAA**	histone cluster 1, H2am	SI00437143	Hs_HIST1H2AM_2
		**CTCCAATTTCCTGTAGGACGA**		SI00437150	Hs_HIST1H2AM_3
		**CTGGCATCTCTTCGAAAGCTA**		SI00437164	Hs_HIST1H2BA_1
**HIST1H2BA**	NM_170610	**CCCGTAAGGAGAGTTATTCTA**	histone cluster 1, H2ba	SI00437171	Hs_HIST1H2BA_2
		**CCAAGTAAGCCTGCTAAGTAA**		SI00437178	Hs_HIST1H2BA_3
		**AAGGAGGGACTTTCTCTGGAA**		SI00437451	Hs_HIST1H2BK_2
**HIST1H2BK**	NM_080593	**CTGTACCTTAATAAATTTGTA**	histone cluster 1, H2bk	SI00437458	Hs_HIST1H2BK_3
		**ACCAAGAAAGCTTCTTATCAA**		SI00437465	Hs_HIST1H2BK_4
		**CAAGCTCTTGTCCAACCCAAA**		SI00437472	Hs_HIST1H2BL_1
**HIST1H2BL**	NM_003519	**TTGCCACTATTGTTTCATTAT**	histone cluster 1, H2bl	SI00437479	Hs_HIST1H2BL_2
		**CCAGCTCCAAGTAAATTCTCA**		SI00437493	Hs_HIST1H2BL_4
		**CTCCAAGAAGGCCATTAACAA**		SI00437500	Hs_HIST1H2BM_1
**HIST1H2BM**	NM_003521	**CAAATCTGCTCCAGTCCCTAA**	histone cluster 1, H2bm	SI00437507	Hs_HIST1H2BM_2
		**AGCGTCACGCCTGGCGCATTA**		SI00437514	Hs_HIST1H2BM_3
		**CAAGGCCATGGGCATCATGAA**		SI00437535	Hs_HIST1H2BN_2
**HIST1H2BN**	NM_003520	**CACCAAGGCCGTCACCAAGTA**	histone cluster 1, H2bn	SI00437542	Hs_HIST1H2BN_3
		**CACCAAGTACACCAGTTCCAA**		SI00437549	Hs_HIST1H2BN_4
		**CGCCATGCCCGACCCGGCTAA**		SI03084361	Hs_HIST1H2BO_5
**HIST1H2BO**	NM_003527	**CTCGCAGCTGCCAGCAATCCA**	histone cluster 1, H2bo	SI03091298	Hs_HIST1H2BO_6
		**GAGTTACTCTATCTACGTGTA**		SI03104402	Hs_HIST1H2BO_7
		**ACTGAACTGCTTATTCGTAAA**		SI00437584	Hs_HIST1H3A_1
**HIST1H3A**	NM_003529	**CTCTCTGACGGTCCAAGCAAA**	histone cluster 1, H3a	SI00437591	Hs_HIST1H3A_2
		**TCGGAAGTCTACTGGTGGCAA**		SI00437598	Hs_HIST1H3A_3
		**ATGGCTCGTACTAAACAGACA**		SI00437619	Hs_HIST1H3B_2
**HIST1H3B**	NM_003537	**CAAGACTTCAAGACCGATCTT**	histone cluster 1, H3b	SI00437626	Hs_HIST1H3B_3
		**AGGGCTCTTTGAGGACACAAA**		SI00437633	Hs_HIST1H3B_4
		**CGCAAGCAGCTTGCTACTAAA**		SI00437640	Hs_HIST1H3C_1
**HIST1H3C**	NM_003531	**CCCGTTTCTTCCTCATTGAAA**	histone cluster 1, H3c	SI00437647	Hs_HIST1H3C_2
		**ATGGCTCGTACGAAGCAAACA**		SI00437654	Hs_HIST1H3C_3
		**ACGCGGCAAGCAAGGCGGAAA**		SI04215792	Hs_HIST1H3D_9
**HIST1H3D**	NM_003530	**AGGGCGTAAATTGTCTTGTGA**	histone cluster 1, H3d	SI00437668	Hs_HIST1H3D_1
		**ACCGAGCTGCTGATTCGCAAA**		SI03138989	Hs_HIST1H3D_5
		**AGGCGGTAAAGCACCGCGCAA**		SI00437710	Hs_HIST1H3E_3
**HIST1H3E**	NM_003532	**GAGTACAAACCTTAAATCCAA**	histone cluster 1, H3e	SI00437696	Hs_HIST1H3E_1
		**CAGGCGGTAAAGCACCGCGCA**		SI00437703	Hs_HIST1H3E_2
		**CTCCGTGAAATCCGCCGCTAT**		SI00437738	Hs_HIST1H3F_3
**HIST1H3F**	NM_021018	**GAGGGTGTGCGCCAACTTAAA**	histone cluster 1, H3f	SI00437724	Hs_HIST1H3F_1
		**GAGCTACTGATTCGCAAGCTA**		SI00437731	Hs_HIST1H3F_2
		**CAGTAATTCTATCCAACTTAA**		SI00437766	Hs_HIST1H3G_3
**HIST1H3G**	NM_003534	**CCGGGCAGTAATTCTATCCAA**	histone cluster 1, H3g	SI00437752	Hs_HIST1H3G_1
		**CAGATCTGCGCTTTCAGAGTT**		SI00437759	Hs_HIST1H3G_2
		**GCGAGAGGGCTTGAGTCTCAA**		SI00437794	Hs_HIST1H3H_3
**HIST1H3H**	NM_003536	**CTCACTGATTACATACCCAAA**	histone cluster 1, H3h	SI00437780	Hs_HIST1H3H_1
		**CCCATCGCTATCGGCCTGGTA**		SI00437787	Hs_HIST1H3H_2
		**AGCCATGGCACGAACAAAGCA**		SI00437822	Hs_HIST1H3I_3
**HIST1H3I**	NM_003533	**CAGCCCAAGAAGTCTGTCCAA**	histone cluster 1, H3i	SI00437808	Hs_HIST1H3I_1
		**AGGGCATAAATATTCCTATCA**		SI00437815	Hs_HIST1H3I_2
		**AGCGACTGGCGGTGTGAAGAA**		SI00437850	Hs_HIST1H3J_3
**HIST1H3J**	NM_003535	**ATCCGTGGCGAGCGAGCATAA**	histone cluster 1, H3j	SI00437836	Hs_HIST1H3J_1
		**CTGCTCTATCTTGGGTTTCTT**		SI00437843	Hs_HIST1H3J_2
		**ATCCGTGACGCTGTCACCTAT**		SI00437878	Hs_HIST1H4A_3
**HIST1H4A**	NM_003538	**CAGCTTGCATTTCTGAACCAA**	histone cluster 1, H4a	SI00437864	Hs_HIST1H4A_1
		**GCGGATCTCTGGTCTGATCTA**		SI00437871	Hs_HIST1H4A_2
		**ACGGCTTCGGCGGTTAATCTT**		SI00437906	Hs_HIST1H4B_3
**HIST1H4B**	NM_003544	**CCGGTTTGATTTATGAGGAGA**	histone cluster 1, H4b	SI00437892	Hs_HIST1H4B_1
		**AAGGCGGTAAAGGTTTGGGTA**		SI00437899	Hs_HIST1H4B_2
		**CGCGGTCTCCTGAGAACTTCA**		SI00437934	Hs_HIST1H4C_3
**HIST1H4C**	NM_003542	**CGGCGGCTGAATCTAAGAATA**	histone cluster 1, H4c	SI00437920	Hs_HIST1H4C_1
		**ATGGATGTAGTATATGCCCTA**		SI00437927	Hs_HIST1H4C_2
		**TTGCGTGACAATATCCAAGGA**		SI00437962	Hs_HIST1H4D_3
**HIST1H4D**	NM_003539	**CGGCGGCTGAGCTTACCTCTA**	histone cluster 1, H4d	SI00437948	Hs_HIST1H4D_1
		**CTCGCGGAGTGCTGAAAGTTT**		SI00437955	Hs_HIST1H4D_2
		**CATCTCGACTTCCCAAATCAA**		SI00437990	Hs_HIST1H4E_3
**HIST1H4E**	NM_003545	**GCGGCTAATGCTACCGCTTAA**	histone cluster 1, H4e	SI00437976	Hs_HIST1H4E_1
		**CCGTAAGGTCCTGCGAGATAA**		SI00437983	Hs_HIST1H4E_2
		**AAAGTGCTGCGTGACAACATA**		SI00438018	Hs_HIST1H4F_3
**HIST1H4F**	NM_003540	**AAGGTGGTAAAGGTTTAGGAA**	histone cluster 1, H4f	SI00438004	Hs_HIST1H4F_1
		**ACGCATTTCGGGCCTCATTTA**		SI00438011	Hs_HIST1H4F_2
		**AAGGCTTTGGCGGTTAAGTGA**		SI00438046	Hs_HIST1H4G_3
**HIST1H4G**	NM_003547	**AAGGTACTGAGCGATAATATT**	histone cluster 1, H4g	SI00438032	Hs_HIST1H4G_1
		**GCGCATCTTGGGCCTCATTTA**		SI00438039	Hs_HIST1H4G_2
		**AAGGGAGGAGCTAAGCGTCAT**		SI00438074	Hs_HIST1H4H_3
**HIST1H4H**	NM_003543	**CTCCTGCTTGCTGCACTCTTA**	histone cluster 1, H4h	SI00438060	Hs_HIST1H4H_1
		**CTGGCCGTGGTAAAGGTGGAA**		SI00438067	Hs_HIST1H4H_2
		**CTCTATGGCTTCGGCGGCTAA**		SI04223016	Hs_HIST1H4I_5
**HIST1H4I**	NM_003495	**CCGCAAGGTGCTGCGCGACAA**	histone cluster 1, H4i	SI00438102	Hs_HIST1H4I_3
		**CCGCGGAGTGTTGAAGGTGTT**		SI00438109	Hs_HIST1H4I_4

**HIST1H4J**	NM_021968	**CGGCGCTAAGCGCCACCGTAA**	histone cluster 1, H4j	SI00438130	Hs_HIST1H4J_3
		**AGCGTCCCTTTCTATCAATAA**		SI00438116	Hs_HIST1H4J_1
		**CCGTAAAGTACTGCGCGACAA**		SI00438123	Hs_HIST1H4J_2
**HIST1H4K**	NM_003541	**CTCTACGGTTTCGGTGGTTGA**	histone cluster 1, H4k	SI00438165	Hs_HIST1H4K_4
		**CCGTAAAGTACTGCGCGACAA**		SI00438151	Hs_HIST1H4K_2
		**CGGCGCTAAGCGCCACCGTAA**		SI00438158	Hs_HIST1H4K_3
**HIST1H4L**	NM_003546	**AAAGTTCTGCGCGACAACATT**	histone cluster 1, H4l	SI00438186	Hs_HIST1H4L_3
		**CGCGGAGTTCTTAAAGTGTTT**		SI00438172	Hs_HIST1H4L_1
		**AAGCGCATCTCAGGCCTTATA**		SI00438179	Hs_HIST1H4L_2
**HIST2H2AA3**	NM_003516	**CCAGGCCGTACTGCTCCCTAA**	histone cluster 2, H2aa3	SI00438214	Hs_HIST2H2AA_3
		**CAGCCCGGCCCGCGTCTCGAA**		SI00438200	Hs_HIST2H2AA_1
		**CTCGGTGACTACTATCGCTGT**		SI00438207	Hs_HIST2H2AA_2
**HIST2H2AB**	NM_175065	**CCAGGCTGTCCTGTTGCCCAA**	histone cluster 2, H2ab	SI00438242	Hs_HIST2H2AB_3
		**CTGGCAAGAACAAGTAATTAA**		SI00438228	Hs_HIST2H2AB_1
		**GACGAAGAGCTCAACAAGTTA**		SI00438235	Hs_HIST2H2AB_2
**HIST2H2AC**	NM_003517	**CCAGGCCGTTCTGTTACCAAA**	histone cluster 2, H2ac	SI00438270	Hs_HIST2H2AC_3
		**AAGCAAATAAATGCAGACAAA**		SI00438256	Hs_HIST2H2AC_1
		**ACCGAAAGCCACAAAGCCAAA**		SI00438263	Hs_HIST2H2AC_2
**HIST2H2BE**	NM_003528	**TTGGGCGACAAGAATATTCAA**	histone cluster 2, H2be	SI00438305	Hs_HIST2H2BE_4
		**CACAGACTGAATAGATCTTAA**		SI00438284	Hs_HIST2H2BE_1
		**TTGGGTCTGTTTCATTCTAAA**		SI00438298	Hs_HIST2H2BE_3
**HIST2H3A**	NM_001005464	**CACGGAGCTGCTGATCCGCAA**	histone cluster 2, H3a	SI04145687	Hs_HIST2H3A_1
		**ATCCGTGGAGAGCGGGCTTAA**		SI00433188	Hs_H3/o_1
		**CGCCAAGCGCGTGACCATTAT**		SI00433195	Hs_H3/o_2
**HIST2H3C**	NM_021059 XM_001124743	**TGCGCTGGTAAGCCTGTGTTT**	histone cluster 2, H3c	SI00438312	Hs_HIST2H3C_1
		**CGGAGGTTCCATCGTATCCAA**		SI02652930	Hs_HIST2H3C_5
		**CACGGAGCTGCTGATCCGCAA**		SI02652986	Hs_HIST2H3C_6
**HIST2H4A**	NM_003548 XM_001124639	**GCGGATCTCTGGCCTCATTTA**	histone cluster 2, H4a	SI00438347	Hs_HIST2H4_2
		**CCGCAAGGTCTTGAGAGACAA**		SI00438340	Hs_HIST2H4_1
		**CCGGCAGAGGAAAGGGCGGAA**		SI00438354	Hs_HIST2H4_3
**HIST3H2A**	NM_033445	**CAAGGCGCGCGCCAAGGCTAA**	histone cluster 3, H2a	SI03157616	Hs_HIST3H2A_5
		**CCGCGGTGCTCGAGTACTTGA**		SI00438368	Hs_HIST3H2A_1
		**CGGAAATGTCCGGTCGTGGTA**		SI00438375	Hs_HIST3H2A_2
**HIST3H2BB**	NM_175055	**CCTCGGCGTCCTGAACCCAAA**	histone cluster 3, H2bb	SI00438410	Hs_HIST3H2BB_3
		**TTGGAGAGACTCAGCCATCAT**		SI00438396	Hs_HIST3H2BB_1
		**GCGCCCAAGAAGGGTTCTAAA**		SI00438403	Hs_HIST3H2BB_2
**HIST3H3**	NM_003493	**AGCAGACTGCGCGCAAGTCAA**	histone cluster 3, H3	SI03143168	Hs_HIST3H3_5
		**GAGGGCTATCTCGCCACCTGA**		SI00438424	Hs_HIST3H3_1
		**ACGGGTCACCATCATGCCTAA**		SI00438445	Hs_HIST3H3_4
**HIST4H4**	NM_175054	**CGGGACAATATCCAAGGCATT**	histone cluster 4, H4	SI00438466	Hs_HIST4H4_3
		**CAGCTTCTCTACAGACTCCAA**		SI00438452	Hs_HIST4H4_1
		**CAGAATGTCTGGGCGAGGTAA**		SI00438459	Hs_HIST4H4_2
**HLTF**	NM_003071 NM_139048	**ACGGGAGTAGTTAATAATAAT**	helicase-like transcription factor	SI02665740	Hs_SMARCA3_7
		**AAGGAATATAATGTTAACGAT**		SI00047558	Hs_SMARCA3_2
		**AACAATGTGAATGGAAATCAA**		SI02665733	Hs_SMARCA3_6
**HMG1L1**	NM_001008735	**ACCATGTCTGCTAAAGAGAAA**	high-mobility group (nonhistone chromosomal) protein 1-like 1	SI00439397	Hs_HMG1L1_4
		**AAGGATATTGCTGCATATCAA**		SI00439383	Hs_HMG1L1_2
		**AAGGGAGTTGTCAAAGCTGAA**		SI00439390	Hs_HMG1L1_3
**HMG20A**	NM_018200	**CACCATTGACTCATATATGAA**	high-mobility group 20A	SI00439418	Hs_HMG20A_3
		**AAGGAAGATAACGGACTGTAA**		SI00439404	Hs_HMG20A_1
		**CAGGAATGCATGAGATTTCAA**		SI00439411	Hs_HMG20A_2
**HMG4L**	NR_002165	**GAGAGAGTCTAAATTCGATTA**	high-mobility group (nonhistone chromosomal) protein 4-like	SI03685388	Hs_HMG4L_3
		**GTGGAAGACAATGTCTGGGAA**		SI03685374	Hs_HMG4L_1
		**GACGTGCAGAGAAGAGCATAA**		SI03685381	Hs_HMG4L_2
**HMGA1**	NM_002131 NM_145899 NM_145901 NM_145902 NM_145903 NM_145904 NM_145905	**CAGCGAAGTGCCAACACCTAA**	high mobility group AT-hook 1	SI00439474	Hs_HMGA1_3
		**CACCACAACTCCAGGAAGGAA**		SI02662569	Hs_HMGA1_6
		**CTGGACAAGGCTAACATCCCA**		SI02662023	Hs_HMGA1_5
		**CTGGACAAGGCTAACATCCCA**		SI02662023	Hs_HMGA1_5
		**CAGCGAAGTGCCAACACCTAA**		SI00439474	Hs_HMGA1_3
		**CACCACAACTCCAGGAAGGAA**		SI02662569	Hs_HMGA1_6
**HMGA2**	NM_001015886 NM_003483 NM_003484	**AACGGCCAAGAGGCAGACCTA**	high mobility group AT-hook 2	SI03029929	Hs_HMGA2_6
		**CCAAGGCACTTTCAATCTCAA**		SI03074302	Hs_HMGA2_8
		**CAGCGGCGGCAGCCTAAGCAA**		SI03067393	Hs_HMGA2_7
**HMGB1**	NM_002128	**AAGAGTAATCAATCTACTCAA**	high-mobility group box 1	SI02627828	Hs_HMGB1_2
		**TCCTTTCATATAGTTAGCTAA**		SI02627842	Hs_HMGB1_4
		**AACCTTTACATGAGGACTCTA**		SI02627835	Hs_HMGB1_3
**HMGB2**	NM_002129	**AGGAATTAGTATCAATAGTAA**	high-mobility group box 2	SI00033040	Hs_HMGB2_2
		**TTGCTAAGAATGTGAATTCAA**		SI00033047	Hs_HMGB2_3
		**GAGGATTGCGTTAGAGATAAA**		SI00033033	Hs_HMGB2_1
**HMGB3**	NM_005342	**AAGAGTCACTGCATCAATGAA**	high-mobility group box 3	SI00439530	Hs_HMGB3_3
		**AGGAGTTAGGAGAACGACATA**		SI03145849	Hs_HMGB3_5
		**CTAGAAATTGTCAGTGGTTTA**		SI00439537	Hs_HMGB3_4
**HMGB4**	NM_001008728 NM_145205	**AAGCCCGATACCAGGAAGAAA**	high-mobility group box 4	SI00474250	Hs_LOC127540_3
		**CCAGGAAGAAATGATGAATTA**		SI00474236	Hs_LOC127540_1
		**TTGGCTTTAAAGAGTTCTCTA**		SI00474243	Hs_LOC127540_2
		**AAGCCCGATACCAGGAAGAAA**		SI00474250	Hs_LOC127540_3
		**TTGGCTTTAAAGAGTTCTCTA**		SI00474243	Hs_LOC127540_2
		**CCAGGAAGAAATGATGAATTA**		SI00474236	Hs_LOC127540_1
**HMGN1**	NM_004965	**CAGATTTGAACATGTAGTGTA**	high-mobility group nucleosome binding domain 1	SI00439572	Hs_HMGN1_1
		**ACCAACGTGAAGATACTGGTA**		SI00439586	Hs_HMGN1_3
		**CTGATTAATAACCATATACCA**		SI00439579	Hs_HMGN1_2
**HMGN2**	NM_005517	**TGGATACAGTTTGGCTTGGAA**	high-mobility group nucleosomal binding domain 2	SI02652972	Hs_HMGN2_5
		**GAGCCCGAGCAGTGTGAAGAA**		SI02635402	Hs_HMGN2_2
		**CACTAATAGAATGTCTCCAAA**		SI00078260	Hs_HMGN2_1
**HMGN3**	NM_004242 NM_138730	**GAGGGCAAAGATGGATCCAAA**	high mobility group nucleosomal binding domain 3	SI00061579	Hs_HMGN3_3
		**AACCTGAACCCAAACCAAGAA**		SI03029054	Hs_HMGN3_6
		**CTGCTGACTATTTGTGGTTTA**		SI00061586	Hs_HMGN3_4
**HMGN4**	NM_006353	**CTGCCTCTTTCATAAATTAAA**	high mobility group nucleosomal binding domain 4	SI00439600	Hs_HMGN4_1
		**CAGCAACGACATATCTTGTTA**		SI00439614	Hs_HMGN4_3
		**TTGGATTTGTATTGAATTTAT**		SI00439607	Hs_HMGN4_2
**HP1BP3**	NM_016287	**CCCAAGATGGATGCAATCTTA**	heterochromatin protein 1, binding protein 3	SI00441154	Hs_HP1-BP74_3
		**TCGGGCCTAGAAATTATTTAA**		SI00441140	Hs_HP1-BP74_1
		**CACACTGAAACCATTAGGAAA**		SI00441147	Hs_HP1-BP74_2
**HTATIP**	NM_006388 NM_182709 NM_182710	**CACCACATTGCCTGTCCTCTA**	HIV-1 Tat interacting protein, 60kDa	SI02780897	Hs_HTATIP_8
		**CACCCGGATGAAGAACATTGA**		SI00162981	Hs_HTATIP_5
		**CGGCGGAACCAGGACAACGAA**		SI00088053	Hs_HTATIP_2
**ING2**	NM_001564	**CTGGACAACAAATATCAAGAA**	inhibitor of growth family, member 2	SI00447657	Hs_ING1L_4
		**AGAGCACTAATTAATAGTCAA**		SI00447643	Hs_ING1L_2
		**ACCAGTGAAAGCCGTGATTTA**		SI00447650	Hs_ING1L_3
**JHDM1D**	NM_030647 XM_376680 XM_379934	**CAGAGTGTCGCTGGTAAATAA**	jumonji C domain-containing histone demethylase 1 homolog D (S. cerevisiae)	SI00460390	Hs_KIAA1718_3
		**ACTGCTAGGCATAATTATCAA**		SI00460376	Hs_KIAA1718_1
		**ATGGCAGTAACCATGAGGTTA**		SI00460383	Hs_KIAA1718_2
**KCMF1**	NM_020122 XM_001133393	**ATGCCTTTAGATGTTGCTTTA**	potassium channel modulatory factor 1	SI00450842	Hs_KCMF1_3
		**CTCCTAATACTCCACATTCAA**		SI00450828	Hs_KCMF1_1
		**CAAGAACATGTTACTTCTGAA**		SI00450835	Hs_KCMF1_2
**KLHDC3**	NM_057161	**CACCATGACATGGACTCTTAT**	kelch domain containing 3	SI00463442	Hs_KLHDC3_3
		**TCCAATGACATTCACAAGCTA**		SI00463428	Hs_KLHDC3_1
		**CAGGTTCTGCTGGGAAGGGAA**		SI00463435	Hs_KLHDC3_2
**L3MBTL**	NM_015478 NM_032107	**GCCAGTGATAGTCAATATTAA**	l(3)mbt-like (Drosophila)	SI00467530	Hs_L3MBTL_3
		**TCCAATATAGTTGATAATTAA**		SI00467516	Hs_L3MBTL_1
		**CCAGTGATAGTCAATATTAAA**		SI00467523	Hs_L3MBTL_2
**L3MBTL2**	NM_001003689 NM_031488	**AGCCATGTAAATTAAGTTCTA**	l(3)mbt-like 2 (Drosophila)	SI00467551	Hs_L3MBTL2_2
		**TCCCGTGGATTTCCACATCAA**		SI00467544	Hs_L3MBTL2_1
		**CAAGATTCCTTTGTAGTTAAA**		SI00467558	Hs_L3MBTL2_3
**LIG3**	NM_002311 NM_013975	**CTGGGAAGAGCTGGAAGATAA**	ligase III, DNA, ATP-dependent	SI02757489	Hs_LIG3_8
		**CTGGAAGATAATGAGAAGGAA**		SI02663549	Hs_LIG3_7
		**CACCCGAATCCGAGATGATAA**		SI00035994	Hs_LIG3_1
**LIG4**	NM_001098268 NM_002312 NM_206937	**AACCTTACTAACGTTAACAAA**	ligase IV, DNA, ATP-dependent	SI00036043	Hs_LIG4_4
		**TGGTGAGATGATGGCCTATAA**		SI03021179	Hs_LIG4_7
		**CACCGTTTATTTGGACTCGTA**		SI00036022	Hs_LIG4_1
**LOC120126**	NM_001101321 XM_061880 XM_936270	**AACAGGAAGAAAGATAGAGAA**	hypothetical protein	SI02793525	Hs_LOC642114_1
		**CAAGAAATCTAGTGTCTCCAA**		SI02793532	Hs_LOC642114_2
**LOC129870**	XM_065445	**AACGCAGACATCTTGAGGTTA**	similar to Nucleolar transcription factor 1 (Upstream-binding factor 1) (UBF-1) (Autoantigen NOR-90)	SI03125703	Hs_LOC129870_5
		**CAGAAGGAATACAAAGTGGAA**		SI00474915	Hs_LOC129870_2
		**CTCCAAGAGGATTCAAACCAA**		SI00474929	Hs_LOC129870_4
**LOC196346**	XM_115100	**ATAGTCAAGGTGGTTCTTGAA**	similar to tripartite motif protein 39	SI03148355	Hs_LOC196346_6
		**CTGCAGCATGGGTTTCCATAA**		SI00484694	Hs_LOC196346_3
		**AAGAATAGTACAATGGTTAAA**		SI03127789	Hs_LOC196346_5
**LOC203510**	XM_115897 XM_937321	**CACTAAGGCAGCAGACCTGAA**	similar to high-mobility group box 3	SI00486647	Hs_LOC203510_2
		**GTGAGATGTGGATTAACTATA**		SI00486661	Hs_LOC203510_4
**LOC255519**	XM_171766 XR_016784 XR_018925	**CAGAGGTTCTTGTCAATTTCA**	similar to high-mobility group box 3	SI00488754	Hs_LOC255519_3
		**AAGGCTGAGTGAGATATGGAA**		SI00488740	Hs_LOC255519_1
		**CAGAGAAGAATGCAAGAAGAA**		SI00488747	Hs_LOC255519_2
		**GAGGCTTGATCATGGACTTAA**		SI00494774	Hs_LOC285412_3

**LOC285412**	XM_208319 XR_017405 XR_019409	**AAGCAGAAATACCAGTGGTAA**	similar to Epidermal Langerhans cell protein LCP1	SI00494760	Hs_LOC285412_1
		**CAGCTGCTACTGTTGCTTCTA**		SI00494767	Hs_LOC285412_2
**LOC340096**	XM_293943	**CTGGCGCTGCGGGAAATCAGA**	similar to HIStone family member (his-72)	SI03211789	Hs_LOC340096_5
		**CCTGGGAAACCTTGCACTCTA**		SI00500808	Hs_LOC340096_1
		**CTCCGTGGAGAGGGTGCCGGA**		SI00500815	Hs_LOC340096_2
**LOC388177**	XM_370909 XM_938956	**CCGCAGGATCCTATTACTTCT**	similar to H2A histone family, member V isoform 2	SI00512330	Hs_LOC388177_3
		**ATGGGTGATAGTGGCTTTAAA**		SI00512316	Hs_LOC388177_1
		**AAGCCCTCTCAAAGACACTAA**		SI00512323	Hs_LOC388177_2
**LOC388553**	XM_373809 XM_944980	**CAGGTTCAGCCGCCAGCACAA**	hypothetical LOC388553	SI00515585	Hs_LOC388553_4
		**CAGGCAGAAGCTGGTGTTCTA**		SI00515564	Hs_LOC388553_1
		**CAGGATGAACCGGAAGCAGTA**		SI00515571	Hs_LOC388553_2
**LOC389168**	XM_371672 XR_017613 XR_019471	**AAGGGTTGGTTTGAGATGTTT**	similar to Protein SET (Phosphatase 2A inhibitor I2PP2A) (I-2PP2A) (Template-activating factor I) (TAF-I) (HLA-DR-associated protein II) (PHAPII) (Inhibitor of granzyme A-activated DNase) (IGAAD)	SI00521626	Hs_LOC389168_3
		**ATGGCCAAGCATGACATCAGT**		SI00521619	Hs_LOC389168_2
		**AAGCTTGCTGAAGAAGGGAGA**		SI00521612	Hs_LOC389168_1
**LOC389217**	XM_371701	**ACAACATTTGGCAATCATTTA**	similar to Protein SET (Phosphatase 2A inhibitor I2PP2A) (I-2PP2A) (Template-activating factor I) (TAF-I) (HLA-DR-associated protein II) (PHAPII) (Inhibitor of granzyme A-activated DNase) (IGAAD)	SI00522193	Hs_LOC389217_4
		**AAGGATGAAAGAGATGACTAA**		SI00522172	Hs_LOC389217_1
		**TGGGTTGTGACAAGCAAGAAA**		SI00522179	Hs_LOC389217_2
**LOC390245**	XM_372429	**CCCGGTAATCCACCAATTTAT**	similar to jumonji domain containing 2D	SI00529865	Hs_LOC390245_4
		**CAGATGTATGATGATATCGAA**		SI00529844	Hs_LOC390245_1
		**CAGGGCACTGTTACAACCCAA**		SI00529858	Hs_LOC390245_3
**LOC391566**	XM_373001	**CAAGAGTTGCAGAGGAAGGAA**	similar to Histone H2B 291B	SI00536298	Hs_LOC391566_3
		**AACGATTCAATGAGAAGCTAA**		SI00536284	Hs_LOC391566_1
		**GAGATACTTGTTAGAAAGAAA**		SI00536291	Hs_LOC391566_2
**LOC391769**	XM_373079	**CTCCGCAGAGAGGGTCCTTAA**	similar to HIStone family member (his-72)	SI00537348	Hs_LOC391769_1
		**CACGCAGCTGCTCCTGCGCAA**		SI00537355	Hs_LOC391769_2
**LOC90826**	NM_138364	**CAGACAAGTTATGGAGCATAA**	hypothetical protein BC004337	SI00150598	Hs_LOC90826_1
		**CACCAAGAGGAATACAATTTA**		SI02779308	Hs_LOC90826_6
		**GAGGATGAATCTGCTATGTTA**		SI00150605	Hs_LOC90826_2
**MBD1**	NM_002384 NM_015844 NM_015845 NM_015846 NM_015847	**CAGGATCCGAAGCAAAGTTGA**	methyl-CpG binding domain protein 1	SI00036939	Hs_MBD1_4
		**AAGACTGTCGAGCACAGAGAA**		SI03031602	Hs_MBD1_7
		**ACCGGGAACAGAGAATGTTTA**		SI03040254	Hs_MBD1_8
**MBD2**	NM_003927 NM_015832	**AAGATGATGCCTAGTAAATTA**	methyl-CpG binding domain protein 2	SI02663787	Hs_MBD2_8
		**TGGAAAGATGATGCCTAGTAA**		SI02632280	Hs_MBD2_7
		**TGGAAAGATGATGCCTAGTAA**		SI02632280	Hs_MBD2_7
		**CAAGTGGTAAGAAGTTCAGAA**		SI03054996	Hs_MBD2_9
		**CAAGTGGTAAGAAGTTCAGAA**		SI03054996	Hs_MBD2_9
		**AAGATGATGCCTAGTAAATTA**		SI02663787	Hs_MBD2_8
**MBD3**	NM_003926	**GCCGGTGACCAAGATTACCAA**	methyl-CpG binding domain protein 3	SI02664886	Hs_MBD3_5
		**CCCGGAGATGGAGCACGTCTA**		SI00057848	Hs_MBD3_2
		**CCAGACGGCGTCCATCTTCAA**		SI00057855	Hs_MBD3_3
**MBD3L1**	NM_145208	**AGGGAAGATAGTGCAGATGAA**	methyl-CpG binding domain protein 3-like 1	SI00628495	Hs_MBD3L1_1
		**CTCGCTAATGAGGCAGAGAAA**		SI00628502	Hs_MBD3L1_2
		**TAAAGTGTAATCCTTTATTAA**		SI00628509	Hs_MBD3L1_3
**MBD3L2**	NM_144614 XM_001131059	**CAGGAGGAACTCTACAGCTAT**	methyl-CpG binding domain protein 3-like 2	SI00628523	Hs_MBD3L2_1
		**CTGCATTTGGAGAGCGTCTTA**		SI00628537	Hs_MBD3L2_3
		**AAGGATCAGGTCTCATCCTGA**		SI04314758	Hs_MBD3L2_7
**MBD4**	NM_003925	**AAGCTTCTCATCGCTACTATA**	methyl-CpG binding domain protein 4	SI02663780	Hs_MBD4_5
		**CCGCCGAATGACCTCCGCAAA**		SI00057827	Hs_MBD4_3
		**AAGAGAATCTGTGTGTAATAA**		SI00057834	Hs_MBD4_4
**MBD5**	NM_018328	**AAGGTGATATGTCATCAATAA**	methyl-CpG binding domain protein 5	SI00628558	Hs_MBD5_2
		**CCGGAGGAGGAACAAATGCAA**		SI00628565	Hs_MBD5_3
		**AAGGAATTACAAATTCTGTAA**		SI00628572	Hs_MBD5_4
**MBD6**	NM_052897	**ATGGGAGACACTCTTAATTTA**	methyl-CpG binding domain protein 6	SI00628579	Hs_MBD6_1
		**CCAGTGAAAGGTACAAAGCAA**		SI00628586	Hs_MBD6_2
		**CCCACTCTGATGGAAGCTTTA**		SI00628593	Hs_MBD6_3
**MCM2**	NM_004526	**CAGCGACATGTGCAAAGAGAA**	minichromosome maintenance complex component 2	SI00064925	Hs_MCM2_2
		**CTCATTGGAGATGGCATGGAA**		SI02653525	Hs_MCM2_6
		**CTGCGGGACTATGTGATCGAA**		SI00064918	Hs_MCM2_1
**MCM7**	NM_005916 NM_182776	**CAGAGGAGGATTTCTACGAAA**	minichromosome maintenance complex component 7	SI00629090	Hs_MCM7_2
		**CAGGCTAATGGAGATGTCAAA**		SI00629083	Hs_MCM7_1
		**CTGGAGCAGAACATACAGCTA**		SI00629097	Hs_MCM7_3
**MECP2**	NM_004992	**CCCATTCTGTCCTACCTTTAA**	methyl CpG binding protein 2 (Rett syndrome)	SI00071099	Hs_MECP2_3
		**CAGAGATGTCTCTTATATCTA**		SI02634205	Hs_MECP2_6
		**ACGGAGCGGATTGCAAAGCAA**		SI02664893	Hs_MECP2_7
**MGC21874**	NM_152293 XM_001128012 XM_001128029 XM_001128049 XM_291105	**CTGGCTAGTAGGCATCTGTAA**	transcriptional adaptor 2 (ADA2 homolog, yeast)-beta	SI00635369	Hs_MGC21874_3
		**AACCCATTGTACAAAGTTCAA**		SI00635355	Hs_MGC21874_1
		**CCGGCGAAACGGGATCACCAA**		SI00635362	Hs_MGC21874_2
**MIZF**	NM_015517 NM_198971	**TTGAGCGTGTTTATTAACAAA**	MBD2-interacting zinc finger	SI00645981	Hs_MIZF_3
		**CAGGGCATTATTCTAGAAACA**		SI00645967	Hs_MIZF_1
		**CCCAAGGACTTTCATGATTAT**		SI00645974	Hs_MIZF_2
**MLL**	NM_005933	**CCGCTACTGATCTTGAATCAA**	myeloid/lymphoid or mixed-lineage leukemia (trithorax homolog, Drosophila)	SI00082866	Hs_MLL_4
		**TGGGATCTAGTTCCAGAGATA**		SI02636305	Hs_MLL_6
		**CTCGATCAAATGCCCGCCTAA**		SI00082845	Hs_MLL_1
**MLL2**	NM_003482	**CCCGTTGAGTCCCATAGGGAA**	myeloid/lymphoid or mixed-lineage leukemia 2	SI00052451	Hs_MLL2_2
		**CTCGGAGTGGTTTGAGAACTA**		SI00052458	Hs_MLL2_3
		**CGGCCGGAGTTTGTAATCAAA**		SI00052444	Hs_MLL2_1
**MLL3**	NM_021230 NM_170606	**CGGGATCAACCCAATCGCTCA**	myeloid/lymphoid or mixed-lineage leukemia 3	SI03086986	Hs_MLL3_6
		**ATCAGAGTTCACGACCATTAA**		SI00130382	Hs_MLL3_4
		**CAGCGGCAGAAGTTACGTGAA**		SI03067316	Hs_MLL3_5
**MLL4**	NM_014727	**CACGAGCTGGTGTTCTGTCAA**	myeloid/lymphoid or mixed-lineage leukemia 4	SI00107751	Hs_MLL4_3
		**CCCAGCGTTGCCAGCACTATA**		SI00107737	Hs_MLL4_1
		**CTCGAATCAGGTCAAGGTCAA**		SI00107744	Hs_MLL4_2
**MORF4**	NM_006792	**ACCCAGCTACTCAACAAATTT**	mortality factor 4	SI00647353	Hs_MORF4_3
		**GCCGAAATTCTTGCAGATTGT**		SI00647339	Hs_MORF4_1
		**GGGCACCCAGCTACTCAACAA**		SI00647346	Hs_MORF4_2
**MORF4L1**	NM_006791 NM_206839	**CCGGAGAGCAGAGTACTCAAA**	mortality factor 4 like 1	SI00647388	Hs_MORF4L1_4
		**TCCGGAGAGCAGAGTACTCAA**		SI00647367	Hs_MORF4L1_1
		**AAGGTTCAATTCAGTGTATAT**		SI00647381	Hs_MORF4L1_3
**MORF4L2**	NM_012286	**TAGGAGTTCTTGGAAGGGAAA**	mortality factor 4 like 2	SI00647409	Hs_MORF4L2_3
		**AAGATTGTTGGTTGGAATAAA**		SI00647395	Hs_MORF4L2_1
		**TAGAATGGAGGTTAAAGTGAA**		SI00647402	Hs_MORF4L2_2
**MPHOSPH8**	NM_017520	**CAGCAGTTTGGAATTCTTCTA**	M-phase phosphoprotein 8	SI00442862	Hs_HSMPP8_3
		**AAGGGTGAAATAAGAGATTTA**		SI00442848	Hs_HSMPP8_1
		**CTGGTGAACTTCTGTGCTTAA**		SI00442855	Hs_HSMPP8_2
**MSL3L1**	NM_006800 NM_078628 NM_078629 NM_078630	**CTGGTGTGGACTCTGTCTTAA**	male-specific lethal 3-like 1 (Drosophila)	SI00650321	Hs_MSL3L1_3
		**CAGGAGGAAACGGTTAGTGAA**		SI00650307	Hs_MSL3L1_1
		**ACAGCGTAAATTGGCAAGAAA**		SI00650314	Hs_MSL3L1_2
**MSL3L2**	XM_087225 XM_939716	**CAGGCAGTCAATAAGTGTTGA**	male-specific lethal 3-like 2 (Drosophila)	SI00481642	Hs_LOC151507_3
		**CAAGGTTTAGTTTGTACTTGA**		SI00481628	Hs_LOC151507_1
		**AAGCAACTGTTCACAGTGAAA**		SI00481635	Hs_LOC151507_2
**MTA2**	NM_004739	**CAGCCAAGCGTCAGAAACTAA**	metastasis associated 1 family, member 2	SI02780834	Hs_MTA2_5
		**ACGGCCTTATGCTCCTATCAA**		SI00067781	Hs_MTA2_2
		**ACGCCAGTTCTCAGAAATAAA**		SI00067774	Hs_MTA2_1
**MTA3**	NM_020744	**CTCGCAGATAAGCATGCTAAA**	metastasis associated 1 family, member 3	SI00650797	Hs_MTA3_3
		**ACGGCCGTTTGTTGCTATTAA**		SI00650790	Hs_MTA3_2
		**CAGATGAGAGGGAACAATCAA**		SI00650804	Hs_MTA3_4
**MYST1**	NM_032188 NM_182958	**CGGGAAAGAGATCTACCGCAA**	MYST histone acetyltransferase 1	SI00654031	Hs_MYST1_1
		**CAGCCTGTAAATATGTATAGA**		SI00654045	Hs_MYST1_3
		**CTGCCTCAAGTACATGAAATA**		SI00654038	Hs_MYST1_2
**MYST2**	NM_007067	**ACAGCTGATCATGATGAGTCA**	MYST histone acetyltransferase 2	SI00094332	Hs_MYST2_1
		**CTCGTTCATCTGGTTCAGAAA**		SI00094346	Hs_MYST2_3
		**CAGGCAGTAGTTCAGATGGAA**		SI00094339	Hs_MYST2_2
**MYST3**	NM_001099412 NM_001099413 NM_006766	**CGGCGCTATACTAATCCAATA**	MYST histone acetyltransferase (monocytic leukemia) 3	SI00091700	Hs_MYST3_1
		**CAGCTGAATTTGCGACCTGTA**		SI00091714	Hs_MYST3_3
		**CTGGACTTCCGTAGTGACCAA**		SI00091707	Hs_MYST3_2
**MYST4**	NM_012330	**CTGAATTATCTTCCACGGCAA**	MYST histone acetyltransferase (monocytic leukemia) 4	SI00099484	Hs_MYST4_1
		**CCCAAACGTATGCGTCGTAAA**		SI00099498	Hs_MYST4_3
		**ACGGAACATGGCTGCATCAAA**		SI00099491	Hs_MYST4_2
**NAP1L1**	NM_004537 NM_139207	**CTGGAAATCCTTAACCAATTA**	nucleosome assembly protein 1-like 1	SI00654871	Hs_NAP1L1_1
		**ATGGCAGACATTGACAACAAA**		SI03019296	Hs_NAP1L1_5
		**CACCACGCTTTGATAACTAAA**		SI03019597	Hs_NAP1L1_6
**NAP1L2**	NM_021963	**CAGAGTATACATGGCCCTGAA**	nucleosome assembly protein 1-like 2	SI00654913	Hs_NAP1L2_3
		**AGGGAATTTCATGACATTGAA**		SI00654899	Hs_NAP1L2_1
		**CACACTAGAATTTCACTTCAA**		SI00654906	Hs_NAP1L2_2
**NAP1L3**	NM_004538	**TAGGCGGTTTCAAATCATCAA**	nucleosome assembly protein 1-like 3	SI00654941	Hs_NAP1L3_3
		**CAGGACAGTGTAGATCTTAAA**		SI00654927	Hs_NAP1L3_1
		**CCAGTACTTATAGTAAATAAA**		SI00654934	Hs_NAP1L3_2
**NAP1L4**	NM_005969	**CAGAATAGTTAGAATATTCTA**	nucleosome assembly protein 1-like 4	SI00654962	Hs_NAP1L4_2
		**TCCAGCTACATCGAAACTTTA**		SI00654976	Hs_NAP1L4_4
		**CTGGTTTACCATCTTCAGAAA**		SI00654969	Hs_NAP1L4_3
**NAP1L5**	NM_153757	**ATGTGACATATTAATTATAAA**	nucleosome assembly protein 1-like 5	SI00654997	Hs_NAP1L5_3
		**CAGAATATCGATGGACTTAAA**		SI00654983	Hs_NAP1L5_1
		**CCCAAATATGTTTAAGTATTA**		SI00654990	Hs_NAP1L5_2
	NM_002482 NM_152298	**TAGGATGGTTCTTGTCCTATA**		SI00655228	Hs_NASP_4

**NASP**	NM_002482 NM_152298 NM_172164	**CAGAATTGACTTCTCAAATAA**	nuclear autoantigenic sperm protein (histone-binding)	SI00655207	Hs_NASP_1
		**CTCGGAGAAGTTAGTGTTGAA**		SI00655214	Hs_NASP_2
		**TTGCATCACTTTCGAAGTATA**		SI00349013	Hs_CNAP1_1
**NCAPD2**	NM_014865	**CAGGAGGTGATTGAATTCTTT**	non-SMC condensin I complex, subunit D2	SI00349020	Hs_CNAP1_2
		**CAAGTCAGTGCTAGTATGTAA**		SI00349027	Hs_CNAP1_3
		**CTCCTAATATTTCGACATTAA**		SI02631783	Hs_NCOA1_14
**NCOA1**	NM_003743 NM_147223 NM_147233	**CAGCAGCTACTGACTGAATAA**	nuclear receptor coactivator 1	SI02631797	Hs_NCOA1_16
		**CAGCGGGAACTGTACAGTCAA**		SI00055342	Hs_NCOA1_1
		**CAGGAGGAGATTATAATACTT**		SI00089369	Hs_NCOA3_1
**NCOA3**	NM_006534 NM_181659	**CAGGATTATATGGACAGACAT**	nuclear receptor coactivator 3	SI00089383	Hs_NCOA3_3
		**AAGGTTGTCAATATAGATACA**		SI00089376	Hs_NCOA3_2
		**AAGGAGCTAATGCCATTATAA**		SI02640225	Hs_NCOA6_7
**NCOA6**	NM_014071	**CAGCGTGCGTGTGACATTCAA**	nuclear receptor coactivator 6	SI02655485	Hs_NCOA6_8
		**CCACAGATTAATATTCCTTTA**		SI02640218	Hs_NCOA6_6
		**CCCGCTCACCAGGGAGTATAA**		SI00087297	Hs_NCOR1_1
**NCOR1**	NM_006311	**ACCGCAGTATTGTCCAAATTA**	nuclear receptor co-repressor 1	SI02780862	Hs_NCOR1_5
		**AGGCAATTCAGTGGACTATAA**		SI00087304	Hs_NCOR1_2
		**CAGAATGAATTCGATGCGTAT**		SI00087339	Hs_NCOR2_3
**NCOR2**	NM_001077261 NM_006312	**AACGAGATTGCTGGAAACCAA**	nuclear receptor co-repressor 2	SI00087325	Hs_NCOR2_1
		**AAGGGCCACGTCATCTACGAA**		SI03035970	Hs_NCOR2_6
		**AAGCCCTGTCAAACAAGTCAA**		SI00661101	Hs_NPM2_3
**NPM2**	NM_182795	**CAGGAACGTTATGAAGCATCA**	nucleophosmin/nucleoplasmin, 2	SI00661087	Hs_NPM2_1
		**CAGGCGAGCGTGGCTAAGAAA**		SI00661094	Hs_NPM2_2
		**CTAGTGAAATATTGTGCTTAA**		SI00661668	Hs_NSBP1_4
**NSBP1**	NM_030763	**CAGCCTTTCTTTAGCATTGAT**	nucleosomal binding protein 1	SI00661661	Hs_NSBP1_3
		**TACACCAGAGGTGAAGCCTAA**		SI04195079	Hs_NSBP1_6
		**CTGAAGGAGTTTCCCATCAAA**		SI00676928	Hs_PADI4_4
**PADI4**	NM_012387	**CTGCACGTCCTTCAGCATCAA**	peptidyl arginine deiminase, type IV	SI02657389	Hs_PADI4_5
		**CAAGACATTGAGAGAACATAA**		SI00676921	Hs_PADI4_3
		**CCGATATCTTAGCTTATTGTA**		SI00088466	Hs_PARP4_3
**PARP4**	NM_006437	**CGGCCGTTGGTTCCTATCTTA**	poly (ADP-ribose) polymerase family, member 4	SI02780050	Hs_PARP4_5
		**TAAGTTGGACAGAGTCATGTA**		SI00088473	Hs_PARP4_4
		**CAAGGATGCAAATTCAATTAA**		SI00120925	Hs_PB1_3
**PBRM1**	NM_018165 NM_018313 NM_181041 NM_181042	**CAGTTAGGAGTTGTCGGAATA**	polybromo 1	SI03073413	Hs_PB1_5
		**TCCCAGGTTTATAATGATGCA**		SI00120932	Hs_PB1_4
		**CGGAGTGTACTCCGCCTGCAA**		SI02632147	Hs_PCAF_6
**PCAF**	NM_003884 XM_001130666	**ACAGTCTACCTCGGTACGAAA**	p300/CBP-associated factor	SI03038035	Hs_PCAF_9
		**CAGCAAATAATTGTCAGTCTA**		SI02632154	Hs_PCAF_7
		**TAGGCACTATTCCTTATATAA**		SI00683627	Hs_PHC1_1
**PHC1**	NM_004426	**CAAGATCTGCGCCAAGATAAA**	polyhomeotic homolog 1 (Drosophila)	SI00683641	Hs_PHC1_3
		**CCGGTGGGTTGTTCTCAGTTA**		SI00683634	Hs_PHC1_2
		**CCGGGTGGACTTTGCCTATAA**		SI00683655	Hs_PHC2_1
**PHC2**	NM_004427 NM_198040	**CCCACACTTCTTTGCCTATAA**	polyhomeotic homolog 2 (Drosophila)	SI00683669	Hs_PHC2_3
		**CTGGTTCACAAAGAGCATTAA**		SI00683662	Hs_PHC2_2
		**TTGGCACATATGAGGCTTAAA**		SI00108332	Hs_PJA2_2
**PJA2**	NM_014819	**ATCCACCATACTCAAGAGTTA**	praja 2, RING-H2 motif containing	SI00108339	Hs_PJA2_3
		**CTGGCAAGTTCAGATACTAAA**		SI00108325	Hs_PJA2_1
		**TCCCTAAACCATCCTCTTTAA**		SI00688828	Hs_POLE3_4
**POLE3**	NM_017443	**GCGGTTCGTTATCCCATTGAA**	polymerase (DNA directed), epsilon 3 (p17 subunit)	SI03220721	Hs_POLE3_5
		**AACGACTAAATTAGTCAGTTT**		SI00688821	Hs_POLE3_3
		**CACCCGTGAGAGAGACTTGAA**		SI02647911	Hs_PPARGC1B_6
**PPARGC1B**	NM_133263	**CAGGCGATGGTGCAACTCATA**	peroxisome proliferator-activated receptor gamma, coactivator 1 beta	SI00150024	Hs_PPARGC1B_2
		**AGCCGAAGTTGTACAGAACTA**		SI03043306	Hs_PPARGC1B_7
		**CAAAGTCACCTGCCCAATAAA**		SI00692825	Hs_PRM2_3
**PRM2**	NM_002762	**CCAGCTGGAAGTTAAGAGAAA**	protamine 2	SI00692811	Hs_PRM2_1
		**TCCCAACACCATGGTCCGATA**		SI00692832	Hs_PRM2_4
		**CCGGCAGTACAAAGACTACAA**		SI02663500	Hs_HRMT1L2_8
**PRMT1**	NM_001536 NM_198318 NM_198319	**CACCATCGACCTGGACTTCAA**	protein arginine methyltransferase 1	SI02663493	Hs_HRMT1L2_7
		**CACCAACGCCTGCCTCATAAA**		SI00441840	Hs_HRMT1L2_1
		**AACGCGATTGAGAAATGGAAA**		SI02781009	Hs_HRMT1L1_6
**PRMT2**	NM_001535	**ACCGTGCAAATTTCTGATCTA**	protein arginine methyltransferase 2	SI02780547	Hs_HRMT1L1_5
		**CAGACCAGCCACGAACAACTA**		SI00441826	Hs_HRMT1L1_3
		**CACCGTGACCCTCACGTTGAA**		SI03058699	Hs_PRMT3_1
**PRMT3**	NM_005788	**AAGGTTCTTGGAGTTGATCAA**	protein arginine methyltransferase 3	SI00441882	Hs_HRMT1L3_3
		**TACCGAGATTTCATATACCAA**		SI03108938	Hs_PRMT3_2
		**AGGGCTCAAGCCACCAATCTA**		SI00719425	Hs_SKB1_3
**PRMT5**	NM_001039619 NM_006109	**CAGCCCATAACGGTACGTGAA**	protein arginine methyltransferase 5	SI00719432	Hs_SKB1_4
		**TGGGCAAGGGATTATAATTAA**		SI02657060	Hs_SKB1_5
		**CACACCTTATCTAAGTCTGAA**		SI00120778	Hs_HRMT1L6_2
**PRMT6**	NM_018137	**CCGCAGCGCTTTGCTCAGCTA**	protein arginine methyltransferase 6	SI00120771	Hs_HRMT1L6_1
		**CCGCCTGGGTATCCTTCGGAA**		SI00120785	Hs_HRMT1L6_3
		**CGCGTGTGCCTAGAATATATA**		SI00124502	Hs_HRMT1L4_2
**PRMT8**	NM_019854	**CAGATGTTAAGCAGCTATGAA**	protein arginine methyltransferase 8	SI00124495	Hs_HRMT1L4_1
		**CAAGCACGTGTTCAAGGACAA**		SI03054086	Hs_PRMT8_1
		**TTGGGAATTCTTGCTGTTAAA**		SI00087507	Hs_RAN_3
**RAN**	NM_006325	**TAGTGTGAAGTTGATACGCAA**	RAN, member RAS oncogene family	SI00087493	Hs_RAN_1
		**TTGGCTAGGTAGGGTCACCTA**		SI03025064	Hs_RAN_5
		**CGCGTGTAAATTCTACTGCAA**		SI02653819	Hs_RB1_7
**RB1**	NM_000321	**AAGGACCGAGAAGGACCAACT**	retinoblastoma 1 (including osteosarcoma)	SI00301651	Hs_RB1_5
		**CAGGGTTGTGTCGAAATTGGA**		SI02653931	Hs_RB1_8
		**CAGCTATCCCTCTATATAATA**		SI02653420	Hs_RBBP4_8
**RBBP4**	NM_005610	**ACGAGTGATCAACGAGGAATA**	retinoblastoma binding protein 4	SI00079513	Hs_RBBP4_3
		**AAGACTCCTTCCAGTGATGTT**		SI00301658	Hs_RBBP4_5
		**CACGCTATTCGGGCTCCTATA**		SI02637229	Hs_RBM14_6
**RBM14**	NM_006328	**AACAGTTCGCCTTCGTGCACA**	RNA binding motif protein 14	SI00301665	Hs_RBM14_5
		**TAGGGTTACAGATCCAGGTTA**		SI02637236	Hs_RBM14_7
		**CTGCCAGGTCCAAGCAATATA**		SI00115304	Hs_REV1L_3
**REV1**	NM_001037872 NM_016316	**ATCGGTGGAATCGGTTTGGAA**	REV1 homolog (S. cerevisiae)	SI00115311	Hs_REV1L_4
		**CAGCGCATCTGTGCCAAAGAA**		SI00115297	Hs_REV1L_2
		**TCAGATATCTTTCAAAGTATA**		SI00701526	Hs_RFC1_2
**RFC1**	NM_002913	**CTGGAAGTGTCCAAAGATCTA**	replication factor C (activator 1) 1, 145kDa	SI00701533	Hs_RFC1_3
		**CAGGAATTAATTGGCCTGATA**		SI00701519	Hs_RFC1_1
		**CAGGGTCAGATCAGACCACAA**		SI00045934	Hs_RING1_1
**RING1**	NM_002931	**AACGTGGGAACTGAGTCTGTA**	ring finger protein 1	SI03030244	Hs_RING1_5
		**CCGAAAGAAGCTGGTGTCCAA**		SI00045948	Hs_RING1_3
		**ACGAAGCTCGATGAAGATTTA**		SI00703997	Hs_RNASE3L_3
**RNASEN**	NM_001100412 NM_013235	**CTGGATGGTCTTTATTACAAA**	ribonuclease III, nuclear	SI00703983	Hs_RNASE3L_1
		**AGGGATTAACACCTTGATAAA**		SI00703990	Hs_RNASE3L_2
		**CCCGAAAGCTTCACTAAGTTT**		SI00350112	Hs_COBRA1_2
**RP13-122B23.3**	NM_015456 XM_001133009	**GCGGCAGATCTGGCAAGACAA**	cofactor of BRCA1	SI00350119	Hs_COBRA1_3
		**CACTTTCAATGTGGATCAGAA**		SI00350105	Hs_COBRA1_1
		**CACCGAGTCGTGTACCATGAA**		SI00117005	Hs_HBXAP_3
**RSF1**	NM_016578	**CCGGACGTCGGCAACGGAGAA**	remodeling and spacing factor 1	SI00117012	Hs_HBXAP_4
		**CAGCCAATTGGTCGAGACAAA**		SI00116991	Hs_HBXAP_1
		**CTGCCATATTGTAGCTCAATA**		SI00046200	Hs_SAFB_3
**SAFB**	NM_002967	**ACGGACTGTAGTAATGGATAA**	scaffold attachment factor B	SI00046186	Hs_SAFB_1
		**CCGAAGATGACTCGGATACAA**		SI00046207	Hs_SAFB_4
		**AAGGAGTATATTGATGAGGAA**		SI00710542	Hs_SAP130_2
**SAP130**	NM_024545	**CAGGAAATAGCTAATCAGAAA**	Sin3A-associated protein, 130kDa	SI00710535	Hs_SAP130_1
		**CAGGTGGAAATGAGTTCTCAA**		SI00710549	Hs_SAP130_3
		**ACGGGTCTTCACCACCAATAA**		SI03041780	Hs_SAP18_5
**SAP18**	NM_005870	**AGCCACGGGATTGTATGAAAT**	Sin3A-associated protein, 18kDa	SI00710563	Hs_SAP18_1
		**GAGCGTATGGTTAAGCCCAAA**		SI03103149	Hs_SAP18_6
		**GACGTGGAATTGAGACTAATA**		SI00710598	Hs_SAP30_2
**SAP30**	NM_003864	**CCAGAGGTTGATTTATACCAA**	Sin3A-associated protein, 30kDa	SI00710591	Hs_SAP30_1
		**AAAGCACAACTTGTTGAGATA**		SI00710605	Hs_SAP30_3
		**CACTCTTATTGTTGTATGCAA**		SI00046305	Hs_SATB1_2
**SATB1**	NM_002971	**TCCAAGCAATTCATACTATAA**	SATB homeobox 1	SI00046298	Hs_SATB1_1
		**ACCGAATATACCAGGACGAAA**		SI00046312	Hs_SATB1_3
		**TTGGGATACCAGATAGCTCAA**		SI00715757	Hs_SET_3
**SET**	NM_003011	**CAGGAATCTTGCTCCAATAAA**	SET translocation (myeloid leukemia-associated)	SI00715743	Hs_SET_1
		**AAGAAGATAGGCTCTCAGTAA**		SI00715764	Hs_SET_4
		**CACGCCTAACTGCTACGCCAA**		SI00453187	Hs_KIAA0339_2
**SETD1A**	NM_014712	**CTGGACGAGTTCTATATTGGA**	SET domain containing 1A	SI00453194	Hs_KIAA0339_3
		**CAGCGTATTATGAAAGCTGGA**		SI00453180	Hs_KIAA0339_1
		**TAGGGCCAGGGTATTATTATA**		SI00138901	Hs_SET7_1
**SETD7**	NM_030648	**CACCTGGACGATGACGGATTA**	SET domain containing (lysine methyltransferase) 7	SI00138915	Hs_SET7_3
		**CAGGGAGTTTACACTTACGAA**		SI00138908	Hs_SET7_2
		**CTGCAGTCTGAAGAAAGGAAA**		SI02664991	Hs_SET8_7
		**AAGAATAGATGAATTGATTGA**		SI02664998	Hs_SET8_8
**SETD8**	NM_020382	**TGGGTTAATTCTTTATAGAAA**	SET domain containing (lysine methyltransferase) 8	SI00126007	Hs_SET8_2
		**CTGCAGTCTGAAGAAAGGAAA**		SI02664991	Hs_SET8_7
		**TGGGTTAATTCTTTATAGAAA**		SI00126007	Hs_SET8_2
		**AAGAATAGATGAATTGATTGA**		SI02664998	Hs_SET8_8

**SETDB1**	NM_012432	**ACCCATGGGATTGCAATTAAA**<br>**ACCGAAAGTTTCAGCCCTATA**<br>**TCGGGTGGTCGCCAAATACAA**	SET domain, bifurcated 1	SI00100338<br>SI00100352<br>SI00100345	Hs_SETDB1_1<br>Hs_SETDB1_3<br>Hs_SETDB1_2
**SETDB2**	NM_031915	**TACCAGGTTTAGAATGGTATA**<br>**CCAGTGATATTCTGCAATCAA**<br>**CCGAGAGCATCTGAACTCTAA**	SET domain, bifurcated 2	SI00141386<br>SI00141379<br>SI00141393	Hs_SETDB2_3<br>Hs_SETDB2_2<br>Hs_SETDB2_4
**SGCA**	NM_000023	**CTCCTGGAACCCAGGCTCTAA**<br>**CAGGCTCTAAACAAGCAGGGA**<br>**CAGGTCCAGCCCTGACTTCAT**	sarcoglycan, alpha (50kDa dystrophin-associated glycoprotein)	SI02622767<br>SI02622760<br>SI02622774	Hs_SGCA_3<br>Hs_SGCA_2<br>Hs_SGCA_4
**SGCG**	NM_000231	**CCGACCCGTTTCAAGACCTTA**<br>**CCGTGTGGAGTTAATGTATGA**<br>**AGGCGCTTTCTCAAATGGATA**	sarcoglycan, gamma (35kDa dystrophin-associated glycoprotein)	SI00004977<br>SI00004970<br>SI03045217	Hs_SGCG_2<br>Hs_SGCG_1<br>Hs_SGCG_5
**SHPRH**	NM_001042683 NM_173082	**CAGAGCGATCTATGAAAGCAA**<br>**GTGGGATTAATCGATGGTGTA**<br>**AGGGTCTGAAACTCTACTATA**	SNF2 histone linker PHD RING helicase	SI00157913<br>SI03107125<br>SI03046106	Hs_SHPRH_3<br>Hs_SHPRH_6<br>Hs_SHPRH_5
**SIN3A**	NM_015477	**CTGGTCACTCGAGCACATCTA**<br>**GAGCGTGTAAGCAAGCGTCTA**<br>**CAGAGGATATATGCTGATAAA**	SIN3 homolog A, transcription regulator (yeast)	SI00719047<br>SI02781240<br>SI00719068	Hs_SIN3A_1<br>Hs_SIN3A_5<br>Hs_SIN3A_4
**SIN3B**	NM_015260	**CAGCTATGTGAATAAGATTAA**<br>**CACATTAAAGTTAATAATTGA**<br>**CAAGATGGTGTTCATCGTGAA**	SIN3 homolog B, transcription regulator (yeast)	SI00719082<br>SI00719075<br>SI00719089	Hs_SIN3B_2<br>Hs_SIN3B_1<br>Hs_SIN3B_3
**SIRT1**	NM_012238	**CAAGCGATGTTTGATATTGAA**<br>**TTGGGTCTTCCCTCAAAGTAA**<br>**CAGGATTATTGTATTTACGTT**	sirtuin (silent mating type information regulation 2 homolog) 1 (S. cerevisiae)	SI00098434<br>SI00098448<br>SI00098441	Hs_SIRT1_1<br>Hs_SIRT1_3<br>Hs_SIRT1_2
**SIRT2**	NM_012237	**AAACTCCACATCCGCAGGCAT**<br>**ACCGGCCTCTATGACAACCTA**<br>**AATCTCCACATCCGCAGGCAT**	sirtuin (silent mating type information regulation 2 homolog) 2 (S. cerevisiae)	SI00301805<br>SI00098406<br>SI02655471	Hs_SIRT2_5<br>Hs_SIRT2_1<br>Hs_SIRT2_6
**SIRT3**	NM_001017524 NM_012239	**CTGTGCCTAGTTGAACGGCAA**<br>**CAGGATGTAGCTGAGCTGATT**<br>**AAGGTGGAAGAAGGTCCATAT**	sirtuin (silent mating type information regulation 2 homolog) 3 (S. cerevisiae)	SI00098476<br>SI00098469<br>SI00098483	Hs_SIRT3_3<br>Hs_SIRT3_2<br>Hs_SIRT3_4
**SIRT4**	NM_012240	**TTGGGTTATTTGTGCCAGCAA**<br>**CCAGCGGTACTGGGCGAGAAA**<br>**CACCTGGGAGAAACTCGGAAA**	sirtuin (silent mating type information regulation 2 homolog) 4 (S. cerevisiae)	SI00098497<br>SI00098490<br>SI00098504	Hs_SIRT4_2<br>Hs_SIRT4_1<br>Hs_SIRT4_3
**SIRT5**	NM_012241 NM_031244	**CTGGAGATCCATGGTAGCTTA**<br>**TCGATTGATTTCCCAGCTATA**<br>**CCGCCTCAAGCATTAGAACTA**	sirtuin (silent mating type information regulation 2 homolog) 5 (S. cerevisiae)	SI00098532<br>SI00098518<br>SI00098525	Hs_SIRT5_3<br>Hs_SIRT5_1<br>Hs_SIRT5_2
**SIRT6**	NM_016539	**CACGGGAACATGTTTGTGGAA**<br>**CTCCCTGGTCTCCAGCTTAAA**<br>**GTCGAGGATGTCGGTGAATTA**	sirtuin (silent mating type information regulation 2 homolog) 6 (S. cerevisiae)	SI00116592<br>SI02777698<br>SI00116599	Hs_SIRT6_1<br>Hs_SIRT6_5<br>Hs_SIRT6_2
**SLBP**	NM_006527	**ATCAGTTAATTTACCATGTAA**<br>**TACGATCGTTATATTAAAGAA**<br>**TTGCCTTACTTTCTTAAGAAA**	stem-loop (histone) binding protein	SI00719670<br>SI00719663<br>SI00719677	Hs_SLBP_2<br>Hs_SLBP_1<br>Hs_SLBP_3
**SMARCA1**	NM_003069 NM_139035	**CTCGATTCAATTGTTATACAA**<br>**CCCGAATAAAGAAAGATGAAA**<br>**CAGTGTATTCATGGTACTCTA**	SWI/SNF related, matrix associated, actin dependent regulator of chromatin, subfamily a, member 1	SI00726719<br>SI00726733<br>SI00726726	Hs_SMARCA1_1<br>Hs_SMARCA1_3<br>Hs_SMARCA1_2
**SMARCA2**	NM_003070 NM_139045	**AGGCGTCTACATAAGGTGTTA**<br>**AAGGATGACGCTGAAGTAGAA**<br>**GCCCGCATAGCTCATAGGATA**	SWI/SNF related, matrix associated, actin dependent regulator of chromatin, subfamily a, member 2	SI00726747<br>SI00726761<br>SI00726754	Hs_SMARCA2_1<br>Hs_SMARCA2_3<br>Hs_SMARCA2_2
**SMARCA4**	NM_003072	**CCGCGCTACAACCAGATGAAA**<br>**CCCGTGGACTTCAAGAAGATA**<br>**TCACTGGATGTCAAACAGTAA**	SWI/SNF related, matrix associated, actin dependent regulator of chromatin, subfamily a, member 4	SI00047579<br>SI02630208<br>SI00047586	Hs_SMARCA4_1<br>Hs_SMARCA4_5<br>Hs_SMARCA4_2
**SMARCA5**	NM_003601	**CTCAATTGTTATGTCATTTAA**<br>**CTGGATTCAATAGTCATTCAA**<br>**AACCAGTAGTTCTTTAATTTA**	SWI/SNF related, matrix associated, actin dependent regulator of chromatin, subfamily a, member 5	SI00726775<br>SI00726789<br>SI00726782	Hs_SMARCA5_1<br>Hs_SMARCA5_3<br>Hs_SMARCA5_2
**SMARCAD1**	NM_020159	**AAGGGTGATAGAGTTGTGTTA**<br>**TCGGAATTGGAAGACCTTAAA**<br>**TTGGCTGGCATTGGTACATAA**	SWI/SNF-related, matrix-associated actin-dependent regulator of chromatin, subfamily a, containing DEAD/H box 1	SI02778314<br>SI00124999<br>SI00124992	Hs_SMARCAD1_5<br>Hs_SMARCAD1_2<br>Hs_SMARCAD1_1
**SMARCAL1**	NM_014140	**CAGCTTTGACCTTCTTAGCAA**<br>**CAGAACAGCATCAGAGGACTA**<br>**TTGAGTTATGAGTTAGGTCAA**	SWI/SNF related, matrix associated, actin dependent regulator of chromatin, subfamily a-like 1	SI00103180<br>SI00103201<br>SI00103194	Hs_SMARCAL1_1<br>Hs_SMARCAL1_4<br>Hs_SMARCAL1_3
**SMARCB1**	NM_001007468 NM_003073	**ACCAGAGAAGTTTGCCCTGAA**<br>**CCGGCTGGACATGGAGATCGA**<br>**GCGCGTCATCATCAAGCTGAA**	SWI/SNF related, matrix associated, actin dependent regulator of chromatin, subfamily b, member 1	SI00726817<br>SI00726803<br>SI00726810	Hs_SMARCB1_3<br>Hs_SMARCB1_1<br>Hs_SMARCB1_2
**SMARCC1**	NM_003074	**AAGGAGAGCATCCTAAGTTAA**<br>**CTGGCAGAACATACCACTGAA**<br>**AAGAATGGTGCTGATGGTAAA**	SWI/SNF related, matrix associated, actin dependent regulator of chromatin, subfamily c, member 1	SI00726831<br>SI00726845<br>SI00726838	Hs_SMARCC1_1<br>Hs_SMARCC1_3<br>Hs_SMARCC1_2
**SMARCC2**	NM_003075 NM_139067	**CCGAAAGAAGATTTCAGCCAA**<br>**CCAAACTACTAGGGAAATTAA**<br>**AAGAAGTATATACAAGCTGAA**	SWI/SNF related, matrix associated, actin dependent regulator of chromatin, subfamily c, member 2	SI00726859<br>SI00726873<br>SI00726866	Hs_SMARCC2_1<br>Hs_SMARCC2_3<br>Hs_SMARCC2_2
**SMARCD1**	NM_003076 NM_139071	**TCCAAATATGATGCCACTAAA**<br>**CAACAGGAGATTGCTACTCTA**<br>**AAGCACTGTGGCAATATATTA**	SWI/SNF related, matrix associated, actin dependent regulator of chromatin, subfamily d, member 1	SI00726887<br>SI00726908<br>SI00726894	Hs_SMARCD1_1<br>Hs_SMARCD1_4<br>Hs_SMARCD1_2
**SMARCD2**	NM_001098426 NM_003077	**GCGGGAGTACATCAACTGCAA**<br>**CCAGAAGAAGACAGCCTGTTA**<br>**CCGGATCGACAAATCCCGGAA**	SWI/SNF related, matrix associated, actin dependent regulator of chromatin, subfamily d, member 2	SI00726915<br>SI00726929<br>SI00726922	Hs_SMARCD2_1<br>Hs_SMARCD2_3<br>Hs_SMARCD2_2
**SMARCD3**	NM_001003801 NM_001003802 NM_003078	**GTGGCAGTATGTGAAGACCAA**<br>**CAGGACTCCCATGACAAGGAA**<br>**CTCAAGGTGATGACAGATGTA**	SWI/SNF related, matrix associated, actin dependent regulator of chromatin, subfamily d, member 3	SI00047621<br>SI00047607<br>SI00047628	Hs_SMARCD3_3<br>Hs_SMARCD3_1<br>Hs_SMARCD3_4
**SMARCE1**	NM_003079	**CCCGCGTACCTTGCTTACATA**<br>**AAGTTGTGGGAGATTGGCAAG**<br>**AAGGAATTTAATATTCTTGTA**	SWI/SNF related, matrix associated, actin dependent regulator of chromatin, subfamily e, member 1	SI02655107<br>SI00301840<br>SI02656458	Hs_SMARCE1_6<br>Hs_SMARCE1_1<br>Hs_SMARCE1_7
**SMC1A**	NM_006306	**CACCATCACACTTTAATTCCA**<br>**CGGCGTATTGATGAAATCAAT**<br>**ATGGAGGAGATTCGTAAGAAA**	structural maintenance of chromosomes 1A	SI02655219<br>SI00087248<br>SI00087241	Hs_SMC1L1_5<br>Hs_SMC1L1_2<br>Hs_SMC1L1_1
**SMYD1**	NM_198274	**CACGTTTGTTATCTCTGCCTA**<br>**CGGGATGATCTGCAAAGCCTA**<br>**CAACGTTAGTGAAGAACGCAA**	SET and MYND domain containing 1	SI00727608<br>SI00727594<br>SI03053239	Hs_SMYD1_4<br>Hs_SMYD1_2<br>Hs_SMYD1_5
**SMYD2**	NM_020197 XM_001127274	**GAGGTTAGTCTGAATCTTGAA**<br>**CCGGAGGGCCAAGCACTATAA**<br>**AAGATAGAAATGACCGGTTAA**	SET and MYND domain containing 2	SI00125335<br>SI00125328<br>SI00125342	Hs_SMYD2_2<br>Hs_SMYD2_1<br>Hs_SMYD2_3
**SMYD3**	NM_022743	**CACGATTATAATAAATTCAAA**<br>**CTGGAGTCAAATATTAACAAA**<br>**CAGCTGCCTTGCAGTAATTAA**	SET and MYND domain containing 3	SI00727615<br>SI00727622<br>SI00727629	Hs_SMYD3_1<br>Hs_SMYD3_2<br>Hs_SMYD3_3
**SNW1**	NM_012245	**CCCGATGAAGAAGCTATTAAA**<br>**AACGTCTGGCTGCTGATGGAA**<br>**TCGGATGGTAGAAATGCAGAA**	SNW domain containing 1	SI02655478<br>SI00301812<br>SI00098609	Hs_SKIIP_7<br>Hs_SKIIP_5<br>Hs_SKIIP_2
**SORT1**	NM_002959	**CTGGGTTTGGCACAATCTTTA**<br>**CAGCAGAGAATTGACTAGATA**<br>**CAAGGACAGGGTACAAACTTA**	sortilin 1	SI00729309<br>SI00729295<br>SI00729316	Hs_SORT1_3<br>Hs_SORT1_1<br>Hs_SORT1_4
**SP100**	NM_003113	**AACAATCTTGTCATACTTGAA**<br>**CTGGTGGTATTTAGCCACTAA**<br>**CCCAGGGAATTTGAAATTGAA**	SP100 nuclear antigen	SI00729876<br>SI00729869<br>SI00729855	Hs_SP100_4<br>Hs_SP100_3<br>Hs_SP100_1
**SSRP1**	NM_003146	**AAGCGAGAGCTTCAAGAGCAA**<br>**CAGGCGAGATCTGGAAGGGAA**<br>**TTCGTTGACTCTGAACATGAA**	structure specific recognition protein 1	SI00733810<br>SI00733817<br>SI00733803	Hs_SSRP1_2<br>Hs_SSRP1_3<br>Hs_SSRP1_1
**SUDS3**	NM_022491	**CTGCAGGTGATTACTTTGAAA**<br>**CAGATGAATTTCCAAGTTTAA**<br>**GAGAATCAGCATAGTAAATTA**	suppressor of defective silencing 3 homolog (S. cerevisiae)	SI00713370<br>SI00713363<br>SI00713377	Hs_SDS3_2<br>Hs_SDS3_1<br>Hs_SDS3_3
**SUV39H1**	NM_003173	**CAGGTGTACAACGTCTTCATA**<br>**GCGGGTCCGTATTGAATGCAA**<br>**CAGAAACTTTGTACTAGTGAA**	suppressor of variegation 3-9 homolog 1 (Drosophila)	SI02665019<br>SI00048664<br>SI00048671	Hs_SUV39H1_6<br>Hs_SUV39H1_1<br>Hs_SUV39H1_2
**SUV39H2**	NM_024670	**AAGCGTTAAGCTGATAATGTA**<br>**CGCGGAGGATATGGAATATTA**<br>**CACAGTGGATGCGGCTCGATA**	suppressor of variegation 3-9 homolog 2 (Drosophila)	SI02665026<br>SI02665033<br>SI03056410	Hs_SUV39H2_5<br>Hs_SUV39H2_6<br>Hs_SUV39H2_7
**SUV420H1**	NM_016028 NM_017635	**AACCATGATTGCAGACCTAAT**<br>**TGGGTCCTGCTGCGTTTATAA**<br>**CTGGTGGGTTGTATTGCCGAA**	suppressor of variegation 4-20 homolog 1 (Drosophila)	SI00119091<br>SI03021032<br>SI00119084	Hs_SUV420H1_4<br>Hs_SUV420H1_7<br>Hs_SUV420H1_3
**SUV420H2**	NM_032701	**CAGCACGAGAACTGTGCGAGA**<br>**CATCATGTACTCAACCCGCAA**<br>**CCGGCGCTATGGGCTGCCTTA**	suppressor of variegation 4-20 homolog 2 (Drosophila)	SI03065503<br>SI03073714<br>SI00144060	Hs_SUV420H2_5<br>Hs_SUV420H2_6<br>Hs_SUV420H2_1
**TADA2L**	NM_001488 NM_133439	**CCAGGTTAAAGGAGAGACAAA**<br>**AGCGATCATACTTATGAAATA**<br>**ACCTTAGAAAGTTTCAATTAA**	transcriptional adaptor 2 (ADA2 homolog, yeast)-like	SI00738486<br>SI00738479<br>SI00738493	Hs_TADA2L_2<br>Hs_TADA2L_1<br>Hs_TADA2L_3
**TAF1**	NM_004606 NM_138923	**CCCAATTTGTTCATATTTGTA**<br>**ATGAAAGAACTAGTAACTTTA**<br>**TAGTAAGTAAATGATAATAAA**	TAF1 RNA polymerase II, TATA box binding protein (TBP)-associated factor, 250kDa	SI00738542<br>SI00738549<br>SI00738535	Hs_TAF1_2<br>Hs_TAF1_3<br>Hs_TAF1_1
**TAF10**	NM_006284	**CAGCACCGCCGCGGAGAACAA**<br>**CACCTGAGCCACCCAACCTAA**<br>**CCAGAAATTCATCTCAGATAT**	TAF10 RNA polymerase II, TATA box binding protein (TBP)-associated factor, 30kDa	SI00086898<br>SI00086884<br>SI02637152	Hs_TAF10_4<br>Hs_TAF10_2<br>Hs_TAF10_5
**TAF12**	NM_005644	**CAGGGAATGGACAGCAATGTA**<br>**CTCCAATTTCTCATCCATAAA**<br>**CCAGAATAAAGAAGTTATTAA**	TAF12 RNA polymerase II, TATA box binding protein (TBP)-associated factor, 20kDa	SI00738605<br>SI00738598<br>SI00738612	Hs_TAF12_3<br>Hs_TAF12_2<br>Hs_TAF12_4
**TAF4B**	NM_005640 XM_290809 XM_937014	**AAGAGTCGTTCTAATAAAGAA**<br>**AAGAATGACTATTTCATTCTA**	TAF4b RNA polymerase II, TATA box binding protein (TBP)-associated factor, 105kDa	SI00738850<br>SI00738843	Hs_TAF4B_2<br>Hs_TAF4B_1

	XM_937014	**AAGGATTTGGAAGAAAGAGAA**		SI00738857	Hs_TAF4B_3
		**CAGGATGACCTACGAGTATTA**		SI00738878	Hs_TAF5_2
**TAF5**	NM_006951	**ACCTCAGTGATTAATATTTAA**	TAF5 RNA polymerase II, TATA box binding protein (TBP)-associated factor, 100kDa	SI00738871	Hs_TAF5_1
		**TTGGAGCTAGTCTACAATCAA**		SI00738885	Hs_TAF5_3
		**CTGCAATACCAGTCTAATGAA**		SI00738913	Hs_TAF5L_3
**TAF5L**	NM_014409	**CTGGGACATCAGGAACACTTA**	TAF5-like RNA polymerase II, p300/CBP-associated factor (PCAF)-associated factor, 65kDa	SI00738906	Hs_TAF5L_2
		**CTGGGATGTTCAGTCATGAAA**		SI00738920	Hs_TAF5L_4
		**CAGCGTGCAGCCCATCGTCAA**		SI00738934	Hs_TAF6_2
**TAF6**	NM_005641 NM_139122 NM_139123 NM_139315	**ACCAAGTTTATTGTAATGTCA**	TAF6 RNA polymerase II, TATA box binding protein (TBP)-associated factor, 80kDa	SI00738927	Hs_TAF6_1
		**CCGTGTGAACGTGGTTCAGAA**		SI00738941	Hs_TAF6_3
		**CCGCACTTTCGTTTCATCATA**		SI00738962	Hs_TAF6L_2
**TAF6L**	NM_006473	**CAGGCTGTGCTGGATGATTAT**	TAF6-like RNA polymerase II, p300/CBP-associated factor (PCAF)-associated factor, 65kDa	SI00738955	Hs_TAF6L_1
		**CCCTTCGTTCCTTGTAAATAA**		SI00738969	Hs_TAF6L_3
		**ATCACTGATAGTAGCATTTAA**		SI00739074	Hs_TAF9L_2
**TAF9B**	NM_015975	**TAGGCTGAAGTCCTTAATTAA**	TAF9B RNA polymerase II, TATA box binding protein (TBP)-associated factor, 31kDa	SI00739067	Hs_TAF9L_1
		**CAGAAGTTTCATCTTAGGATA**		SI00739081	Hs_TAF9L_3
		**CCACTTGAAACAATAAATAAA**		SI00748846	Hs_TNKS1BP1_2
**TNKS1BP1**	NM_033396	**CCGGAGAGTTCCTTAAATCAA**	tankyrase 1 binding protein 1, 182kDa	SI00748839	Hs_TNKS1BP1_1
		**ACGGATGTCGTTGGGCACCAA**		SI00748853	Hs_TNKS1BP1_3
		**AACGAAGTTTGGAAACAGAAA**		SI00749546	Hs_TOPBP1_2
**TOPBP1**	NM_007027	**ACGAGTATACAGAGACCTTAA**	topoisomerase (DNA) II binding protein 1	SI00749553	Hs_TOPBP1_3
		**CAGCAATATTTCCAACATAAA**		SI00749539	Hs_TOPBP1_1
		**CAGGAGTGATTTCTCTTTATA**		SI00320537	Hs_C14orf92_1
**TOX4**	NM_014828	**CACCAAGTTGATTATTACCAA**	TOX high mobility group box family member 4	SI00320551	Hs_C14orf92_3
		**CAGAAAGTTCGAATCAATTTA**		SI00320558	Hs_C14orf92_4
		**AGCGTTATCGCCTACTTGGAA**		SI00372260	Hs_DNMT2_2
		**CACATTCGGTTGAGCAACATT**		SI00372253	Hs_DNMT2_1
**TRDMT1**	NM_004412 NM_176081 NM_176083 NM_176084 NM_176085 NM_176086	**CTGCGATATTTCACTCCTAAA**	tRNA aspartic acid methyltransferase 1	SI00372274	Hs_DNMT2_4
		**AGCGTTATCGCCTACTTGGAA**		SI00372260	Hs_DNMT2_2
		**CTGCGATATTTCACTCCTAAA**		SI00372274	Hs_DNMT2_4
		**CACATTCGGTTGAGCAACATT**		SI00372253	Hs_DNMT2_1
		**TAGGTGTACTGTACTGAAGAA**		SI00056798	Hs_TIF1_3
**TRIM24**	NM_003852 NM_015905	**CCGAGACTTATCTAAACCAGA**	tripartite motif-containing 24	SI00056805	Hs_TIF1_4
		**CACGACAAGCAATAAAGCCAA**		SI00056791	Hs_TIF1_2
		**CTGGCCCTATTCTGTCACGAA**		SI00750757	Hs_TRIM28_3
**TRIM28**	NM_005762	**AGCGTCCTGGCACTAACTCAA**	tripartite motif-containing 28	SI03144988	Hs_TRIM28_5
		**CAGGAGATGATCCCTACTCAA**		SI00750750	Hs_TRIM28_2
		**TCCCGTAGTATCGGTACACAA**		SI02777740	Hs_TRIM33_5
**TRIM33**	NM_015906 NM_033020	**AAGGGTGCAATTGAGAATCTA**	tripartite motif-containing 33	SI00112455	Hs_TRIM33_1
		**CTGGGCAAAGAATGTAGTCAA**		SI02777747	Hs_TRIM33_6
		**AAGGTATTTATTATTACCAGA**		SI00751317	Hs_TRIM66_3
**TRIM66**	XM_084529 XM_940455	**CCCGGCATTATTACCAGATTA**	tripartite motif-containing 66	SI00751303	Hs_TRIM66_1
		**AGAGAAGATTTCAGTAATAAA**		SI00751310	Hs_TRIM66_2
		**AACAAGCTGATTGTCAAGGAA**		SI00752178	Hs_TSPYL1_2
**TSPYL1**	NM_003309 XM_371844	**AACAACTTAGTAAGTGATATA**	TSPY-like 1	SI00752171	Hs_TSPYL1_1
		**AAGCAGGAGAAACTGAACAAA**		SI00752185	Hs_TSPYL1_3
		**CAAGGTGATAGTGAAGGAGTA**		SI00752213	Hs_TSPYL3_3
**TSPYL3**	NM_178465 NR_002781	**TAGGACAGGTTGCAAATTCAA**	TSPY-like 3 (pseudogene)	SI00752199	Hs_TSPYL3_1
		**TAGGAGCTTCATCATCCAGAA**		SI00752206	Hs_TSPYL3_2
		**CTGATTGGGCTTCATACTTTA**		SI00752234	Hs_TSPYL4_2
**TSPYL4**	NM_021648	**TAGGTTAATTGTTTAGTTATA**	TSPY-like 4	SI00752227	Hs_TSPYL4_1
		**TTGGTTCTAAATCTTCAGTAA**		SI00752241	Hs_TSPYL4_3
		**AACCTGTATCTCTGTTATAAA**		SI00752269	Hs_TSPYL5_3
**TSPYL5**	NM_033512	**CCCGGACTGTTCATATGTTAA**	TSPY-like 5	SI00752255	Hs_TSPYL5_1
		**CAAGATTGTGGAGATAATCAA**		SI00752262	Hs_TSPYL5_2
		**CCCGAAGAAGAAGACTACCAA**		SI04173568	Hs_VCX_8
**VCX**	NM_013452	**CACTGGGAGAAGGAAGTGAGA**	variable charge, X-linked	SI00759570	Hs_VCX_2
		**AAAGCAGTTCGTAGAGGGAGA**		SI04197074	Hs_VCX_11
		**AAAGCAGTTCGTAGAGGGAGA**		SI03122014	Hs_VCX3A_5
**VCX3A**	NM_016379 XM_001127089 XM_001127103 XM_001127115	**TAGTGGAGTGTTGACCAATCA**	variable charge, X-linked 3A	SI04359586	Hs_VCX3A_12
		**CAAGGCCACGGAGGCAGGAAA**		SI03157574	Hs_VCX3A_6
		**AAGAGTCTCCACCACAGATAA**		SI00504903	Hs_LOC344760_2
**VN2R1P**	XM_293581 XM_941234	**TAGCGGAGATTGGCATATGTA**	vomeronasal 2 receptor 1 pseudogene	SI00504896	Hs_LOC344760_1
		**TCGCCAGTTACCAGATAATTA**		SI00504910	Hs_LOC344760_3
		**CCCAGTGACCTTCATGTCCAA**		SI00763798	Hs_XAB1_2
**XAB1**	NM_007266	**CAGAGTCTCCTTGGCAATCAA**	XPA binding protein 1, GTPase	SI00763791	Hs_XAB1_1
		**CACACTGTATAGCTCATTATA**		SI00763805	Hs_XAB1_3
		**CCGATACGTCACAGCCTTCAA**		SI00087157	Hs_XRCC1_1
**XRCC1**	NM_006297	**AAGCCTGAAGTATGTGCTATA**	X-ray repair complementing defective repair in Chinese hamster cells 1	SI00087178	Hs_XRCC1_4
		**CCGATGGATCTACAGTTGCAA**		SI02665117	Hs_XRCC1_5
		**GCCGAAGATTATGAAATGAAA**		SI00765422	Hs_ZBTB33_2
**ZBTB33**	NM_006777	**TAGGAACTACTTGAAATAGTA**	zinc finger and BTB domain containing 33	SI00765429	Hs_ZBTB33_3
		**AAGAATTAAATGCTAACTTAA**		SI00765415	Hs_ZBTB33_1
		**CACGGTACTCTCCATCTTAAA**		SI00408842	Hs_FLJ35036_3
**ZBTB38**	NM_001080412 XM_001133510 XM_001133669 XM_172341	**ACCGAAATTCTTCCTATGAAA**	zinc finger and BTB domain containing 38	SI00408828	Hs_FLJ35036_1
		**CTGGACGATCTCAAAGCTGAA**		SI00408835	Hs_FLJ35036_2
		**AAGAATACATATATATATATA**		SI00765478	Hs_ZBTB4_2
**ZBTB4**	NM_020899	**TCCCTAATTTGCACTAGTTAA**	zinc finger and BTB domain containing 4	SI00765492	Hs_ZBTB4_4
		**CACAAATTATATATCTATCTA**		SI00765471	Hs_ZBTB4_1
		**ACAGAGCGTGTTGTCCGAGAA**		SI03037706	Hs_ZMYND11_5
**ZMYND11**	NM_006624 NM_212479	**ACCGAAGTTATGAAGAGTTCA**	zinc finger, MYND domain containing 11	SI03039694	Hs_ZMYND11_6
		**CCGGATGAAGTCGGACCACAA**		SI00090412	Hs_ZMYND11_1
		**CACCATTGAACAGTTATCCTA**		SI00288064	Hs_PRKCBP1_8
**ZMYND8**	NM_012408 NM_183047 NM_183048	**CCGGCAGCATCCTGAATCTTA**	zinc finger, MYND-type containing 8	SI00163485	Hs_PRKCBP1_5
		**AGCGAACTTGTCATAGATTTA**		SI00288057	Hs_PRKCBP1_7
