# Supplementary material for: A Broad Set of Chromatin Factors Influences Splicing
Source: PLoS Genet. 2016 Sep 23;12(9):e1006318. doi: 10.1371/journal.pgen.1006318 (PMC5035054; doi:10.1371/journal.pgen.1006318)
Supplement: S3 Table — Chromatin factors identified in both screens are highlighted in yellow; Non-overlapping siRNAs are highlighted in red or green. (PDF) [file pgen.1006318.s007.pdf]

Table S3: Comparative analysis of chromatin factors retained as hits in Salton et al. and our work.

Comparative analysis of siRNA screens

Salton et al.	allemand et al.			
ARID5A	HAT1	H2AFY2	NPM2	TSPYL4
BRDT	AOF2	HDAC2	PHC1	XAB1
DDX5	ASH1L	HDAC9	PHC2	ZMYND11
EHMT2	ATAD2	HIST1H1D	POLE3	
EIF3S6	BAP1	HIST1H2AB	PRM2	
EP300	BAZ1A	HIST1H2AL	RFC1	
HMGN2	BAZ1B	HIST1H3B	SETDB2	
HMGN4	BPTF	HIST1H3G	SIN3A	
ING1L/ING2	BRD1	HIST1H4A	SMARCA2	
JUND	BRD7	HIST2H2AA3	SMARCA5	
MLL2/MLL4	CBX3	HMGB3	SMARCAD1	
MTA2	CHD5	ING2	SMARCC1	
PCAF	CHD7	LOC389168	SMARCD1	
RAN	CHRAC1	MLL4	SMYD2	
RENT1	DNMT1	MORF4L2	SUDS3	
SAFB	EHMT1	MTA3	TADA2β	
SCMH1	EZH2	MYST1	TAF1	
SMCHD1	FBXL11	NAP1L4	TAF10	
SUV39H2	GTF3C4	NASP	TAF5	
TIF1/TRIM24	H2AFX	NCOA1	TRIM33	

Comparative analysis of siRNA screen (Salton et al.) and Proteomic (Allemand et al.)

Salton et al.	allemand et al.				
ARID5A	PBRM1	HDAC2	SNW1	HIST1H4L	BRIP1
BRDT	SMARCC2	WBP7	SAFB	HIST3H2BB	TOX4
DDX5	SMARCA4	KAT6B	NCOR2	HIST1H1T	LIG4
EHMT2	SMARCD1	EHMT1	SIN3A	H2AFY2	
EIF3S6	BRD7	ZMYND8	GATAD2A	HIST1H2BA	
EP300	SMARCC1	BRD8	NCOR1	MTA2	
HMGN2	CHD4	MLL3	ZBTB4	ACIN1	
HMGN4	ACTL6A	MLL2	HP1BP3	RBBP4	
ING1L/ING2	SMARCE1	HDAC1	DMAP1	SMC1A	
JUND	SMARCA1	SETD1A	SIN3B	MBD3	
MLL2/MLL4	SMARCD2	SHPRH	TAF1	PARP4	
MTA2	SMARCA2	MORF4L1	RB1	NASP	
PCAF	SMARCB1	EHMT2	COBRA1	C14ORF43	
RAN	ARID1A	BAZ1B	CBX3	BRCA2	
RENT1	CHD7	DOT1L	ZBTB33	MSL3	
SAFB	BPTF	CDYL	TRIM28	POLE3	
SCMH1	BRD1	MBD3L2	C6ORF130	SMARCA5	
SMCHD1	CBX2	EIF2C1	EIF2C2	HERC2	
SUV39H2	CHD5	PRMT8	TSPYL1	BRCA1	
TIF1/TRIM24	ARID1B	PRMT5	TNKS1BP1	DICER1	

Legend:
- (red) Hits from Salton et al. not present in the siRNA library of Allemand et al.
- (light green) Hits from Allemand et al. not present in the siRNA library of Salton et al.
- (yellow) Hits in common between the two studies
